# Ten years of pluviometric analyses in Italy for civil protection purposes

**DOI:** 10.1038/s41598-021-99874-w

**Published:** 2021-10-13

**Authors:** Matteo Del Soldato, Ascanio Rosi, Luca Delli Passeri, Carlo Cacciamani, Filippo Catani, Nicola Casagli

**Affiliations:** 1 Earth Sciences Department, University of Firenze, Via La Pira, 4, 50121 Firenze, Italy; 2 Department of Civil Protection, Presidency of the Council of Ministers, Via Vitorchiano 2, 00189 Rome, Italy; 3 Regional Agency for Prevention and Environmental of Emilia-Romagna Region - Idrometeoclima service, Viale Silvani, 6, 40122 Bologna, Italy; 4 Department of Geosciences, University of Padova, Via G. Gradenigo, 6, 35131 Padova, Italy; 5 National Institute of Oceanography and Applied Geophysics, Borgo Grotta Gigante, 42/C, 34010 Sgonico, TS Italy

**Keywords:** Environmental sciences, Natural hazards

## Abstract

The concept of climate change has grown in recent decades, influencing the scientific community to conduct research on meteorological parameters and their variabilities. Research on global warming, as well as on its possible economic and environmental consequences, has spread over the last 20 years. Diffused changes in trends have been stated by several authors throughout the world, with different developments observed depending on the continent. Following a period of approximately 40 days of almost continuous rain that occurred from October to November 2019 across the Italian territory and caused several hazards (e.g., floods and landslides), a relevant question for decision-makers and civil protection actors emerged regarding the relative frequencies of given rainfall events in the Warning Hazard Zones (WHZs) of Italy. The derived products of this work could answer this question for both weather and hydrogeological operators thanks to the frequency and spatio-temporal distribution analyses conducted on 10-year daily rainfall data over the entire Italian territory. This work aspires to be an additional tool used to analyse events that have occurred, providing further information for a better understanding of the probability of occurrence and distribution of future events.

## Introduction

The concept of climate change has grown in recent decades, and this growth has prompted the scientific community to investigate meteorological variables and their variabilities. Global warming and its possible economic and environmental consequences have been largely investigated over the last 20 years. One of the most crucial parameters taken into consideration regarding the socioeconomic and environmental consequences of global warming is the spatio-temporal distribution of precipitation ^1–4^.

In literature, on the one hand, several works have stated that in Northern Europe, *e.g.,* in Ireland^5–7^, in Central and Northern Asia, *e.g.*, in the Himalayan region in the period 1943 and 1993^8^ large-scale incremental precipitation trends was identified. A similar trend was recognized also in the Eastern part of North America, *e.g.,* in North Carolina^9^ and Canada^10^, from 1940s to the 1990s. On the other hand, in Central and Northern Asia, *e.g.* central Sahel^11^, in the Southern African and Southern Asia areas, *e.g.,* the Yellow River Basin^25^, the expansion of drying conditions and decreasing annual precipitation have been exhibited. For a more limited timespan, from 1961 to 1990, a reduction of annual rain was recorded also in Nigeria^12^, but the area to which these differences in the last decades were mainly accented is the Mediterranean region^13–24^. Conversely, the short and very intense storms, recently characterizing the Mediterranean region, are often cause of landslide and flood events with serious socioeconomic consequences and considerable losses in terms of human life^26–30^.

Focusing on Italy, when studying the temporal distribution of rainfall, increases in the maximum rainfall values were recorded in North and Central Italy^31,32^; however, when considering annual precipitation at the regional scale, several authors have recognized decreasing trends^33–45^ or no relevant changes^46–48^.

**Figure 1:**
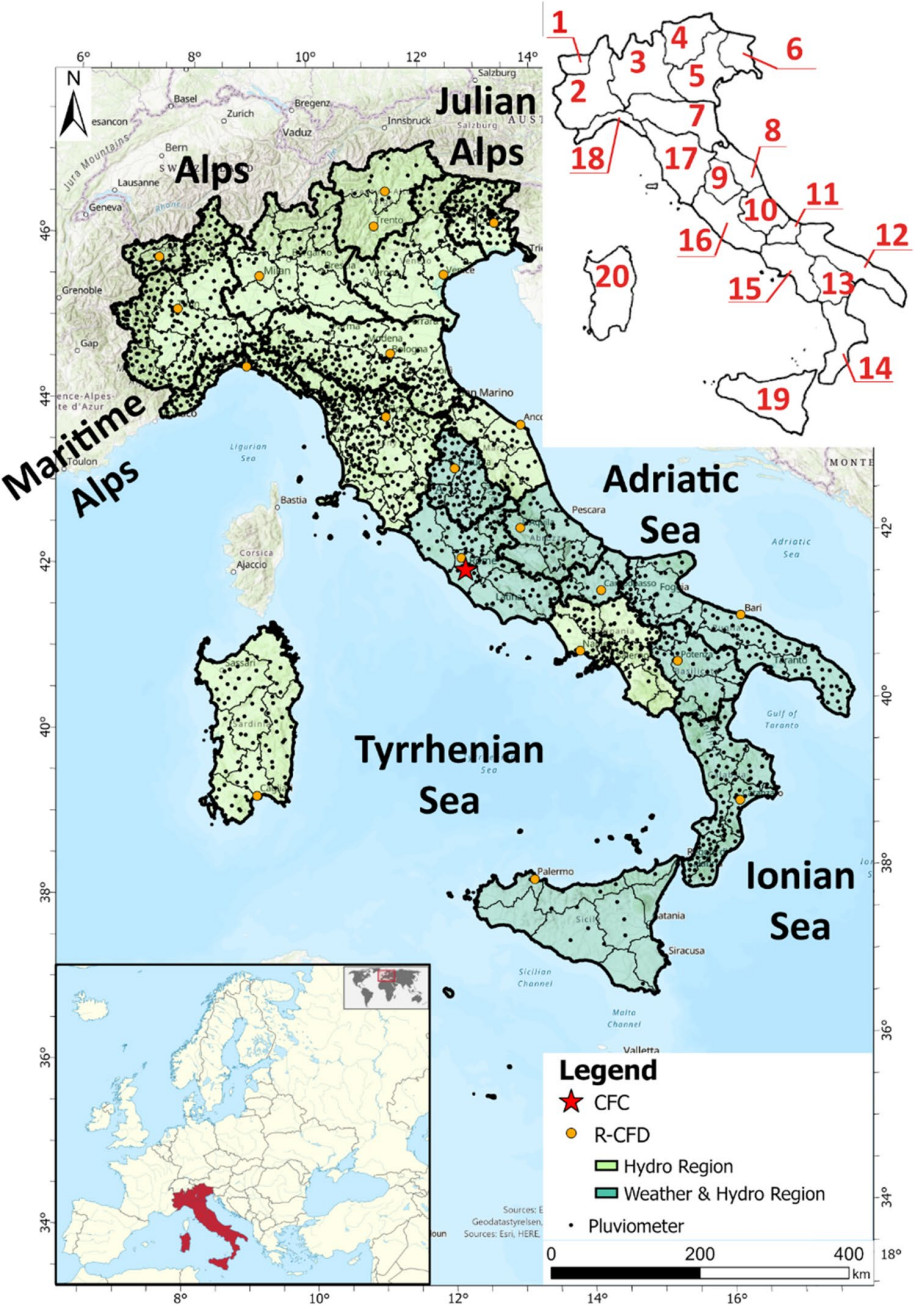
Localization of the area of interest with discrimination of the Regional Decentralized Functional Centre (R-CFD) zones that are autonomous only for hydrogeological warnings (in light green) and the R-CFD zones that are autonomous for both weather forecasting and hydrogeological warnings (in dark green). The map was generated using ESRI ArcGIS Pro 2.5.0 (https://www.esri.com/en-us/home). 1-Valle d’Aosta; 2-Piemonte; 3-Lombardia; 4-Trentino-Alto Adige; 5-Veneto; 6-Friuli-Venezia Giulia; 7-Emilia-Romagna; 8-Marche; 9-Umbria; 10-Abruzzo; 11-Molise; 12-Puglia; 13-Basilicata; 14-Calabria; 15-Campania; 16-Lazio; 17-Toscana; 18-Liguria; 19-Sicilia; 20-Sardegna Regions. In the first image, the Central Functional Centre (CFC) in Rome and the locations of all rain gauges analysed in the presented study are also reported with black dots.

Investigations of the rainfall distribution over the Italian territory play a fundamental role due to the implications of considerable hydrogeological problems severely affecting the national territory. Italy is strongly and frequently affected by natural hazards, such as subsidence^49^, landslides^50^ and floods in flat areas^51,52^ or particular environments^53,54^, and these hazards have relevant socioeconomic consequences^55–58^. The ISPRA (Superior Institute for Environmental Protection and Research—*Istituto Superiore per la Protezione e la Ricerca Ambientale*) conducts various studies on this topic investigating long series of 30-years precipitation data (1951–1980 and 1961–1990). The consequences of rainy events in Italy are often significant, for this reason this issue is dealt by Center of Competence and coordinated by the Italian Civil Protection Department of the Presidency of the Council of Ministers (DPC). Meteorological and hydrogeological warnings related to the rain are demanded to the Regional Functional Centre (RFC) offices coordinated by the Central Functional Centre (CFC) office of the DPC. These offices, in addition to delivering daily weather bulletins, have the task of assessing and monitoring the rainfall and hydrological situation over their corresponding regional territory (RFC) of Italy (CFC). The Italian territory is administratively divided into 20 Regions, each with a corresponding RFC office for managing the regional hydrogeological warning system. Twelve regions comprising northern and central Italy are completely autonomous regarding both their warning and weather forecasting systems (Hydro Region, light green in Fig. 1), while 8 regions in central and southern Italy are independent only regarding their warning systems but depend on the meteorological centre of the DPC for their weather forecasting systems (Weather & Hydro Region, dark green in Fig. 1a). All RFCs are able to manage hydrogeological warnings in their territory thanks to knowledge of the criticality and strength of these hazards.

Aside from the published investigations of rainfall frequency analysis, which are commonly made while considering a period of 30 years, an analysis of 10-year data covering the entire Italian territory was performed in this study due to the availability of a relevant number of spatially distributed rain gauges over Italy. Kiely et al.^6^ affirmed that the rainfall with high precipitation rate is increasing and that the probability of the return period of relevant events is shortening from 30 to 10 years. The analysis was conducted for all 158 Warning Hydrogeological Zones (WHZs) in Italy; these zones are reference areas for geological and hydraulic risk alerts and are delimited considering their morphology, catchment boundaries and administrative limits and used for issuing hydrogeological warnings.

This work was idealized after a prolonged rainy period that occurred in Italy from October to November 2019, with a period of approximately 40 days of almost continuous rainfall all over the country. This prolonged rainfall event led to several hazards, such as floods and landslides, in different regions. The analysis of this period posed an important question for decision-makers and for the personnel involved in weather-forecasting and alert-issuing activities: what is the relative frequency of a given rainfall event in each WHZ of Italy? The abilities to characterize rainfall events after their occurrence and to define their probabilities of occurrence with respect to the events of the last 10 years have been considered primary issues for operative staff since these factors are useful for increasing the knowledge operators have regarding their territory.

The results presented in this work can be useful for both weather and hydrogeological analyses since this work considers the spatio-temporal distribution of daily rainfall data in the last 10 years over the entire Italian territory and also analyses of the rainy frequency in each WHZ. In addition, the resulting information can be used by different actors working at different levels of the hydrogeological management chain for civil protection. The obtained results can also be useful for the CFC and RFC operators acting in weather forecasting and monitoring fields because the entire procedure can be systematically repeated in a relatively short time.

The products derived in this study can fulfil the request of obtaining information about rainfall events for the entire territory of Italy, for a single Region in Italy or for a specific WHZ. The final purposes of this study are to obtain an additional tool for analysing events that can also provide useful information for a better understanding of future events and their distribution and probability of occurrence.

## Results

### Spatial distribution of the average rainfall

A dense network of spatially distributed rain gauges over Italy provides continuous direct observations of rain measurement with high time resolution (up to 1 min) for several specific locations^59^. The Civil Protection Department, more specifically the CFC, has the opportunity to continuously register pluviometric data over the whole Italian territory using a distributed network composed of more than 4500 rain stations. From 2010, more than 2000 rain gauges were selected to compose a robust database of rainfall data with good spatial and temporal distributions to investigate the national and WHZ-specific 10-year rainfall frequency. This network is spread across the Italian territory considering its physiography and geomorphology, allowing the collection of rainfall measurements approximatively every 15 min or each hour. The rain gauge stations take part in a robust and complete instrument network composed of rain gauges, thermometers, hydrometers, and hygrometers used by the CFC of the DPC and by the RFC to monitor the weather and hydrogeological conditions.

**Figure 2:**
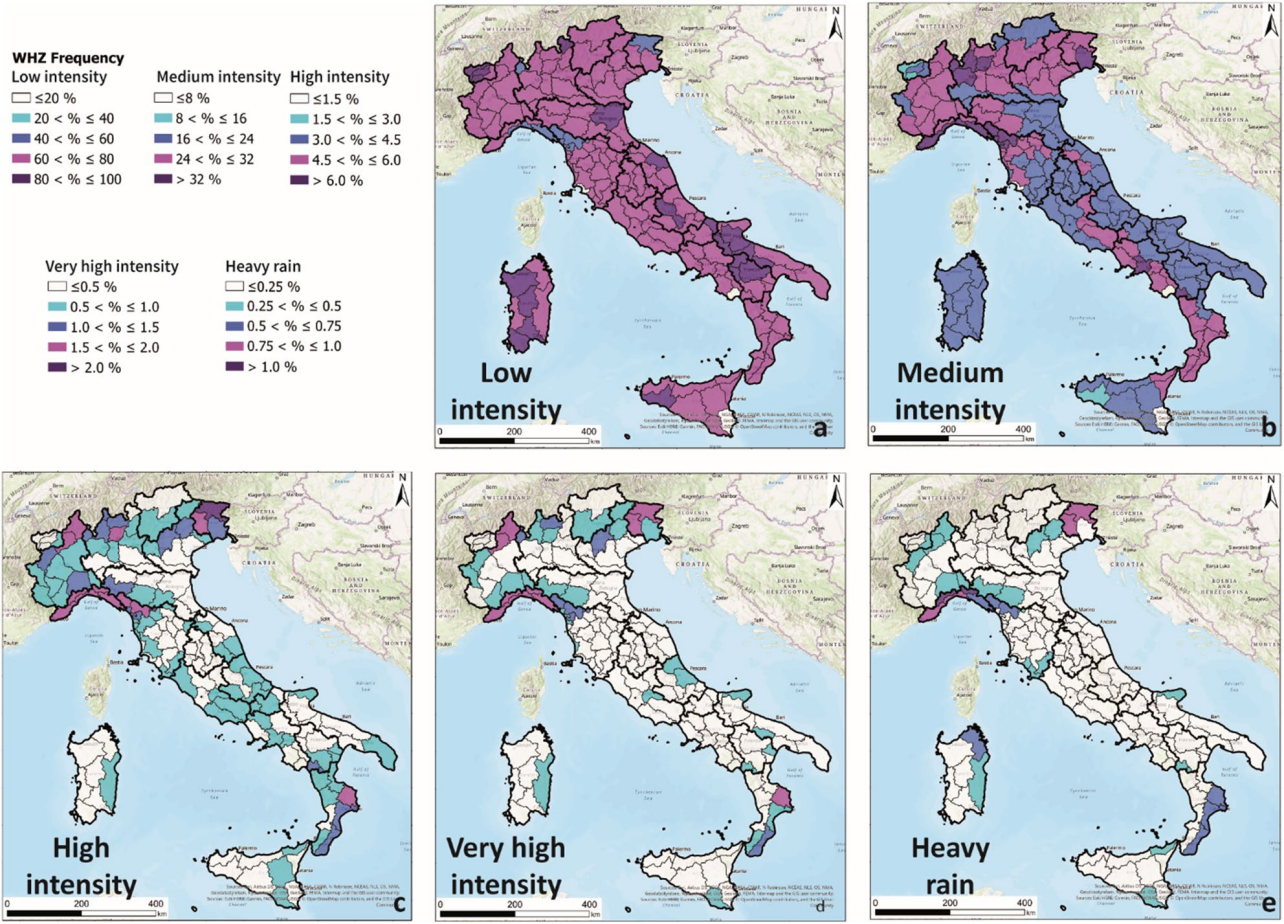
Spatial distribution of the rainfall frequency for each Warning Hydrogeological Zone (WHZ) from 1 January 2010 to 31 December 2019 over the entire Italian territory. The maps were generated using ESRI ArcGIS Pro 2.5.0 (https://www.esri.com/en-us/home). The maps show the spatial distributions of the rainfall frequencies falling within the Low (**a**), Medium (**b**), High (**c**), Very high (**d**) and Heavy rain (**e**) intensity classes.

For civil protection purposes, knowledge of the daily distribution of rain is fundamental for the issuance of national weather and hydrogeological risk bulletins. The possibility of having an updated rainfall distribution covering the entire Italian territory would allow easier comparative analyses between average rainfall values and forecasted rainfall data. Considering all available rain station data over the Italian territory (2041 rain gauges–black dots in Fig. 1), the rainfall frequency and the spatial distributions of rainfall were mapped from 1 January 2010 to 31 December 2019 (Fig. 2).

For this purpose, the rain data were ranked into five severity classes according to the DPC classification:

-  < 20 mm of daily rain—“Low intensity” (Fig. 2a)
- 20—60 mm of daily rain—“Medium intensity” (Fig. 2b)
- 60—100 mm of daily rain—High intensity (Fig. 2c)
- 100—150 mm of daily rain—“Very high intensity” (Fig. 2d)
-  > 150 mm of daily rain—“Heavy rain” (Fig. 2e)

Generally, the spatial distributions of the “Low intensity” (Fig. 2a) and “Medium intensity” (Fig. 2b) rates do not display relevant differences in the Warning Hydrogeological Zones of the entire Italian territory, with the exception of the northern part Tuscany Region and the Liguria Region, where the frequency of “Medium intensity” rainfall is higher than that of the “Low intensity” rainfall.

The spatial distribution of “High intensity” rainfall (Fig. 2c), in contrast, highlights the alpine sectors of Italy, spanning from the southwestern Alps (named the Maritime Alps) to the Friuli-Venezia Giulia Region (north-eastern Alps, also called the Julie Alps), where higher numbers of days with rainfall rates between 60 and 100 mm were recorded with respect to the rest of the country. In the two abovementioned areas, rainfall frequencies between 4.5 and 6% and higher than 6% were assessed.

A similar distribution can be found in the “Very high intensity” map (Fig. 2d). The frequency of rainfall classified as “Very high intensity” showing rates between 1.5 and 2% turns out more localized in the WHZs of the Liguria Region, in the northern WHZs of the Piemonte Region and in the WHZs of the eastern portion of the Calabria Region (the Ionian seacoast).

Investigating the “Heavy rain” class, the situation is similar to the spatial distribution of the “Very high intensity” class, but the “Heavy rain” class turns out more localised. It can be point out also considering that in many cases no rate is recorded in the borer WHZs. A clear example can be the one of the Calabria Region in which the Ionian seacoast WHZs show values higher than 1% and the closest Tyrrhenian WHZs exhibit negligible rates. The same situation can be recognized in the north-eastern portion of the Sardegna Region. The northern WHZs of the Piemonte Region do not exhibit a significant frequency percentage for this class, while four WHZs of the Liguria Region and three of the Friuli-Venezia Region are characterized by values higher than 0.75% and 1% (Fig. 2e).

For four WHZs, Camp-8, Sici-G, Tosc-E3, and Tosc-I, no rainfall information could be recorded because the rain stations of these WHZs were omitted by the application of the filters to collect and examine a robust. The rain gauges of these areas recorded too few rainy data and the minimum functionality threshold were not satisfied.

It is interesting to note that the WHZs that are characterized by high occurrence frequencies of the “High intensity”, “Very high intensity” or “Heavy rain” classes follow the distribution of the main damages recorded in the last decade. The WHZs of the Friuli Venezia Giulia Region are characterized by high rainfall frequencies, as confirmed by several socioeconomic losses and casualties registered in the last decade (OCDPC-Ordinances of the Head of the Civil Protection Department-555/2018). In recent decades, more than 27 landslides and 38 flooding events resulted in funds being required for urgent remedial works^60^.

Comparing the alpine sector, the northern WHZs of the Piemonte Region feature high frequencies of the “High intensity” and “Very high intensity” class\es. These areas were characterized by approximately 300 new landslide events that occurred in the last decade as the consequences of high levels of rain^61^. No relevant rainfall frequencies were highlighted in the WHZs close to the Liguria Region, where relevant flood events have recently filled the newspapers and induced the administrative office to apply for a state of emergency and request funding of approximatively 440 million euros to remedy the consequences and socioeconomic damages caused by 88 landslides and 53 floods events^60^ (*e.g.*, OCDPC 181/2014-OCDPC 269/2015-OCDPC 430/2017-OCDPC 534/2018-OCDPC 615/2019-OCDPC 620/2019). The entire territory of the Liguria Region, especially the northern WHZs, registered high relevant rainfall frequencies in the “High intensity” classes. These territories are well known due to the high numbers of damaging events that have been recorded in the last decade due to both flood and landslide events. The flood events recorded in Liguria are: (i) floods occurred in 2010, 2011, 2014 and 2019 in Genova town and province, (ii) in 2011 in the Cinque Terre areas of La Spezia province and (iii) in 2014 and 2019 in the western provinces and in the areas close to the border with the Piemonte Region. The latest relevant consequences of these events included the collapse of the Polcevera viaduct, also known as “Ponte Morandi”^62–69^, and the collapse of the Savona-Torino bridge on the A6 highway^70^.

East of the Liguria Region, the WHZs of northern Toscana Region also show relevant rainfall rates (more than 1.5% and more than 1%, respectively) with very high frequencies. Both areas are characterized by mountainous territories, and the relevant consequences of these rainfall events are reflected as high numbers of landslides; in fact, approximately 220 landslides were recorded in recent decades in this area. The area was seriously affected by landslides several times over the last decade with serious consequences in terms of socioeconomic losses and some casualties. Significant events occurred in 2010, 2014^71^, 2017 and 2019 (*e.g.*, OCDPC 203/2014-OCDPC 216/2014-OCDPC 434/2017-OCDPC 485/2017-OCDPC 621/2019-OCDPC 622/2019).

In general, the whole southern Italy is characterized by high rainfall frequencies in the “Low intensity” and “Medium intensity” classes and low rainfall frequencies in the other rain intensity classes. The Ionian coast of the Calabria Region shows significant frequencies in both “High intensity” and “Very high intensity” classes. For this reason, the Ionian WHZs of the Calabria Region stand out. In 2015 in the southern region and in 2018 in the northern area, relevant floods occurred, causing significant consequences. The ReNDiS data^60^ state that 32 landslide and 17 flood events required national funds of approximately 12 and 26 million euros, respectively, for the associated damages.

Another region that exhibits relevant frequencies in the “Heavy rain” class is the WHZ on the north-eastern coast of the Sardegna Region (OCDPC 360/2016, OCDPC 366/2016). In this region, 20 recorded flood events have caused high costs of approximately 59 million euros due to the consequential damages^60^.

### 10-year rainfall frequency analysis for the warning hydrogeological zones

**Figure 3:**
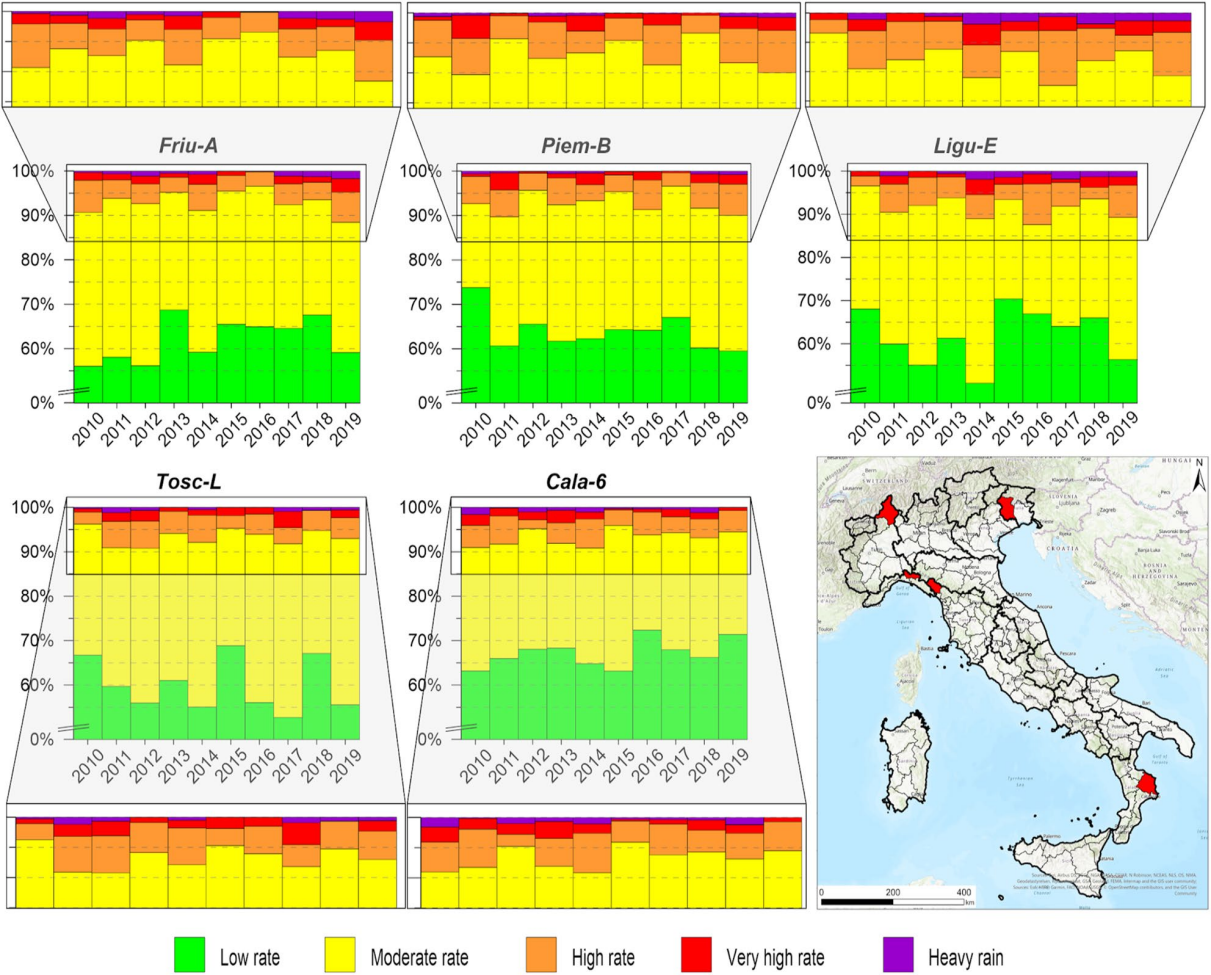
Pluviometric frequency analysis for five WHZs characterized by relevant values in all intensity classes: Friu-A, Piem-B, Ligu-E, Tosc-L and Cala-6. The map was generated using ESRI ArcGIS Pro 2.5.0 (https://www.esri.com/en-us/home). For the higher intensity classes, zoomed images are provided for better visualization.

In addition to the average frequencies per rainfall class, a possible evolution of the frequency was investigated for each warning hydrogeological zone. In Fig. 3, five of the most significant identified frequency revealing the situation and evolution of the rain are reported: (i) Friu-A in the north-western Friuli Venezia Giulia Region; (ii) Piem-B in the north-western Piemonte Region, close to the Valle d’Aosta Region; (iii) Ligu-E in the north-eastern Liguria Region; (iv) Tosc-L in the northern Tuscany Region, close to Liguria: (v) Cala6 in the eastern Calabria Region, facing the Ionian Sea. All these WHZs have the same relevant frequencies of the “Very high intensity” and “Heavy rain” classes.

For each year, the frequencies of the “Low intensity”, “Medium intensity”, High intensity, “Very high intensity” and “Heavy rain” classes are displayed to illuminate the frequency in the last 10 years (Suppl. 1). The “Low intensity” class ranges from approximately 44% in Ligu-C, the eastern WHZ of the Liguria Region, to 93% in Sici-D, in the south-western Sicilia Region. Basi-D, in the western Basilicata Region, showed 100% of the investigated rainfall data categorized in the “Low intensity” class in 2011. The same result was found for the Lomb-01 WHZ, in the northern Lombardia Region, while for Tosc-O3, in the southern Toscana Region, showed 100% of the investigated rainfall data classified in the “Low intensity” class in 2017. The “Medium intensity” class resulted in a wider category, with values between approximately 6.7% registered in Sici-D, in the south-western Sicilia Region, and 49% recorded in Tosc-S2, in north-west of Tuscany. The High intensity category ranged between 0.1%, assessed in Pugl-C in the eastern Puglia Region, and 11.5% in Lomb-01, in the northern Lombardia Region. No rainfall events falling within the “Very high intensity” class were recorded over the entire study period in Sici-C in the southern Sicilia Region, Sici-E in the northern Sicilia Region, or Sici-I in the north-eastern Sicilia Region, or in Lomb-13 in the south-western Lombardia Region or Sard-G in the north-western Sardegna Region. The “Heavy rain” class showed a minimum frequency of 0.03% in Camp-3 and a maximum frequency of two orders of magnitude higher, approximately 3.5%, recorded in Sici-F in the southern Sicilia Region in 2019. This class exhibited the largest number of no recorded events, with 35 WHZs recording no events with daily rainfall higher than 150 mm for the entire study period.

The zoomed panels on the upper portions of the graphs of Fig. 3 show as no clear trends could be recognized in the rainy frequency analysis. This result can be justified by considering that a database covering only 10 years was available and analysed.

Notably, in the frequency analysis, due to the restrictions put in place to obtain a robust dataset, some WHZ results had “*no data*” for some years:

- 2010 showed “no data” values for Basi-D in the western Basilicata Region, Lomb-03 in the north-western Lombardia Region, Piem-E in the western Piemonte Region and Vene-A in the northern Veneto Region;
- 2014 exhibited “no data” for Tosc-S2 and Tosc-V, both in the north-western Toscana Region;
- 2015, 2016, 2017, 2018 and 2019 showed “no data” values for the Sici-D zone in the southwestern Sicilia Region.

## Discussion

The availability of data from several rain stations over the entire Italian territory is a very useful tool for the investigation, monitoring and back-analysis of rainfall conditions. These analyses can be used to compare historical data with forecasted data, giving direct estimations of the severity of the expected rain event and allowing better estimations of the possible consequences. Some of the rainfall stations have long time series, but the instruments often have problems and are substituted with other instruments located not precisely in the same place. This operation can affect the number of rain stations with long time series. In fact, only a small number of rain gauges had registered data over very long periods. According to ISPRA documents, areas with the highest recorded rainfall amounts are the alpine and pre-alpine sectors of the Friuli-Venezia Giulia (above 2000 mm/year), the northern portion of Piemonte, the Apuan Alps, located in northern Toscana, and eastern Liguria. The lowest average annual rainfall rates were recorded in the southern portion of Sicilia, Puglia and southern Sardegna^72^.

In view of this information (defined in the period from 1961–1990), the obtained rainfall distribution maps considering the period from 2010–2019 confirm a similar situation. In fact, areas with higher frequencies of relevant events, considering the “Very high intensity” (100—150 mm/h) and “Heavy rain” classes (> 150 mm/h), trace quite well the results highlighted for the reference period from 1961–1990. The main difference can be identified on the Ionian coast of the Calabria Region due to sporadic but relevant events that occurred in 2015 and 2018; these events also caused some flood events with significant socioeconomic consequences.

A comparison between the maps of average precipitation of 30-years (1961–1990) realized by the ISPRA research institute, and classified according to the presented work, and the areas characterized by “Very high intensity” and “Heavy rain” of the period 2010–2019 (Fig. 2a and b, respectively) was made. It was possible to identify:

- The South of Italy is featured by average values of precipitation of the period 1961–1990 between 20–60 and 60–100 mm/day except for one WHZ of the Basilicata Region (Basi-C) and some WHZs of the Campania Region. The last 10-year analysis shows, differently, the highest classes in the Ionian seacoast the WHZs of the Calabria Region. No high values were recorded in the WHZs of the Puglia Regions, while a correspondence can be recognized for the Basi-C WHZ. Considering the islands, there is a quite good correspondence for the Sicilia Region, while the Sardegna Region exhibits an increment of rain in the last ten-years in the eastern WHZs;
- The Central Italy is the portion of Italy where the main differences were recognized. The period 1961–1991 exhibits generally an average precipitation between 60–100 mm/day with the western Apennine WHZs characterized with values between 60 and 100 mm/day with sporadic WHZs with values 100–150 mm/day in the Adriatic Apennine line. The last 10-years shows the higher classes for few WHZs only in the south of Tuscany and some two WHZs in the Marche Regions. A good correspondence is highlighted in the northern WHZs of the Tuscany region where in both periods considered, significant rates of precipitations were recorded;
- The North Italy exhibits a very good correspondence for the two periods with the areas with highest daily average precipitations in the Alpine chain with emphasis in the Liguria Region, northeastern portion of the Alps (Valle d’Aosta and Piemonte Regions) and the Julian Alps (Friuli Region). Slight differences can be recognized in the eastern portion of the Liguria Region (Ligu-C) where in the last decade significant events characterized by high intensity occurred, that was already recognized in the period 1961–1990 because featured by significant rainy rates.

For the 10-year precipitations a frequency analysis was conducted considering all the rain classes (Fig. 3 and Suppl. 1), and for the “Very high intensity” and “Heavy rain” classes jointly (Suppl. 2A) and only for “Heavy Rain” class (Suppl. 2B) show no significant variations in frequency either together or separated in the investigated period. Slight changes in frequency can be identified only for few WHZs. This result can be useful for focusing future research on these areas and for monitoring the conditions in the whole territory. The absence of a relevant frequency distribution could be due to the period investigated, which was ten years in this study, since a period of at least 30 years is usually considered for climate change analyses. In any case, the rainfall distribution and frequency class distribution can be considered relevant since the probability of the return period of relevant events featuring high precipitation intensities is reduced from 30 to 10 years^6^.

The period of 10-year is no longer with respect the usual 30-year period used for the frequency analysis and trend investigation. In this work, only 10-years were considered because the available databases did not allow having information for more years. The few data available before the 2010 did not pass the data quality checking and filtering, for this reason this work was focused on ten-year frequency investigation.

**Figure 4:**
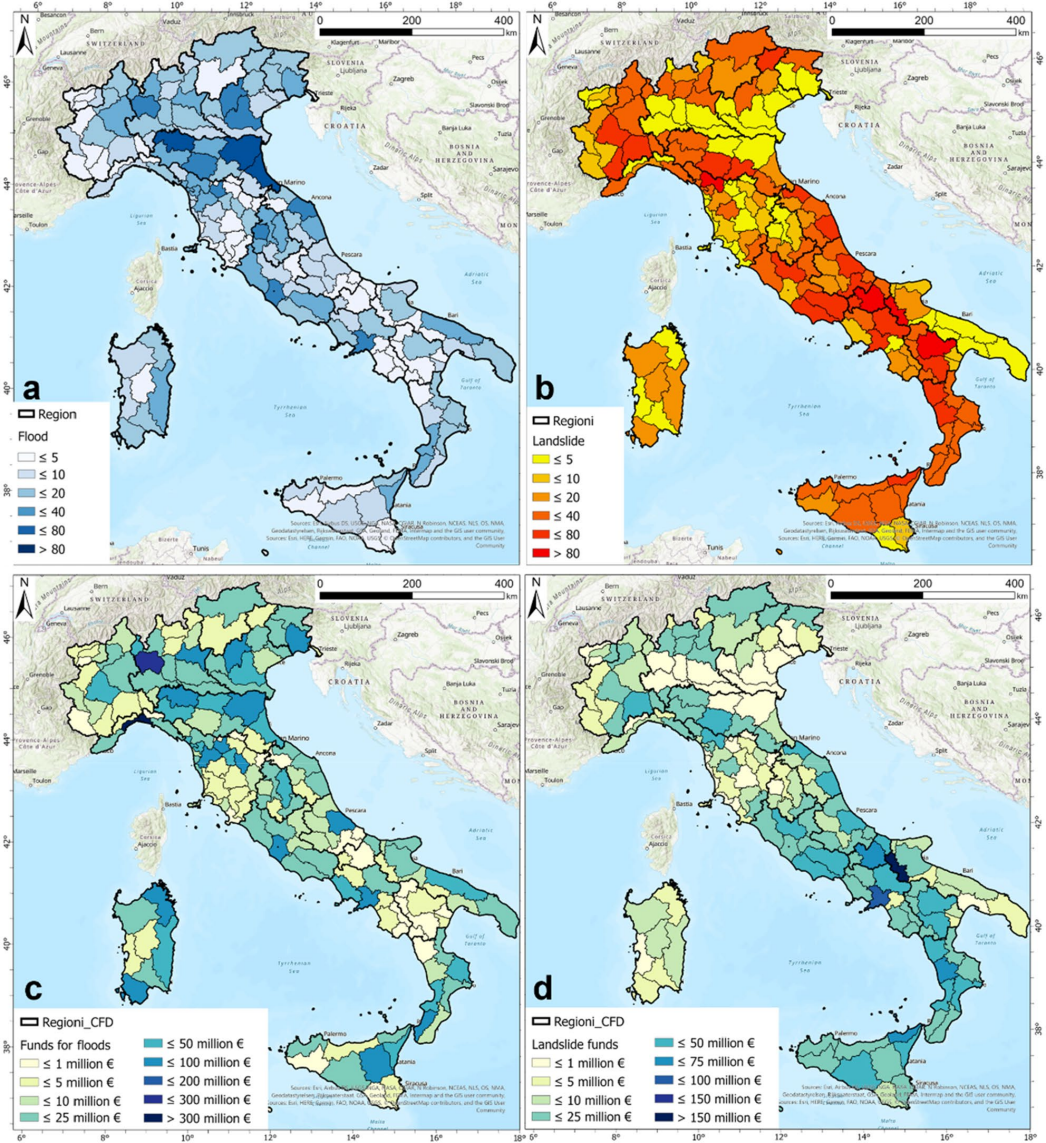
Number of damaging events (a, floods; b, landslides) and funds allocated for remediation works (c, floods; d, landslides). (data obtained from ISPRA^60^).

The frequencies of daily rainfall higher than 100 mm (“Very high intensity”) and 150 mm (“Heavy rain”) together (Suppl. 2A) and only for the rainfall higher than 150 mm (“Heavy rain”) (Suppl. 2B) in the 2010—2019 period were compared with the ReNDiS (National Repository of Soil Defense interventions—*Repertorio Nazionale degli interventi per la Difesa del Suolo*) database^60^. This qualitative comparison was useful to verify if the areas highlighted as rainiest correspond to the areas most involved by natural hazards. ReNDiS is a database of remediation work planned to restore the damage derived from natural events such as landslides and floods, founded by the Italian government. The ReNDiS data begins in 2000 and covers the period until 2019; in this dataset, it was reported that more than 3.2 billion euros and 2.2 billion euros were allocated as funding to remediate the damages caused by flood^74^ and landslide events, respectively. The distributions of the numbers of flood (Fig. 4a) and landslide (Fig. 4b) events trace the situation highlighted through the analysis of the rain gauge data for the 2010–2019 period, since the regions where higher frequencies of high-intensity rainfall occurred were also the regions with higher numbers of reported events. A comparison of the flood and rainfall frequency distributions shows a good correspondence for the Sardegna Region and Ionian seacoast, but the flood and rainfall frequencies do not seem to match in the Po basin, specifically in the territory of the Emilia-Romagna Region. By taking into consideration the overflow time and the huge contribution of the wide Po basin, comprising the Piemonte and Lombardia Regions, this discrepancy can be explained. In fact, floods in the Pianura Padana Plain in the Emilia Romagna Region occur due to accumulated and overflowing rain in the entire basin. Rainfall events falling within the “High intensity” and “Very high intensity” classes were recorded in the Piemonte and Lombardia alpine arcs. The correspondence between the landslide and rainfall frequencies, instead, is simpler and more recognizable. Some examples of good matching can be observed in the Liguria Region, mainly in the coastal WHZs, in the Apuane Alps in northern Toscana Region, and in the north-western portion of the alpine arc, *i.e.*, the Friuli-Venezia Giulia and Trentino-Alto Adige Regions. Some differences can be justified considering that the ReNDiS product^60^ takes into consideration urgent interventions over a longer period, from 2000 to 2019, compared with the dataset used in this study, and the ReNDiS dataset does not provide the dates of the events the works refer to. It is worth noting that the allocated funds for both flood events (Fig. 4c) and slope instabilities (Fig. 4d) almost coincide well with the areas involved in events due to the relevant frequencies of rainfall recorded by the rain gauge network for the investigated period of 2010–2019.

The good correlation between the rainfall distribution for the 2010–2019 period and the ReNDiS data requires the consideration of the managing actors of the hydrogeological risk. A comparison between the 20-year^60^ ReNDiS data and the 10-year extracted information highlights that the areas in which more interventions were required were subjected to higher frequencies of rain events with relevant intensities (> 100 mm/day). This result should induce the relevant actors to pay more attention to these areas not only for repair-related interventions after damaging events but also for prevention, forecasting and monitoring actions. This work could help in this purpose by serving all civil protection and hydrogeological risk managing actors.

A possible future development of this work could be the analysis of sub-daily rain frequency. This could be possible taking advantage of the availability of hourly rainy data for a relevant number of rain gauges. This type of analysis will allow to investigate the high intensity event with low duration, e.g. few hours, that more and more often causes relevant socioeconomic damages and a higher number of casualties due to fast landslides or flash floods. This work was the first result of the analysis conducted on a big amount of rainy data and it was useful to analyse the frequency of rainfall in the last ten-years. A future insight will be conducted on the same data for identify a procedure for investigating rainfalls by sub-daily timespan, e.g. 1-h, 3-h and 6-h cumulated and rate.

## Methods

**Figure 5:**
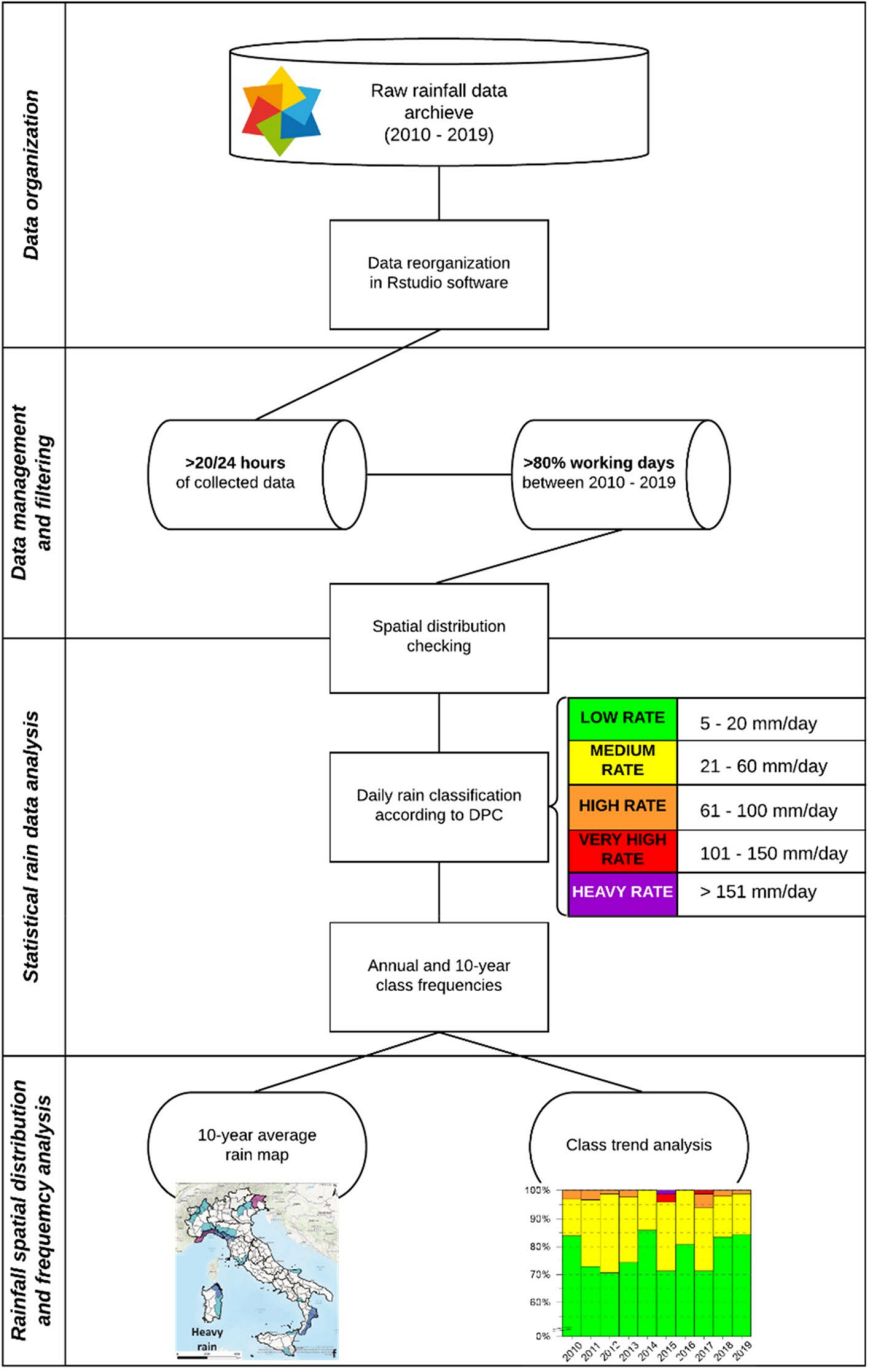
Flowchart of the adopted methods in the investigation and frequency analysis. The flowchart was generated using Lucidchart (https://www.lucidchart.com/pages/).

The applied approach (Fig. 5) involved taking into consideration only the rain stations that collected relevant databases of rain values and was based on singular to two-step filtering of the available daily and 10-year rain gauges data with subsequent classification and spatialization steps to design rainfall maps and analyse the frequency class distribution over the 2010–2019 period. The raw rainfall data were elaborated through a 3-step process:

1. rain gauges data were downloaded and organized;
2. data preparation was conducted by means of data quality checking and filtering;
3. statistical rain data analysis was performed by DPC classification and WHZ categorization;
4. the spatial distribution of rainfall was obtained, and a frequency analysis was performed.

The entire procedure of selecting data from the databases was realized by a script developed in Rstudio software^77^.

### Rain gauge data downloading and organization

A database composed of 4500 spatially distributed rain gauges was available from the myDewetra^75^ portal developed by CIMA foundations^76^ as a zip file for each month containing a .csv file for each rain station. Each .csv file contains a table with two columns: the first column represents the time of acquisition, in yyyymmddhhmm format; the second column reports the hourly cumulative rainfall. In the first lines, the name, coordinates, and elevation (m a.s.l.) of the rainfall station are indicated. The first operation was to reorganize the database to collecting the daily rainfall values of all the available rain stations in a table and to report the name and the relative coordinates of each station.

### Rain data management and filtering

All rain gauges were singularly analysed to select only the rain gauges that recorded data for more than 20 h per day to set up a database that was as robust as possible from a statistical point view by removing noisy data and data with low representativeness. In addition to considering only the rain stations that recorded consistent rain data records over the investigated period of 10 years, data from the rain gauges with fewer than 80% working days over the 10 years, e.g., approximately 2900 days, were discarded.

By this operation, the original rain gauge database, composed of approximately 4500 stations, was reduced to a database of 2041 stations with consistent data series. The network of selected rain gauges exhibits a good spatial distribution in all WHZs except for four, i.e., Sici-G in the south-eastern Sicilia Region, Tosc-I on Elba Island, Tosc-E3 on the southern coast of the Tuscany Region and Camp-8 on the southern coast of the Campania Region. In these areas, there were no instruments that satisfied the minimum requirements.

### Statistical rain data analysis

The refined database was analysed to extract relevant information about the spatio-temporal distribution of rain over Italy. For these purposes, the daily rainfall data were categorized into five classes according to the ranks used for the daily meteorological warning bulletin emitted by the weather CFC workgroup of the Italian DPC (Table 1).

**Table 1:** WHZ rainfall classes, as defined by the weather CFC workgroup of the Italian DPC, with the addition of the “Heavy rate” class.

Low rate	5–20 mm/day
Medium rate	21–60 mm/day
High rate	61–100 mm/day
Very high rate	101–150 mm/day
Heavy rain	> 151 mm/day

It is worth noting that rainfall values below 5 mm/day were not considered in this study. This choice was made to avoid the influence of potential errors caused by hourly data sums affected by systematic instrument errors and because very low values are not relevant for civil protection or meteorological warning purposes. Then, the number of events recorded within each class was automatically assessed and processed to extract the frequency of occurrence of events annually for each WHZ.

### Rainfall spatial distribution and frequency analysis

The frequency distribution of rain in the above classes for each WHZ were assessed for the entire 2010–2019 period, both yearly and averaging over the 10 years of study. The percentages were assessed by considering the numbers of events within each class related to the number of events recorded in the entire WHZ. To evaluate the average rainfall distribution in the entire 2010–2019 period, the same approach was applied; the number of events within each class were counted and ranked in the 10 years of study related to the total number of events recorded. The annual frequency for each WHZ was plotted to investigate and analyse if variation were recognized possible from 2010 to 2019. It was difficult to identify relevant variation in frequency distribution for the investigated period, but interesting evolutions were highlighted in some WHZs.

## Supplementary Information

Supplementary Information 1.

Supplementary Information 2.

Supplementary Information 3.

## Data Availability

The data that support the findings of this study are available from the corresponding author upon reasonable request.
