# Supplementary material for: Ten years of pluviometric analyses in Italy for civil protection purposes
Source: Sci Rep. 2021 Oct 13;11:20302. doi: 10.1038/s41598-021-99874-w (PMC8514502; doi:10.1038/s41598-021-99874-w)
Supplement: Supplementary file 1 — Supplementary Information 1. [file 41598_2021_99874_MOESM1_ESM.pdf]

# Supplementary material

## Suppl. 1

## Frequency of rainfall classes

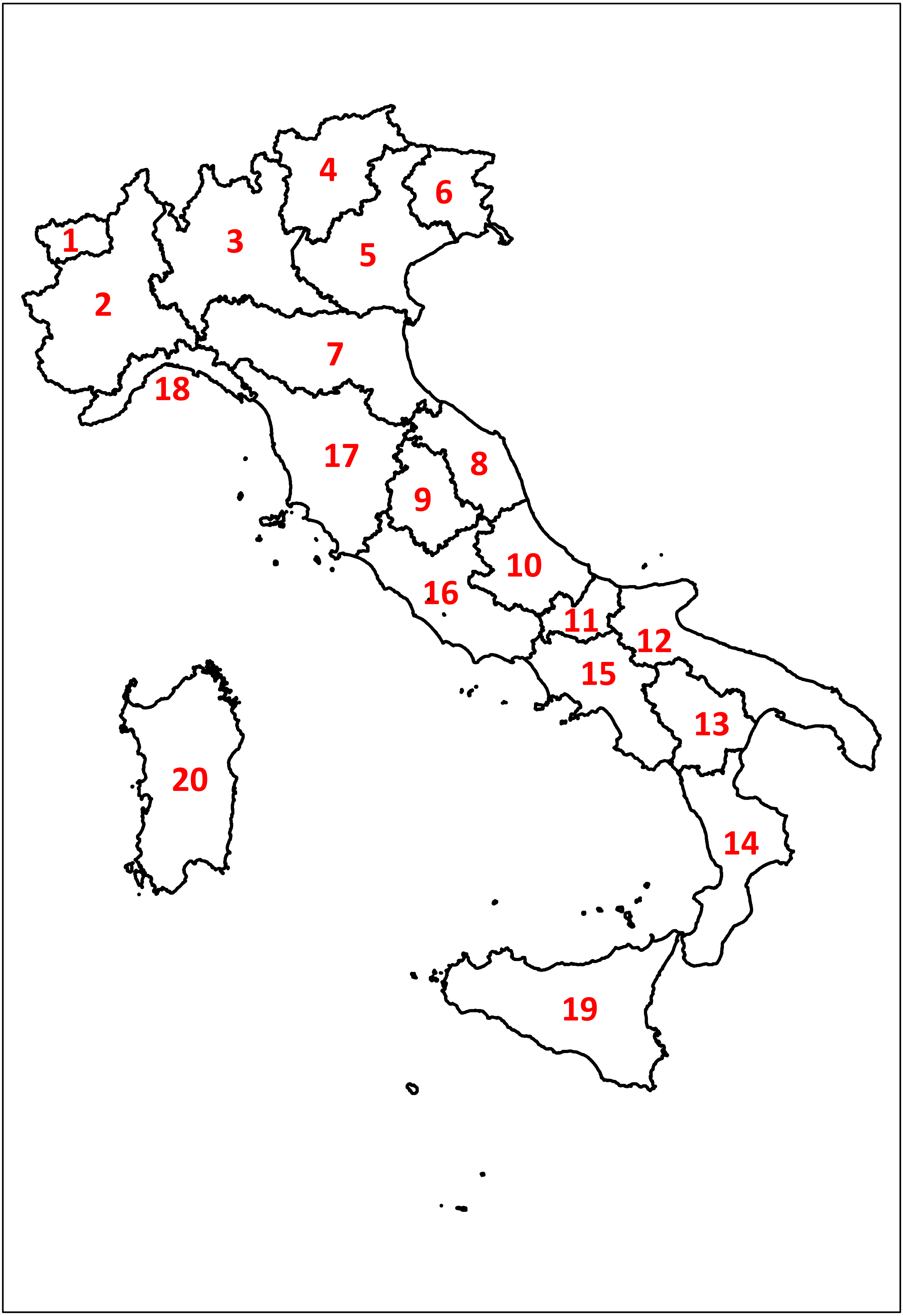
1
2
3
4
5
6
7
8
9
10
11
12
13
14
15
16
17
18
19
20

# MM π Abruzzo Region

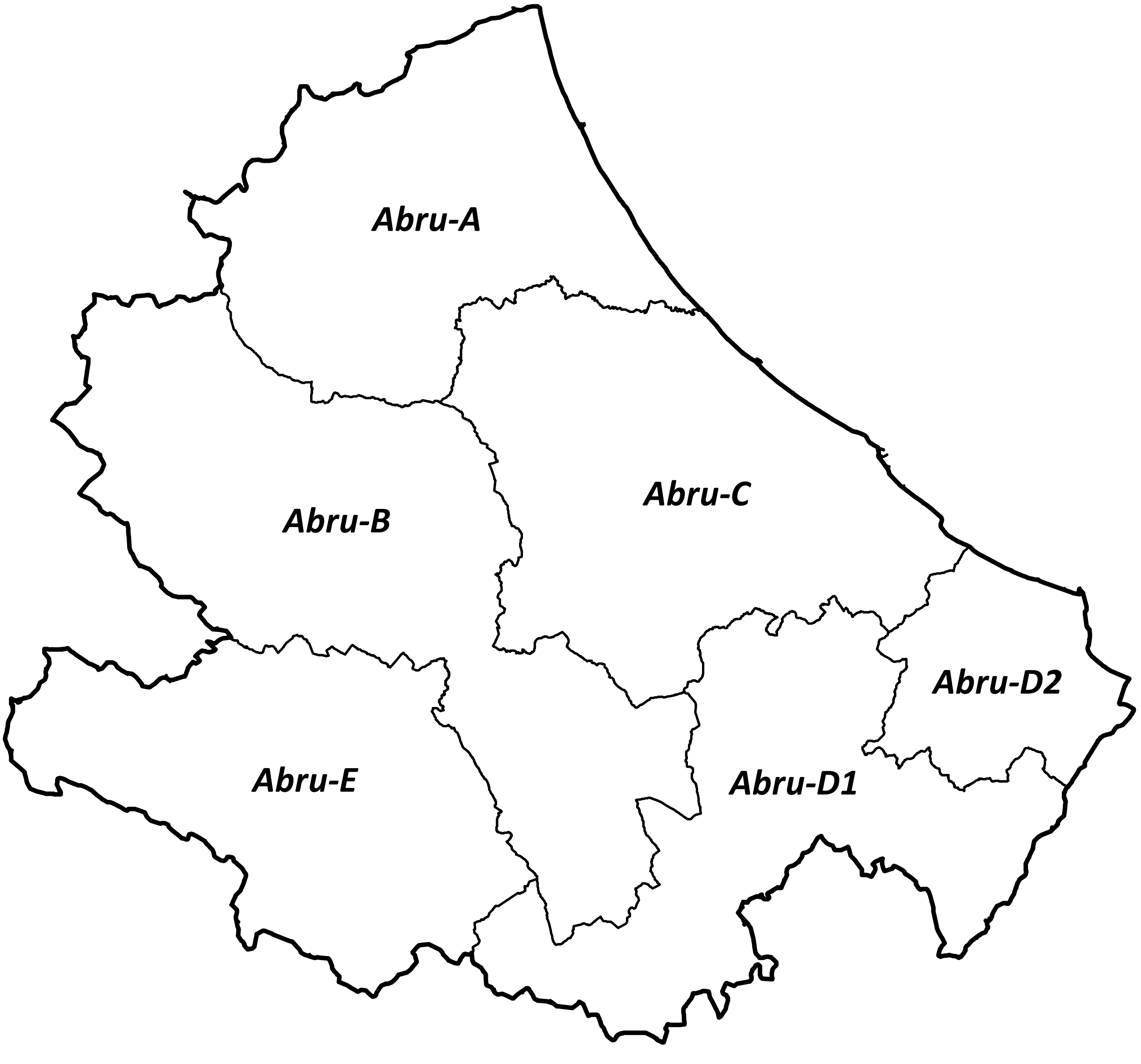

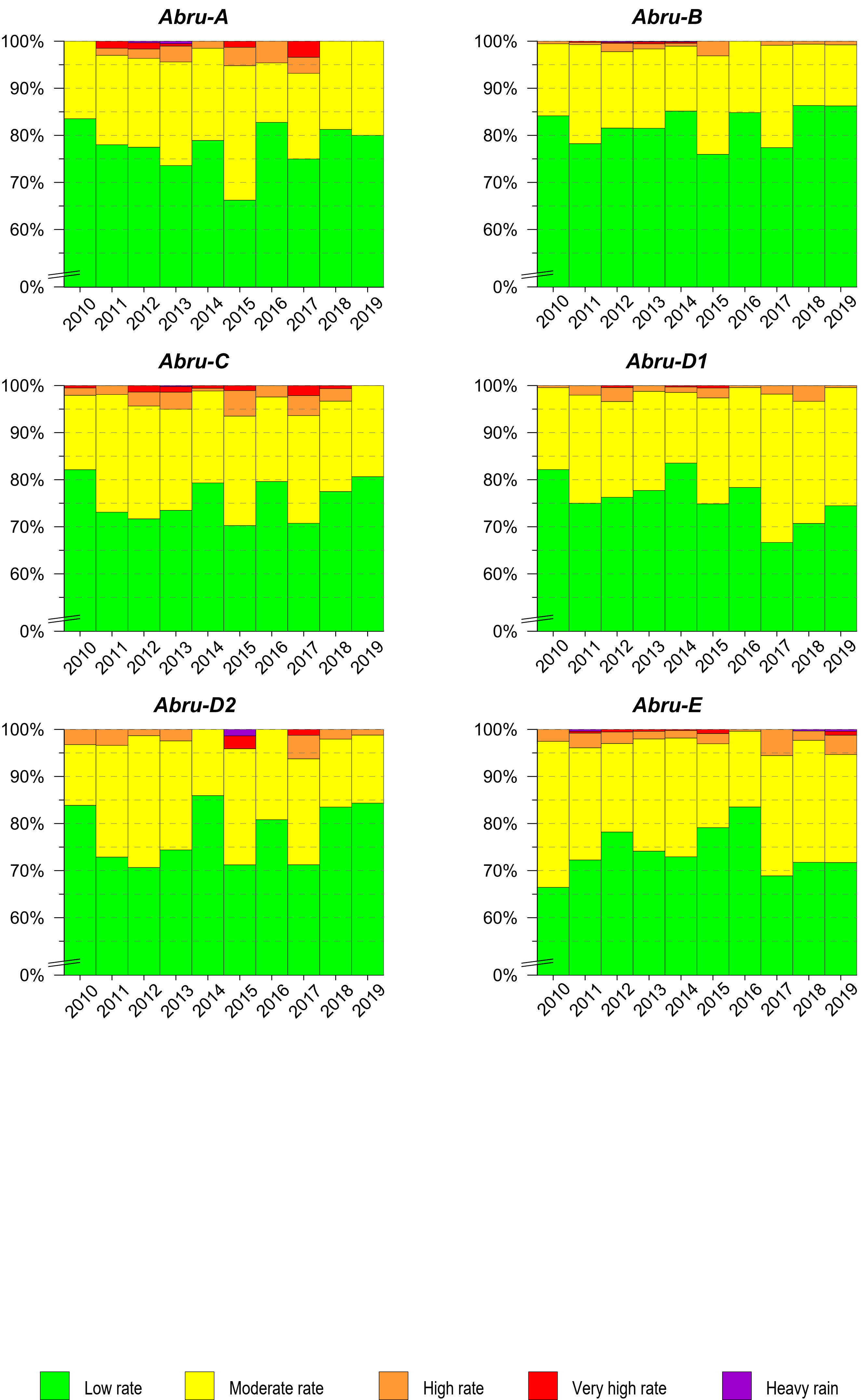

Abru-A
Abru-B
Abru-C
Abru-D1
Abru-D2
Abru-E
100%
90%
80%
70%
60%
0%
2010
2011
2012
2013
2014
2015
2016
2017
2018
2019
Low rate
Moderate rate
High rate
Very high rate
Heavy rain

# 13 - Basilicata Region

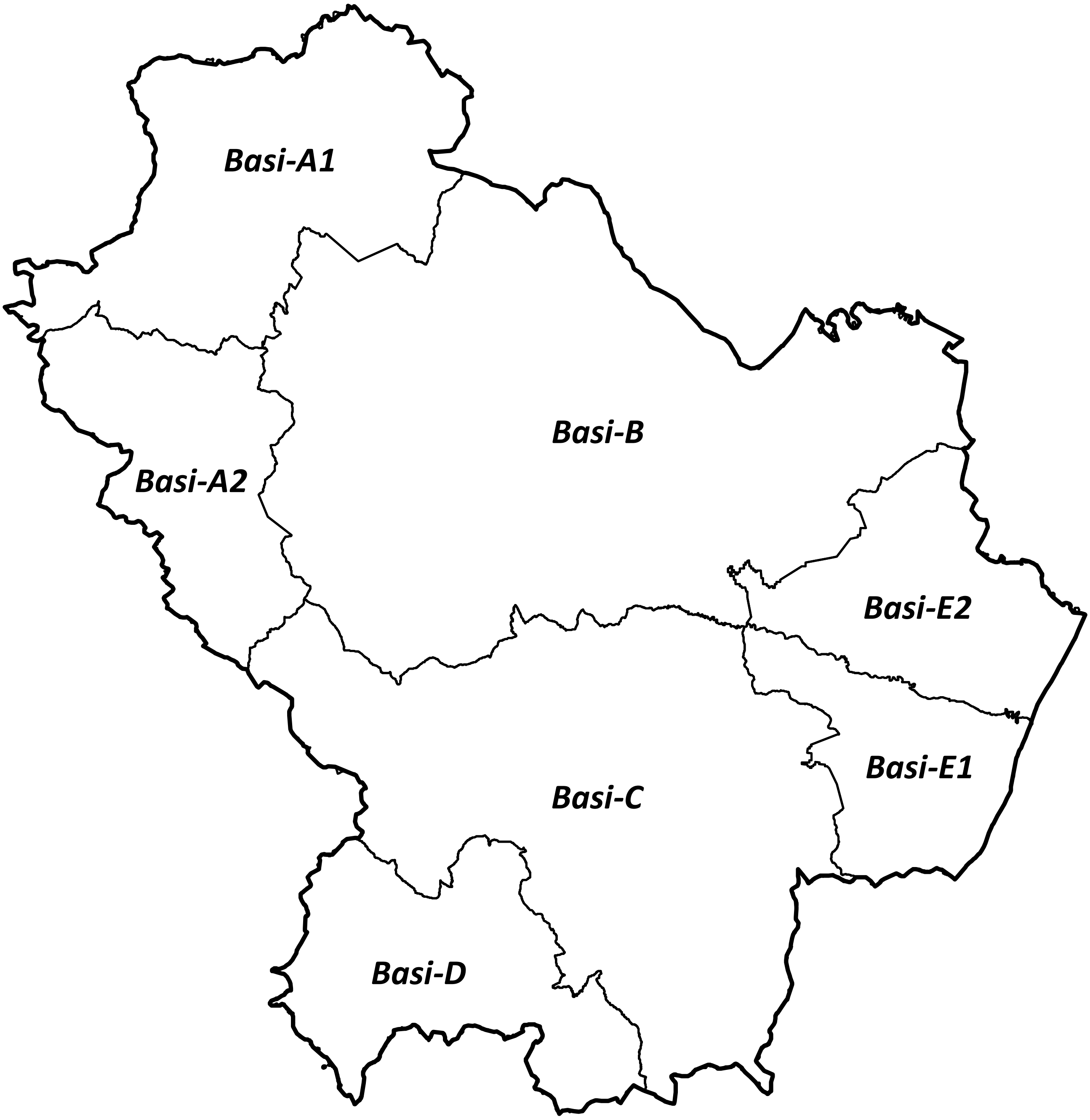

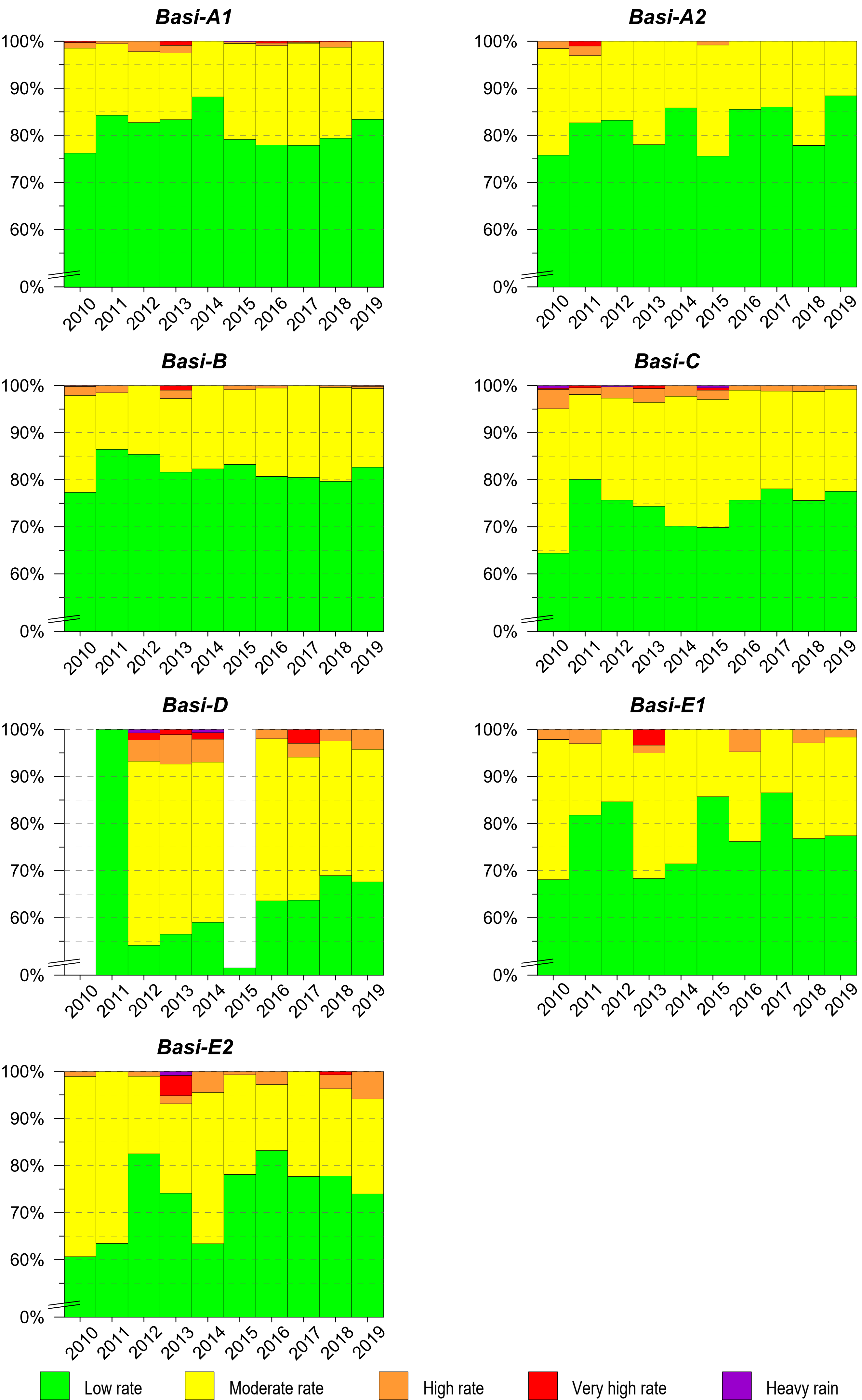
Basi-A1
Basi-A2
Basi-B
Basi-C
Basi-D
Basi-E1
Basi-E2
100%
90%
80%
70%
60%
0%
2010
2011
2012
2013
2014
2015
2016
2017
2018
2019
Low rate
Moderate rate
High rate
Very high rate
Heavy rain

# 14 - Calabria Region

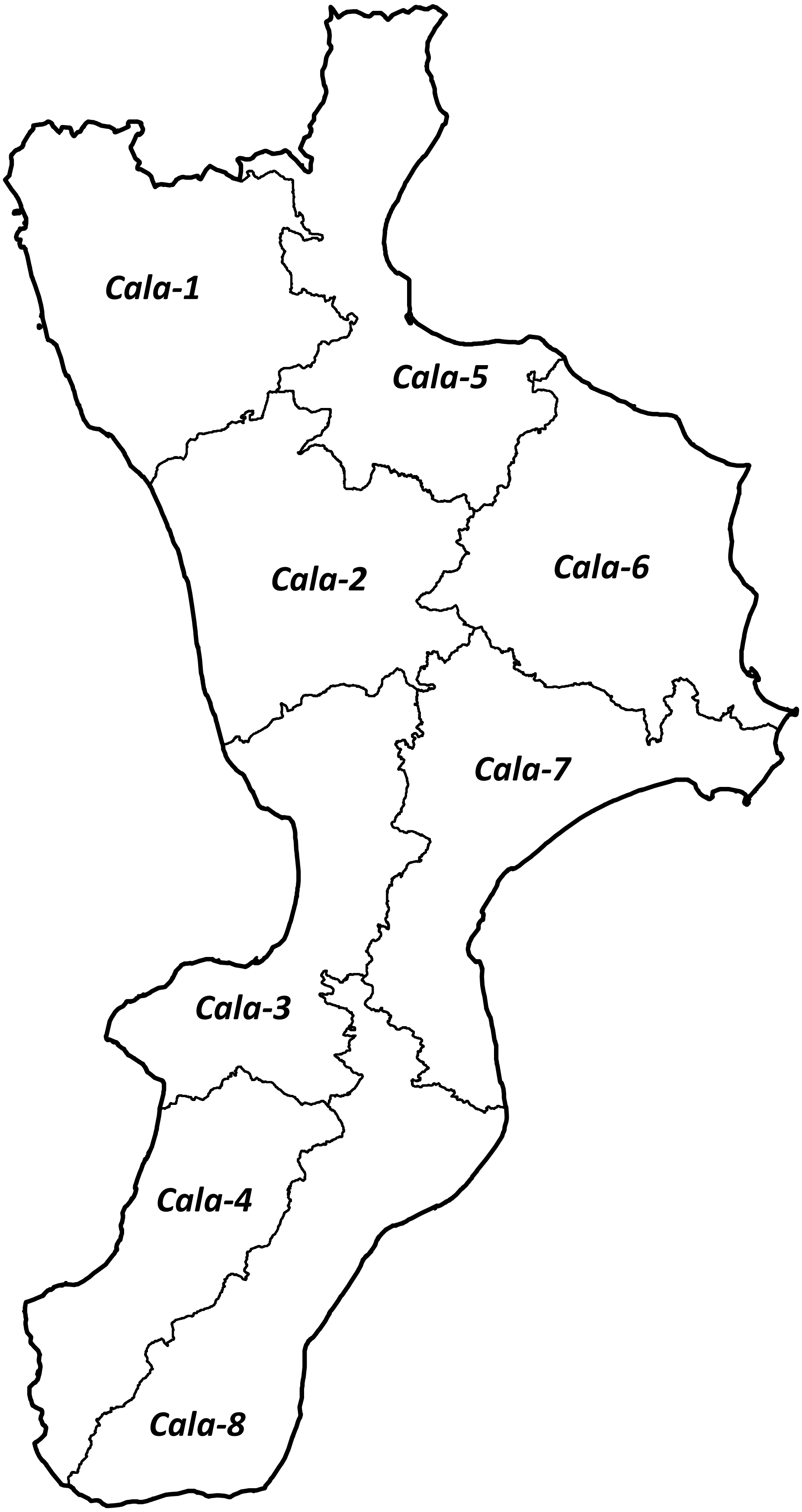

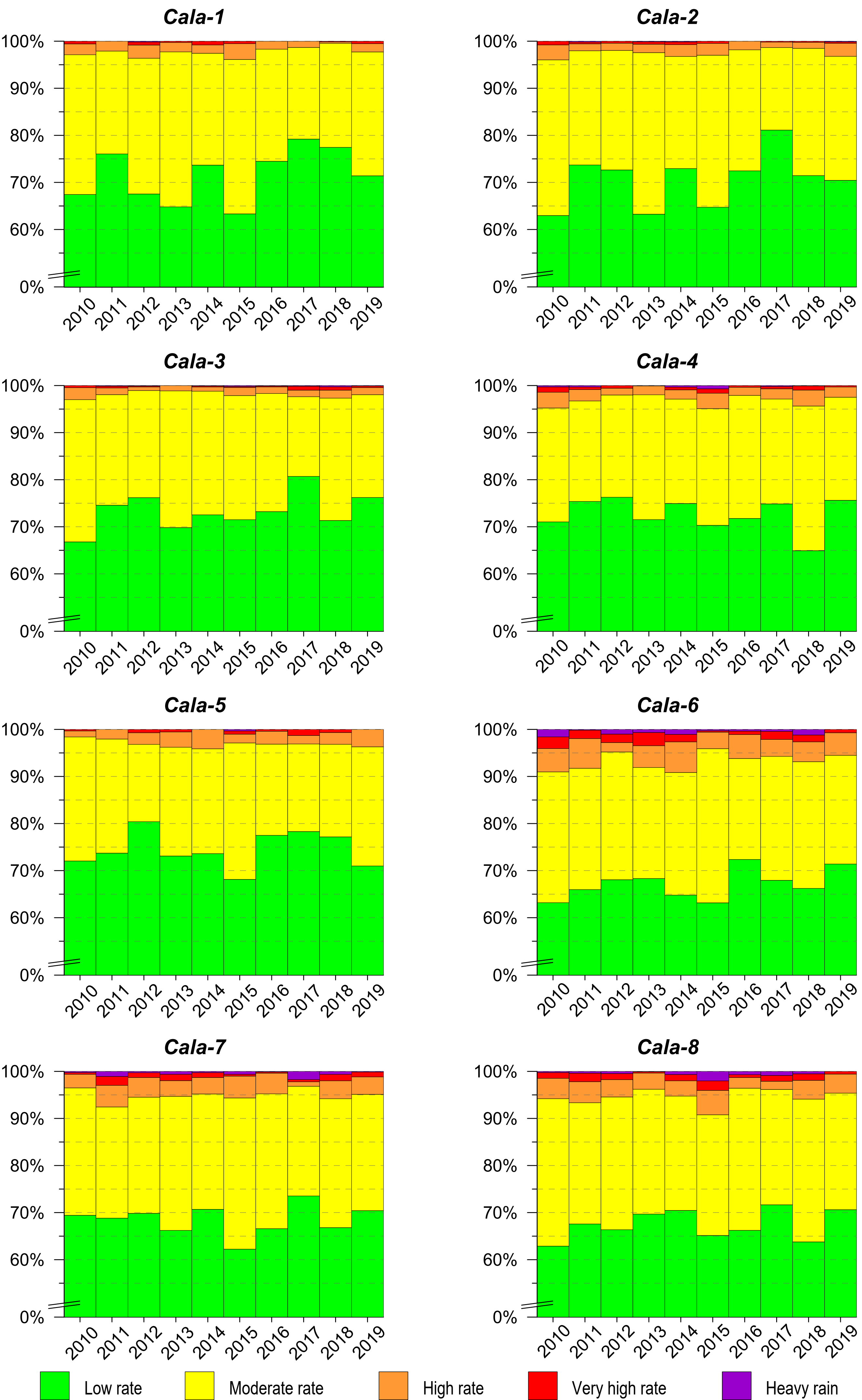

Cala-1
Cala-2
Cala-3
Cala-4
Cala-5
Cala-6
Cala-7
Cala-8
100%
90%
80%
70%
60%
0%
2010
2011
2012
2013
2014
2015
2016
2017
2018
2019
Low rate
Moderate rate
High rate
Very high rate
Heavy rain

# 15 - Campania Region

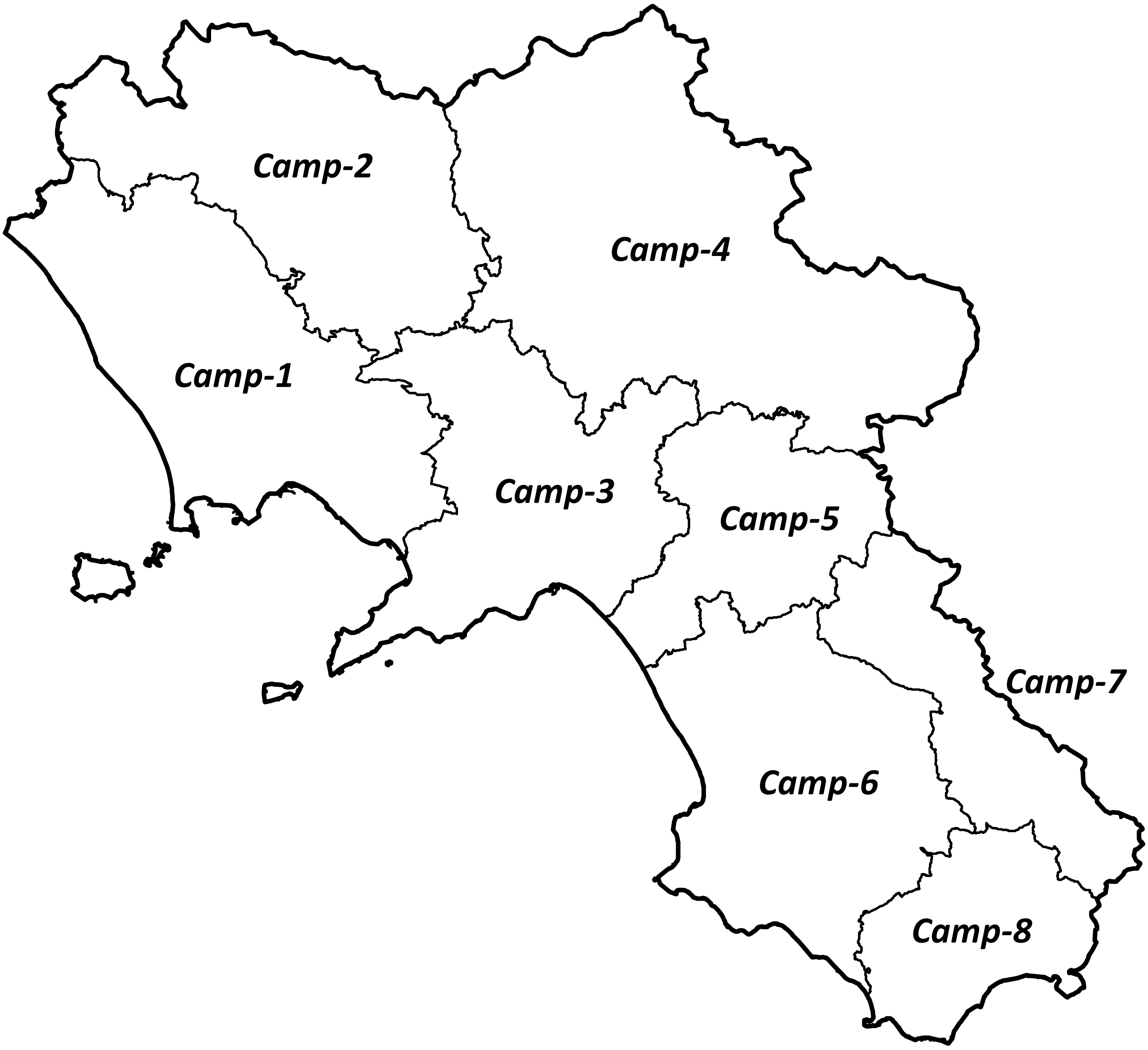

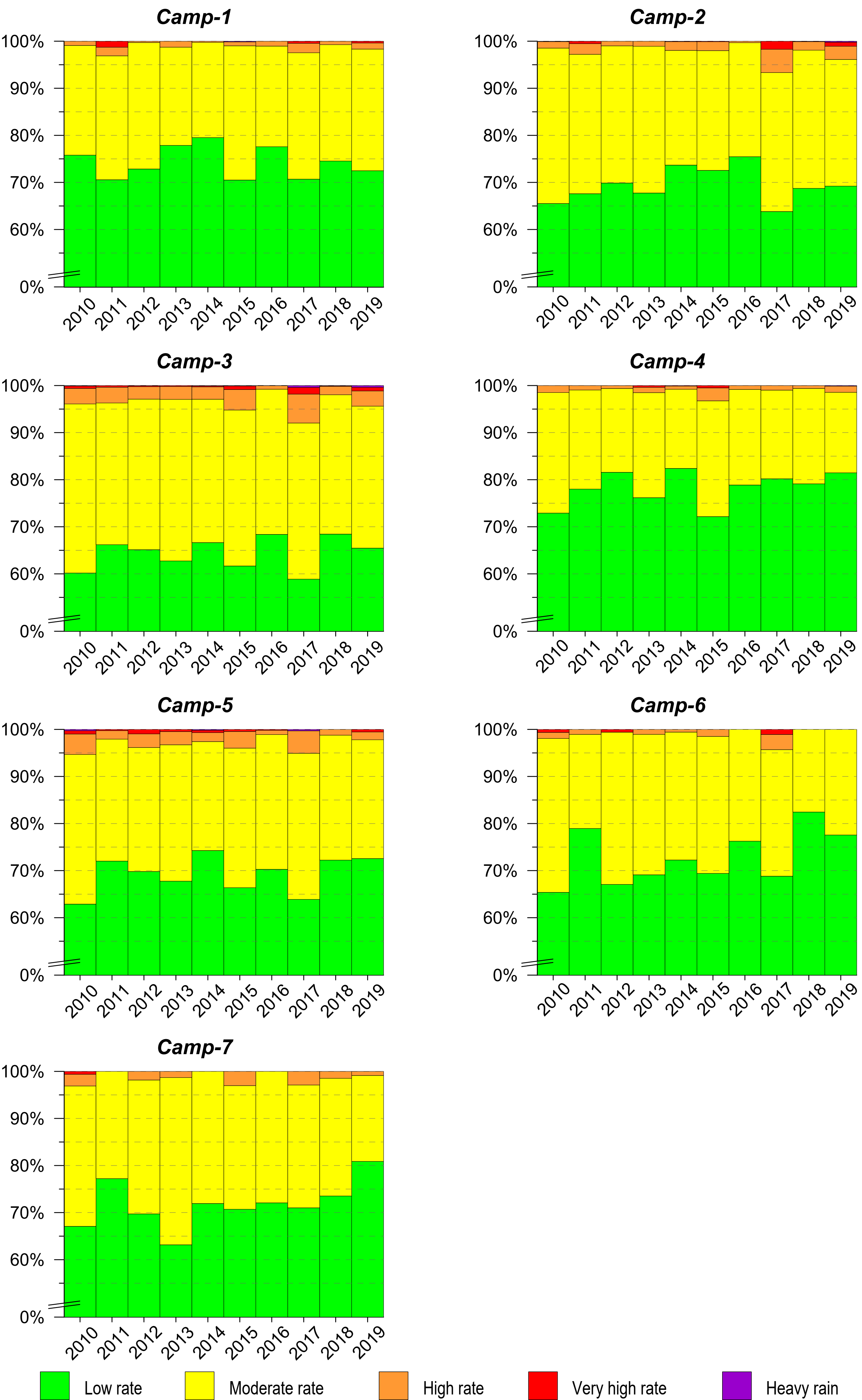

Camp-1
Camp-2
Camp-3
Camp-4
Camp-5
Camp-6
Camp-7
100%
90%
80%
70%
60%
0%
2010
2011
2012
2013
2014
2015
2016
2017
2018
2019
Low rate
Moderate rate
High rate
Very high rate
Heavy rain

# 7 - Emilia Romagna Region

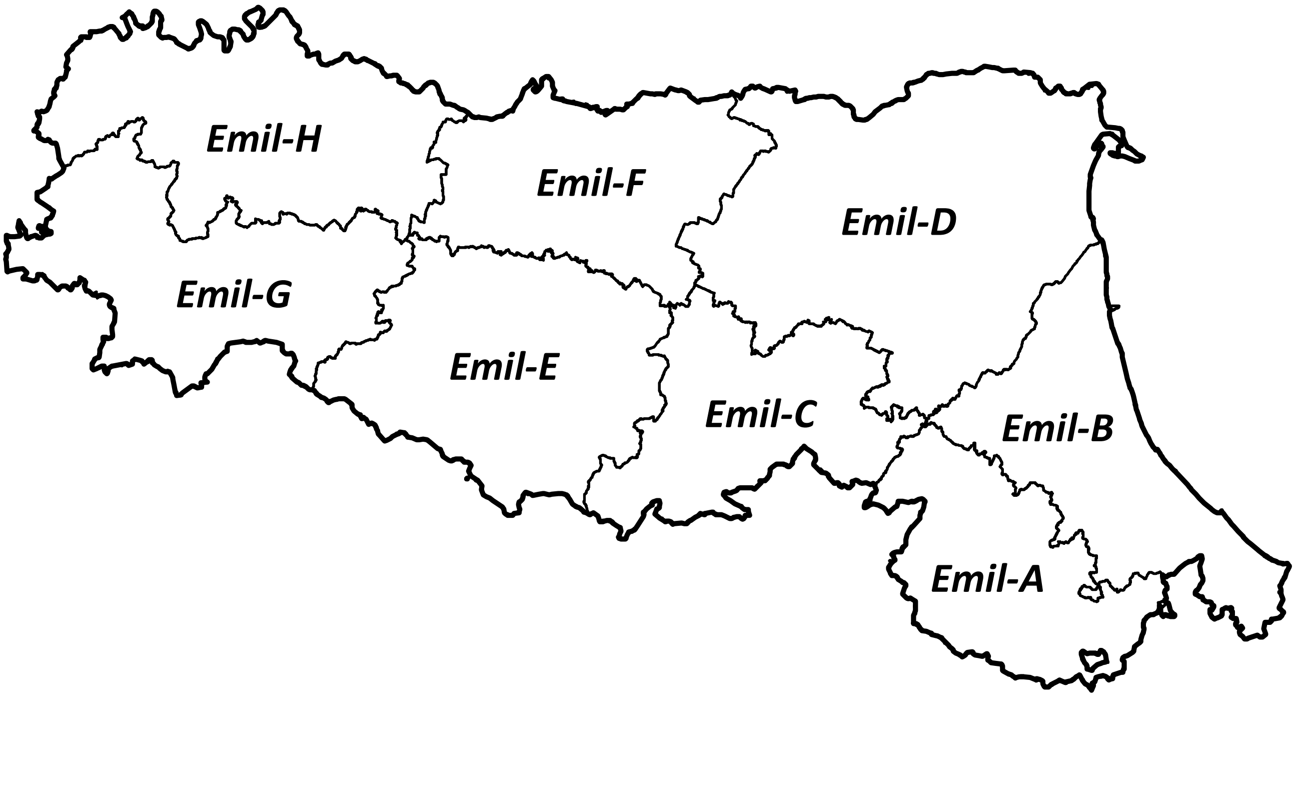

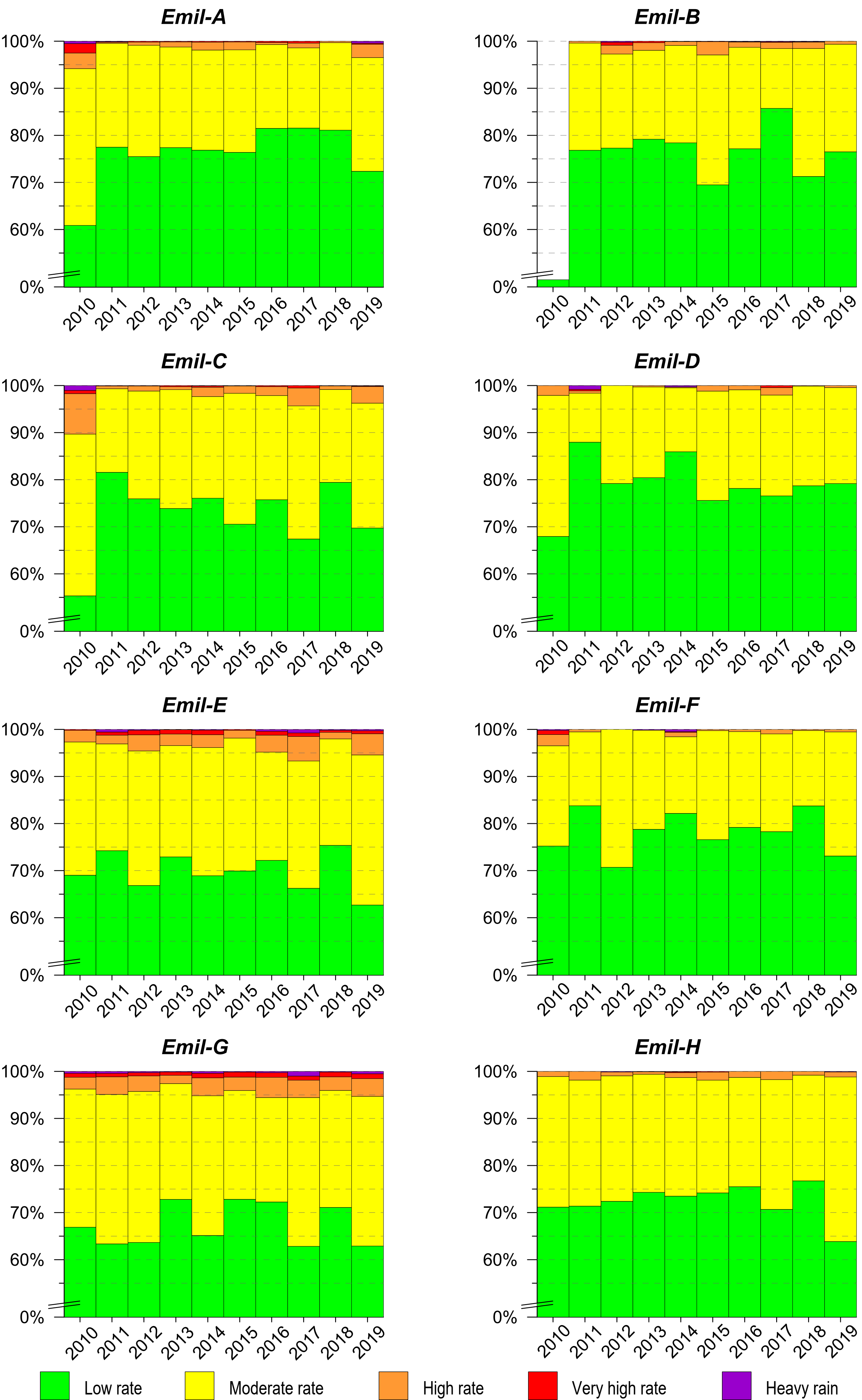
Emil-A
Emil-B
Emil-C
Emil-D
Emil-E
Emil-F
Emil-G
Emil-H
100%
90%
80%
70%
60%
0%
2010
2011
2012
2013
2014
2015
2016
2017
2018
2019
Low rate
Moderate rate
High rate
Very high rate
Heavy rain

# 6 - Friuli-Venezia Giulia Region

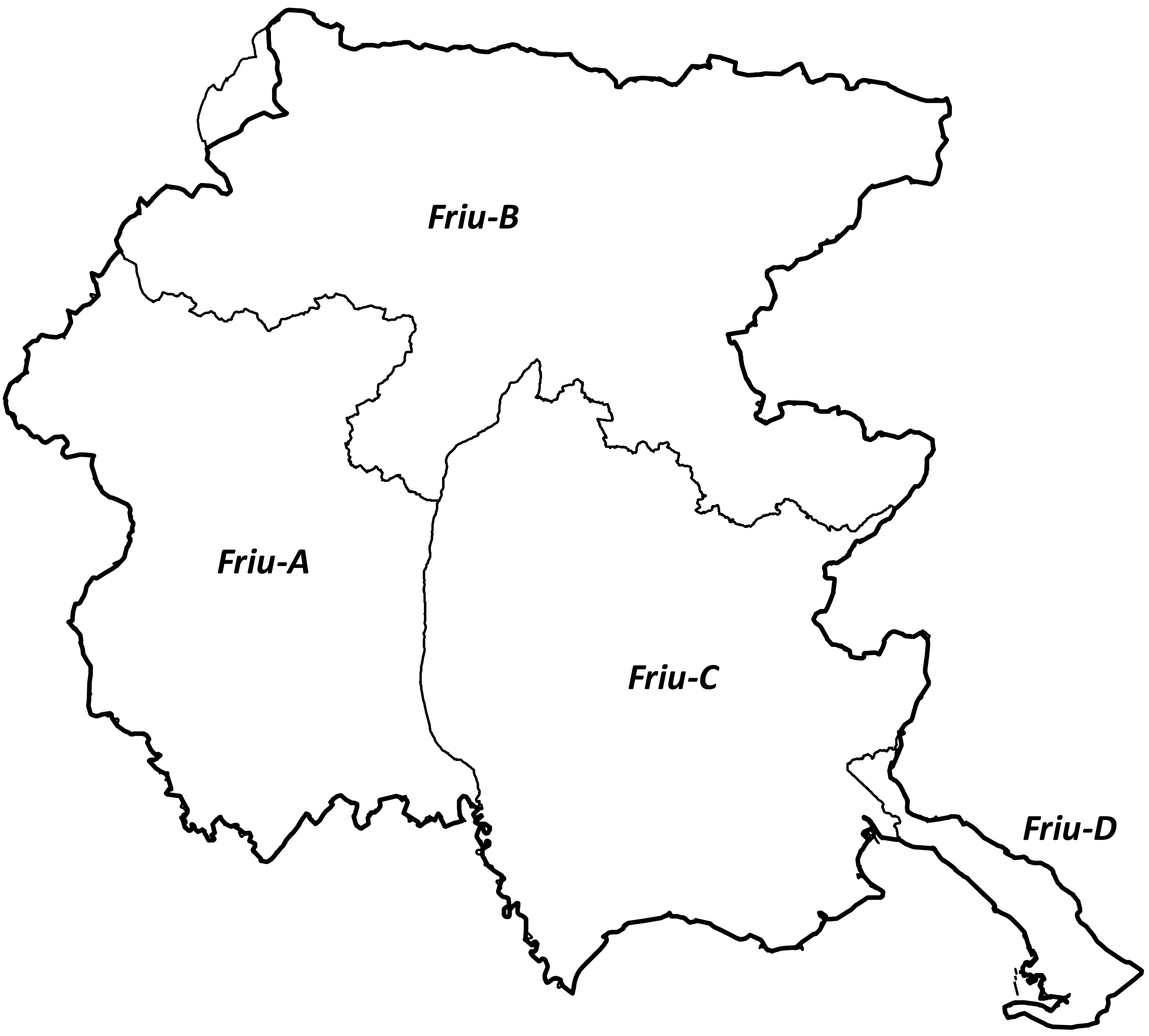

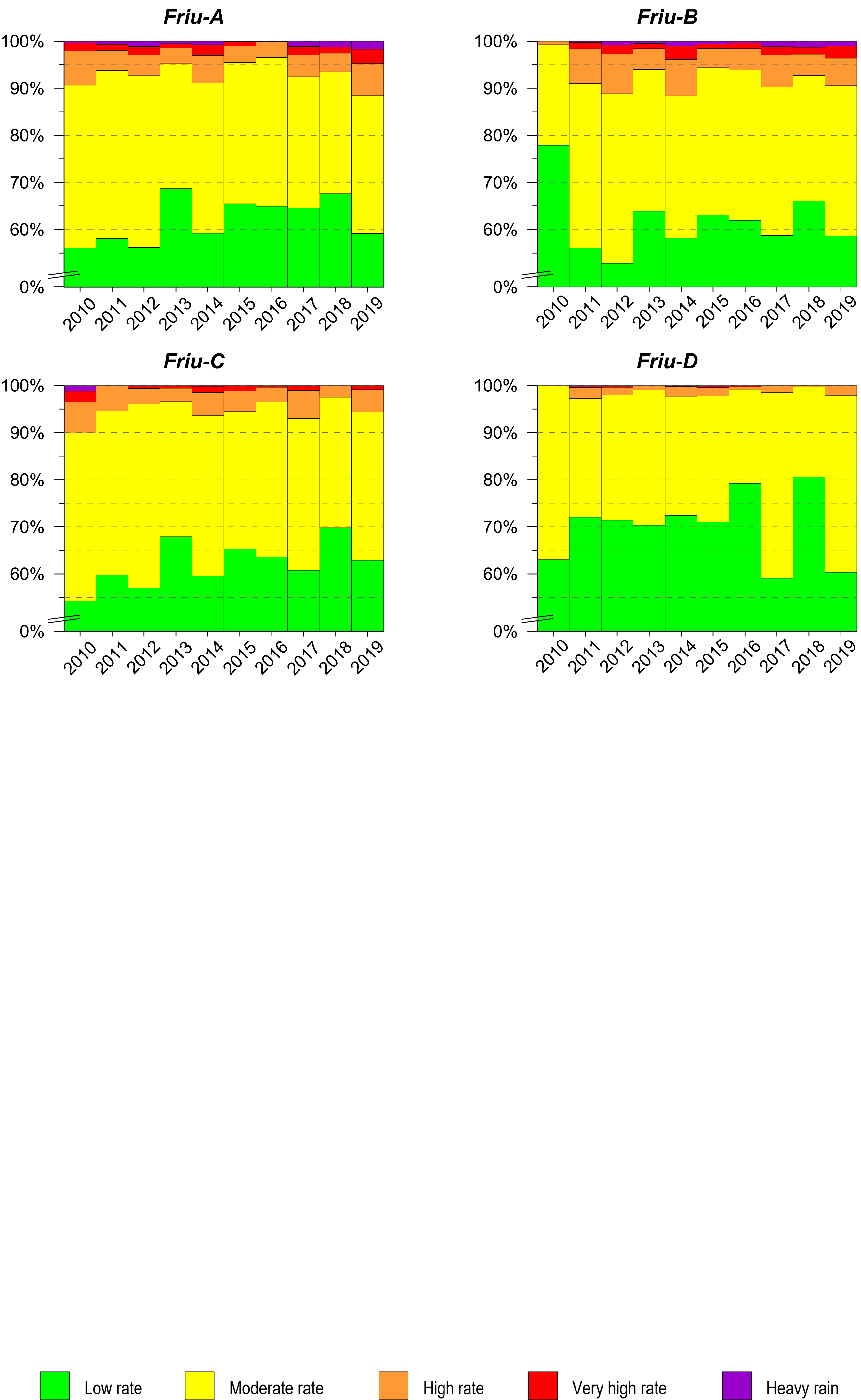
Friu-A
Friu-B
Friu-C
Friu-D
100%
90%
80%
70%
60%
0%
2010
2011
2012
2013
2014
2015
2016
2017
2018
2019
Low rate
Moderate rate
High rate
Very high rate
Heavy rain

# 16 - Lazio Region

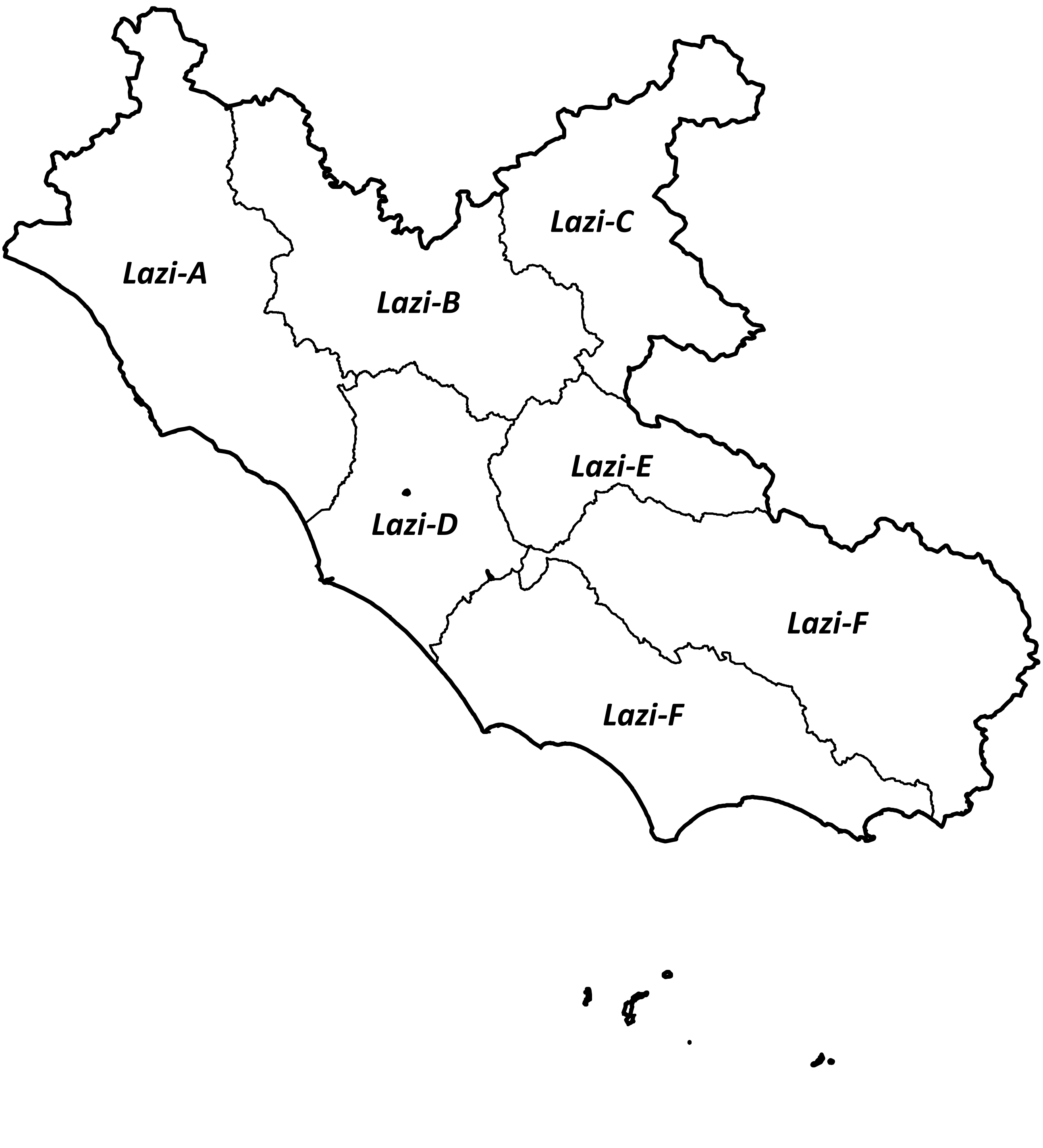

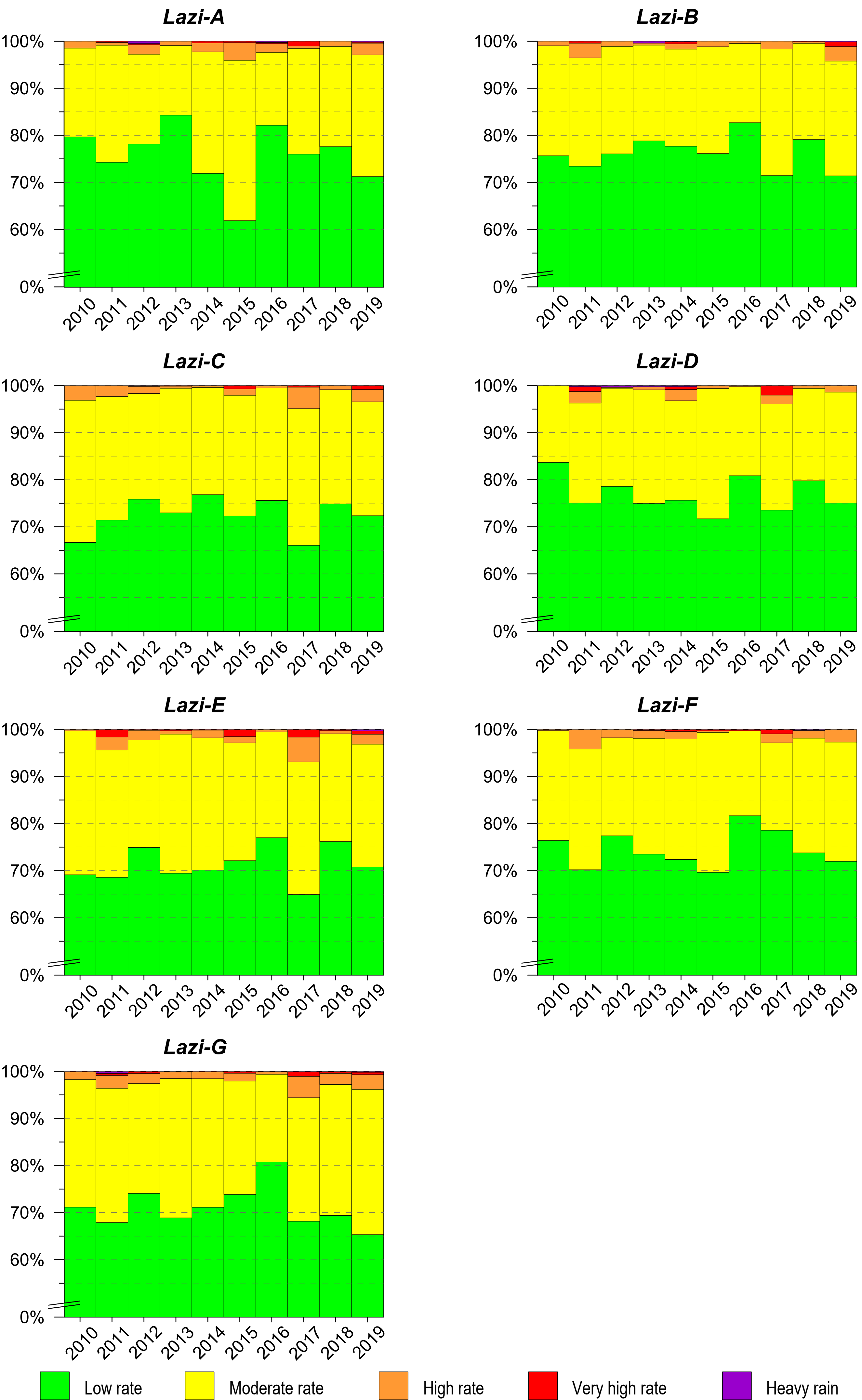
Lazi-A
Lazi-B
Lazi-C
Lazi-D
Lazi-E
Lazi-F
Lazi-G
100%
90%
80%
70%
60%
0%
2010
2011
2012
2013
2014
2015
2016
2017
2018
2019
Low rate
Moderate rate
High rate
Very high rate
Heavy rain

# 18 - Liguria Region

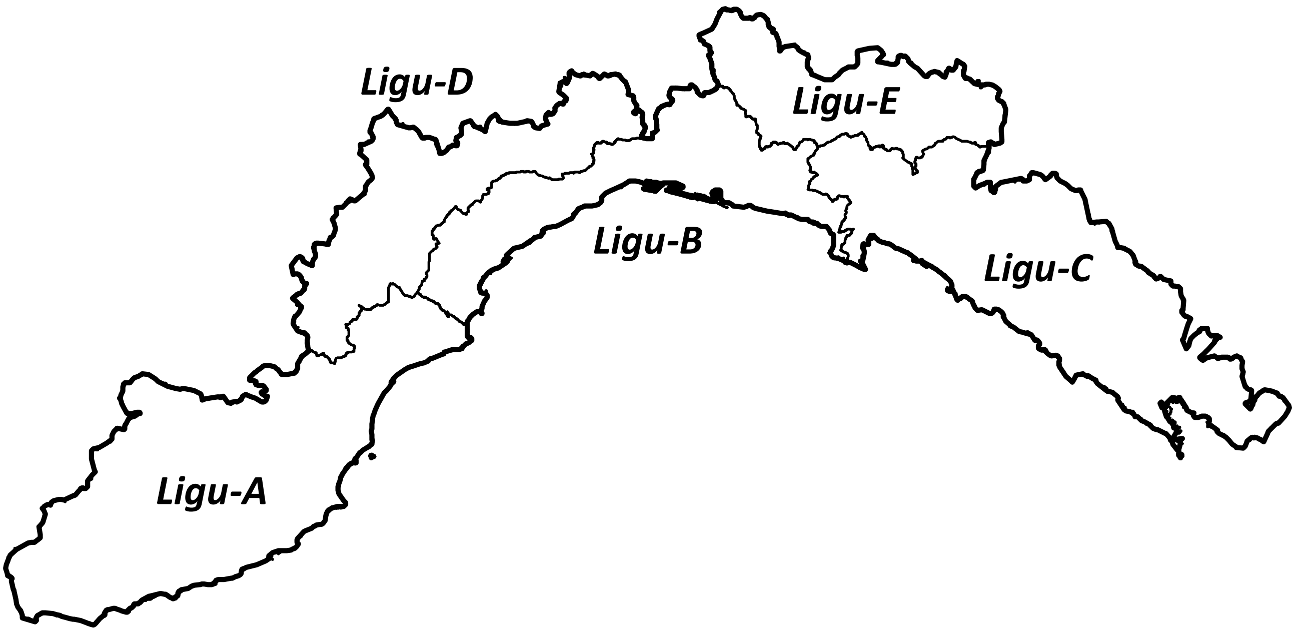

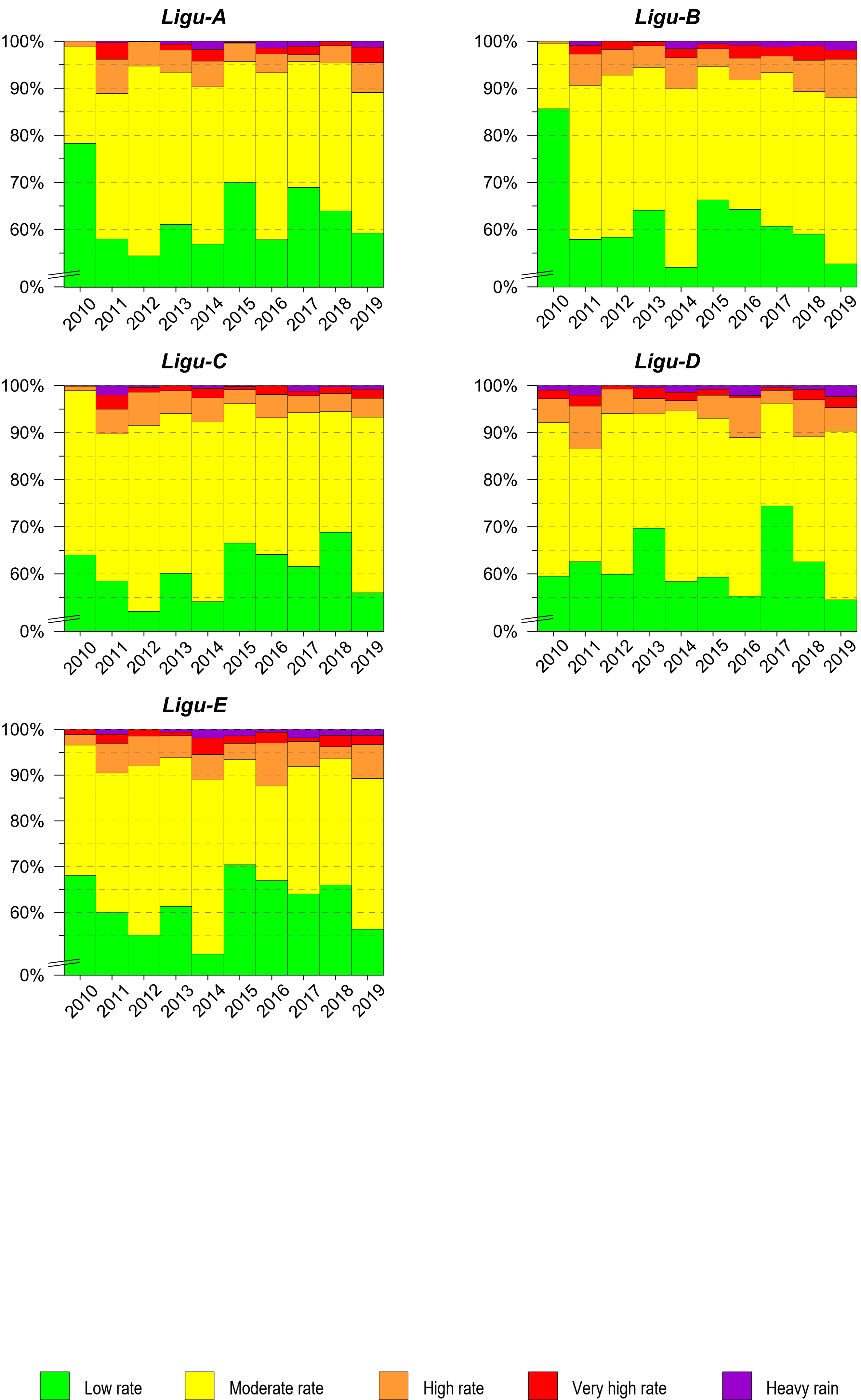

Ligu-A
Ligu-B
Ligu-C
Ligu-D
Ligu-E
100%
90%
80%
70%
60%
0%
2010
2011
2012
2013
2014
2015
2016
2017
2018
2019
Low rate
Moderate rate
High rate
Very high rate
Heavy rain

# 3 - Lombardia Region

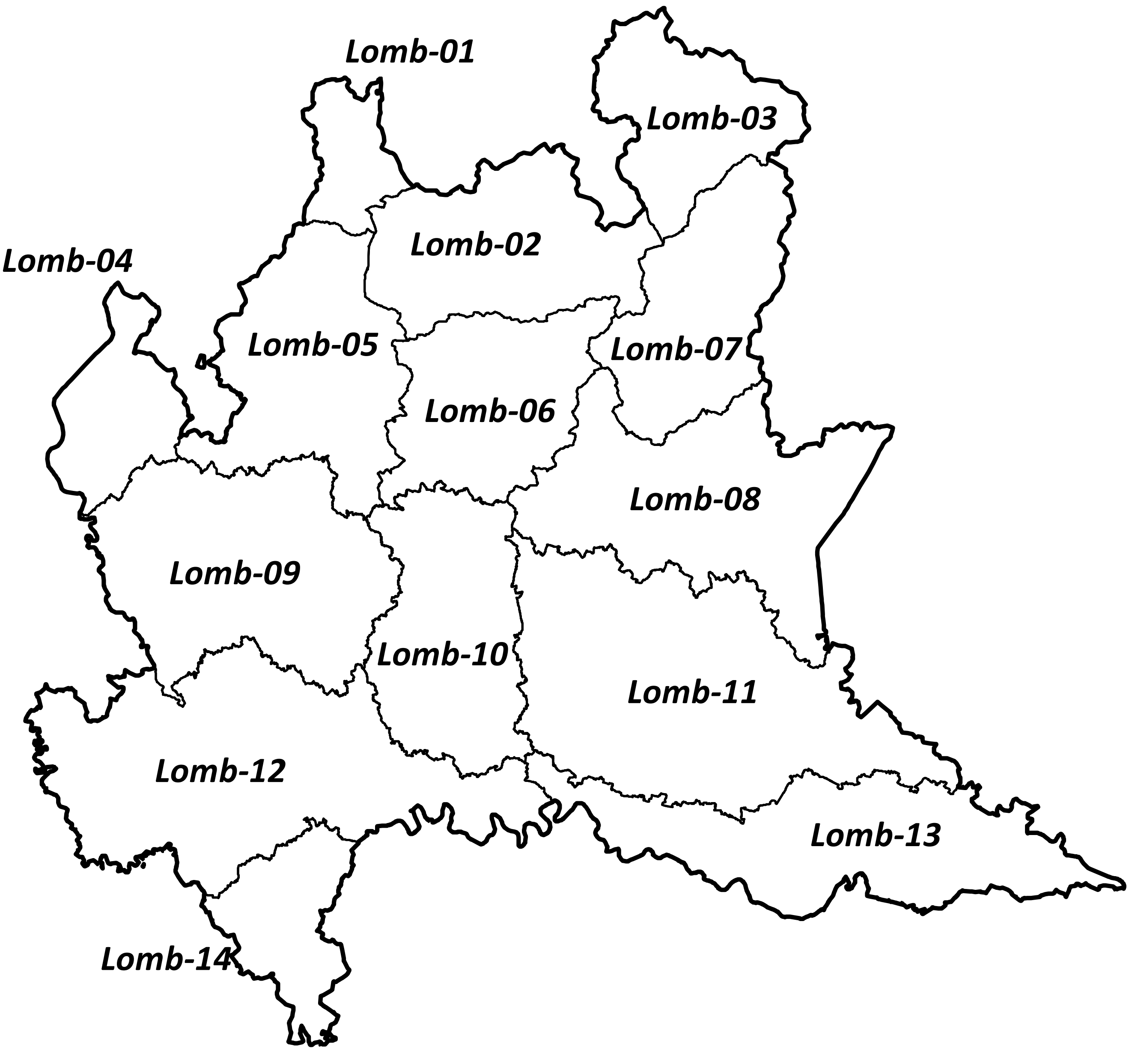

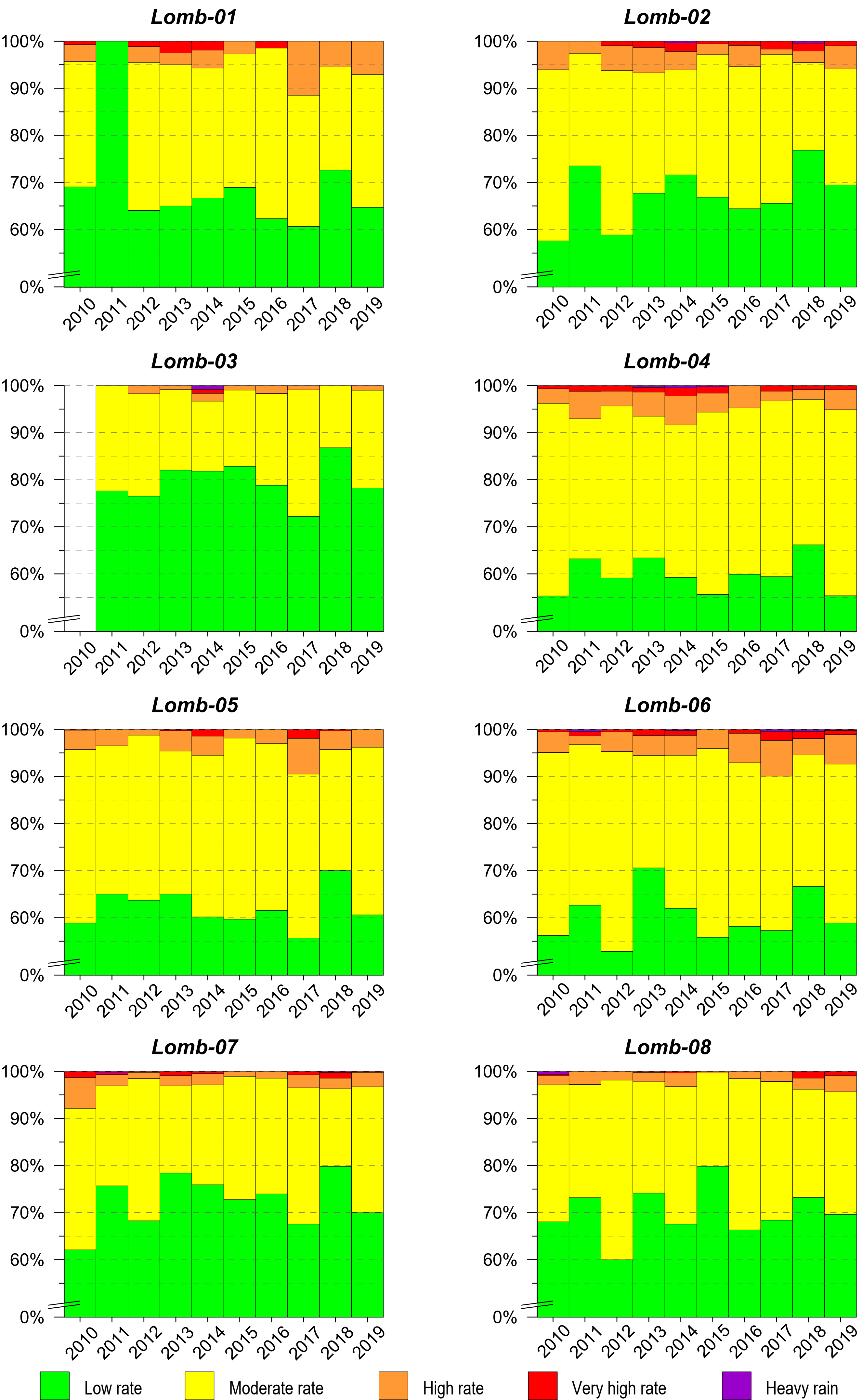

Lomb-01
Lomb-02
Lomb-03
Lomb-04
Lomb-05
Lomb-06
Lomb-07
Lomb-08
100%
90%
80%
70%
60%
0%
2010
2011
2012
2013
2014
2015
2016
2017
2018
2019
Low rate
Moderate rate
High rate
Very high rate
Heavy rain

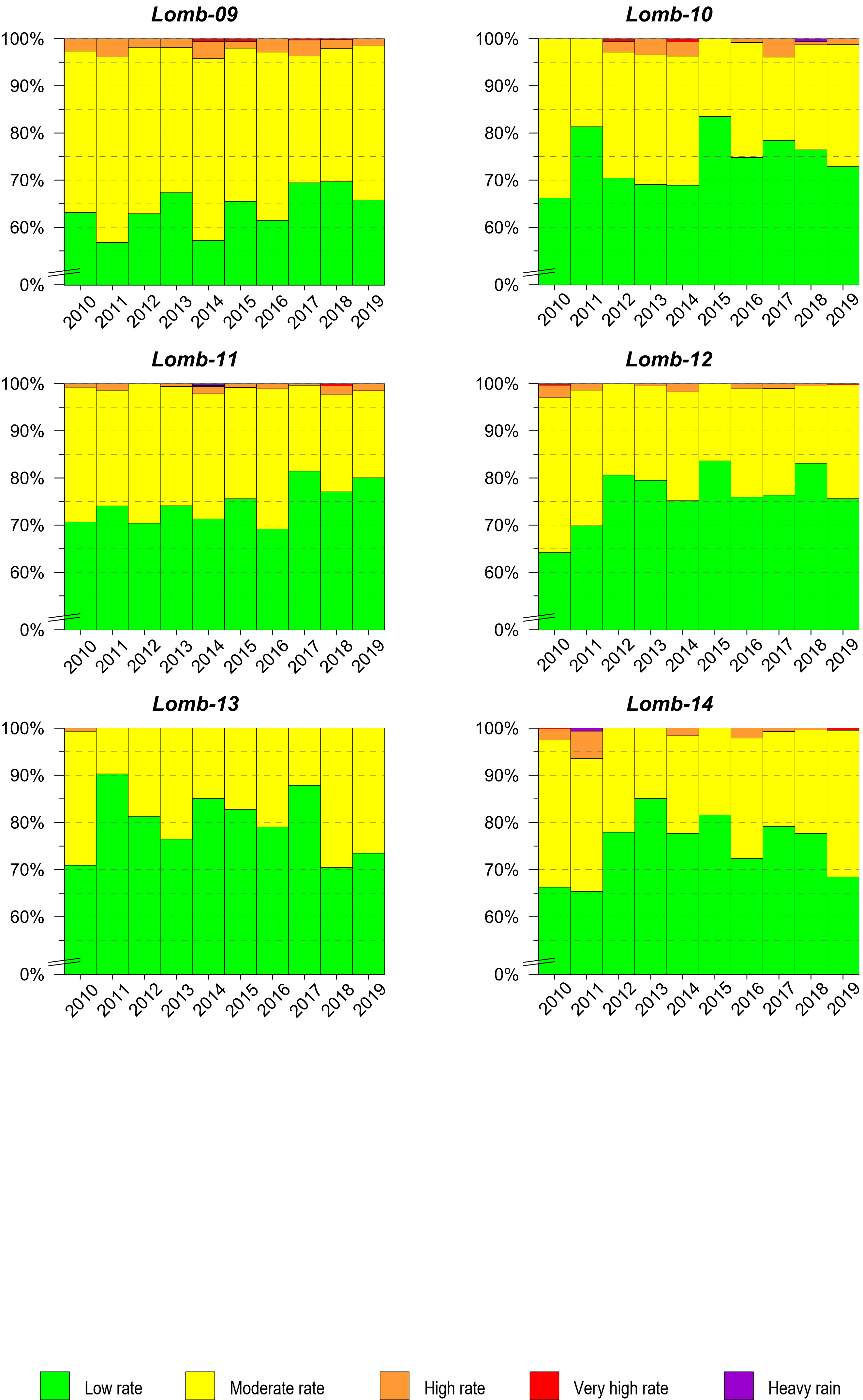
Lomb-09
Lomb-10
Lomb-11
Lomb-12
Lomb-13
Lomb-14
100%
90%
80%
70%
60%
0%
2010
2011
2012
2013
2014
2015
2016
2017
2018
2019
Low rate
Moderate rate
High rate
Very high rate
Heavy rain

# 8 - Marche Region

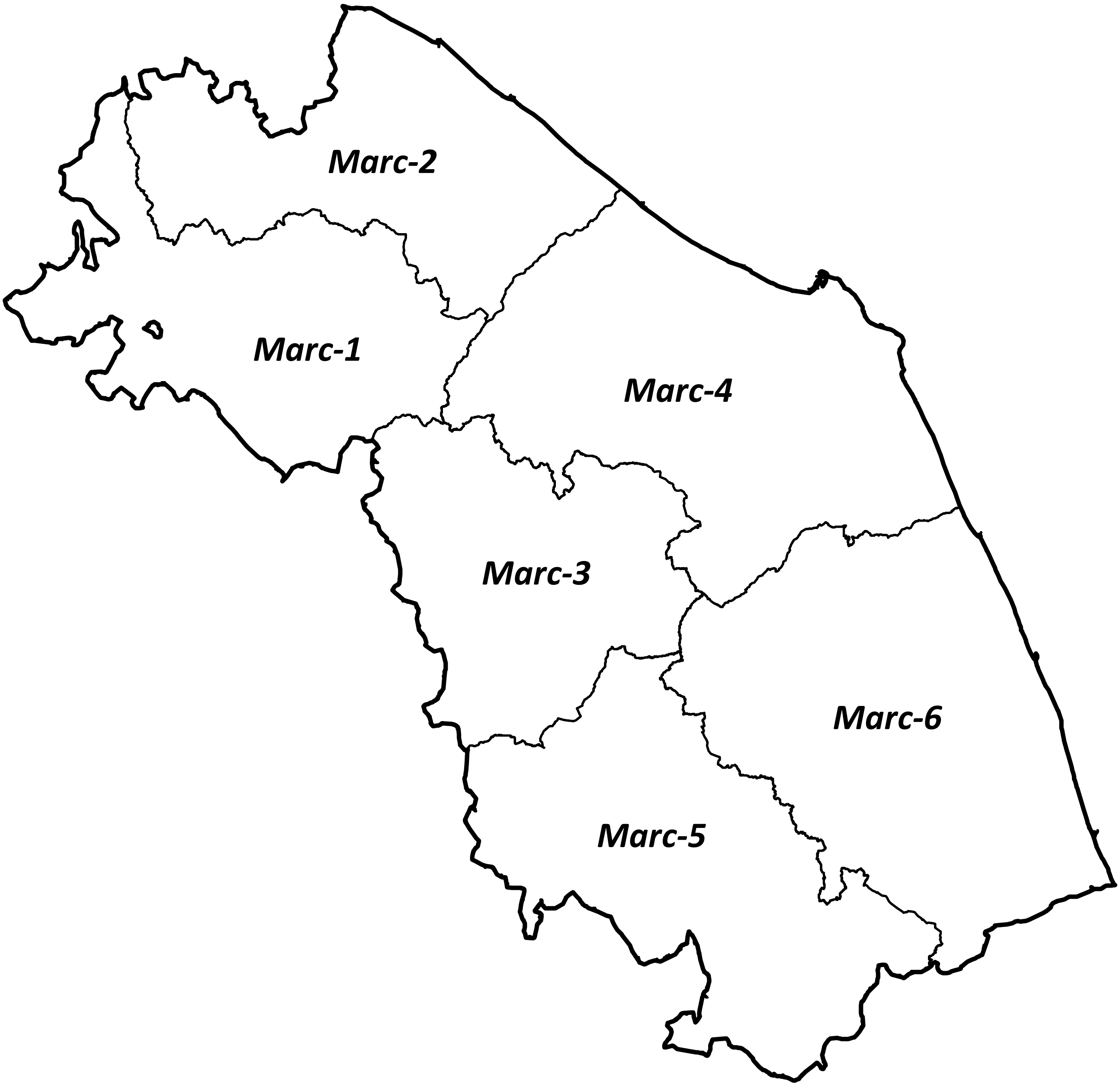

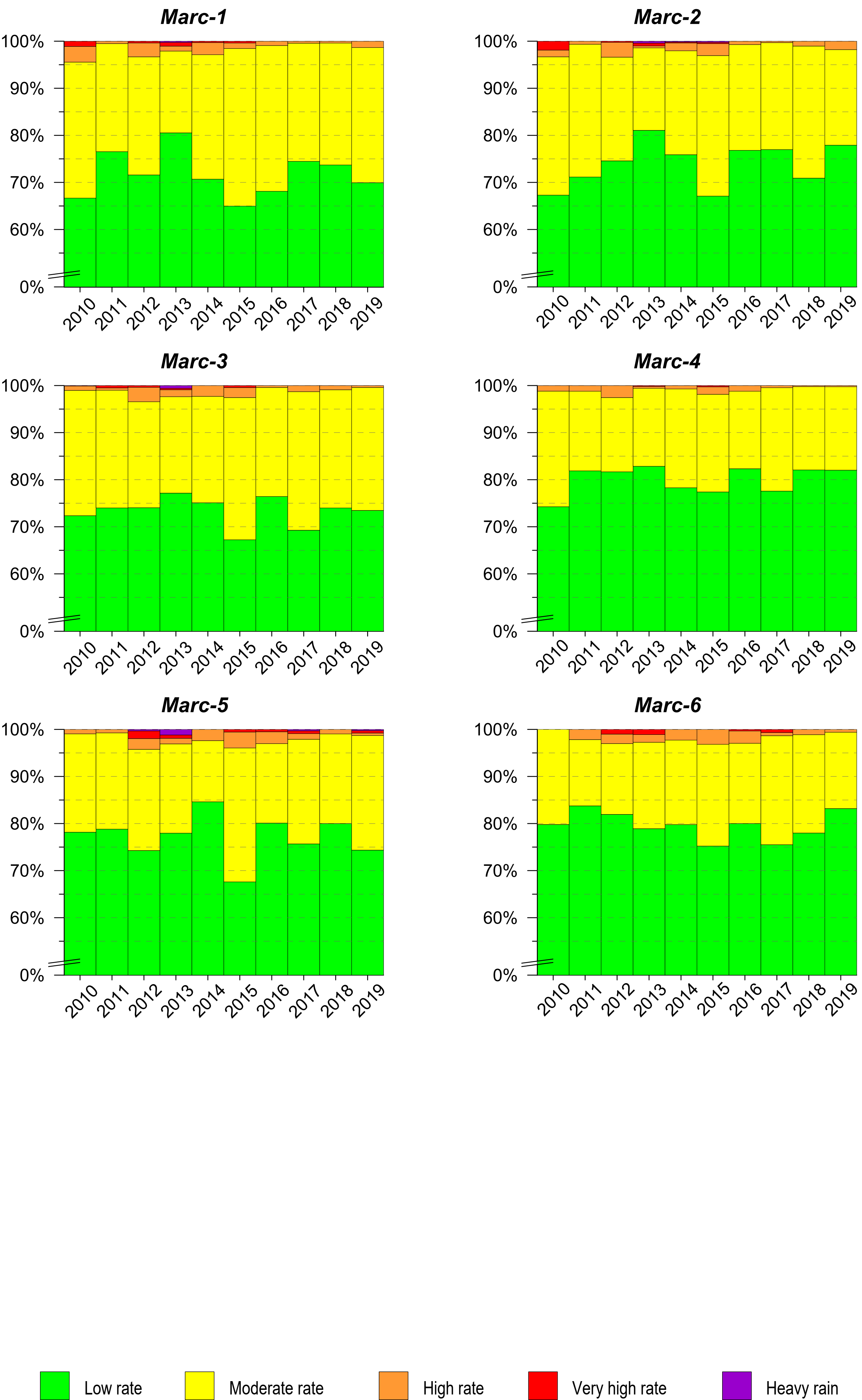

Marc-1
Marc-2
Marc-3
Marc-4
Marc-5
Marc-6
100%
90%
80%
70%
60%
0%
2010
2011
2012
2013
2014
2015
2016
2017
2018
2019
Low rate
Moderate rate
High rate
Very high rate
Heavy rain

# 11 - Molise Region

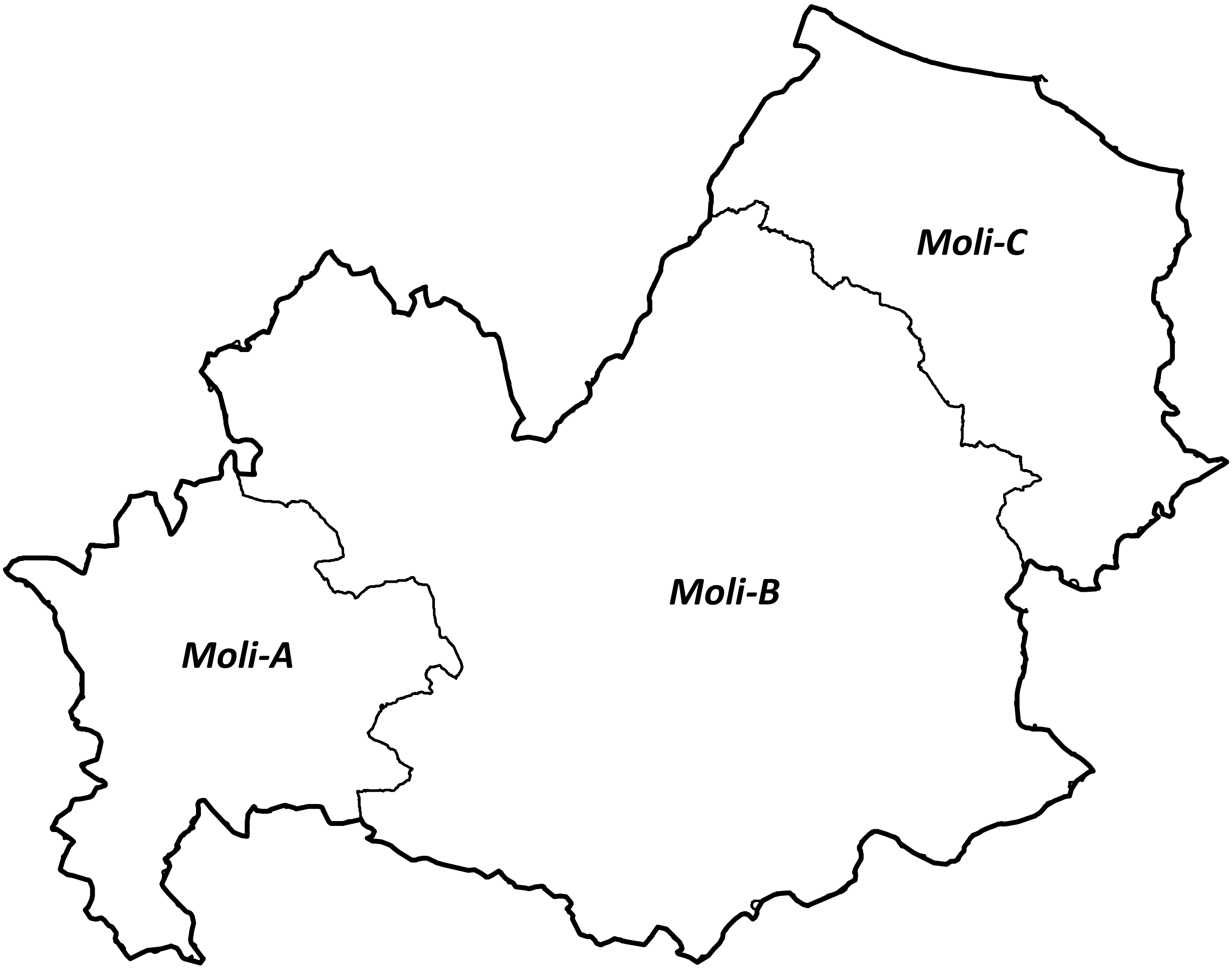

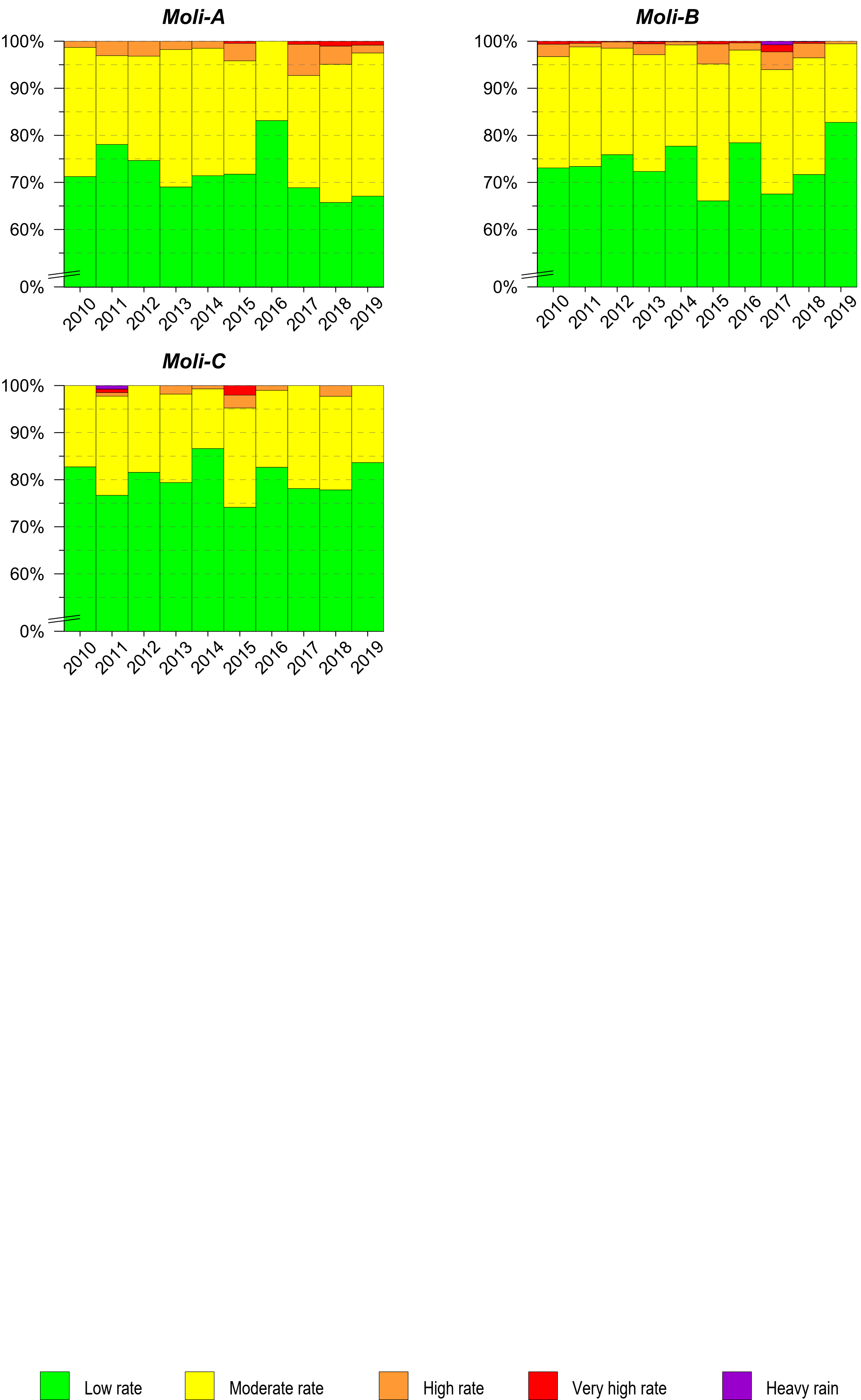
Moli-A
Moli-B
Moli-C
100%
90%
80%
70%
60%
0%
2010
2011
2012
2013
2014
2015
2016
2017
2018
2019
Low rate
Moderate rate
High rate
Very high rate
Heavy rain

# 2 - Piemonte Region

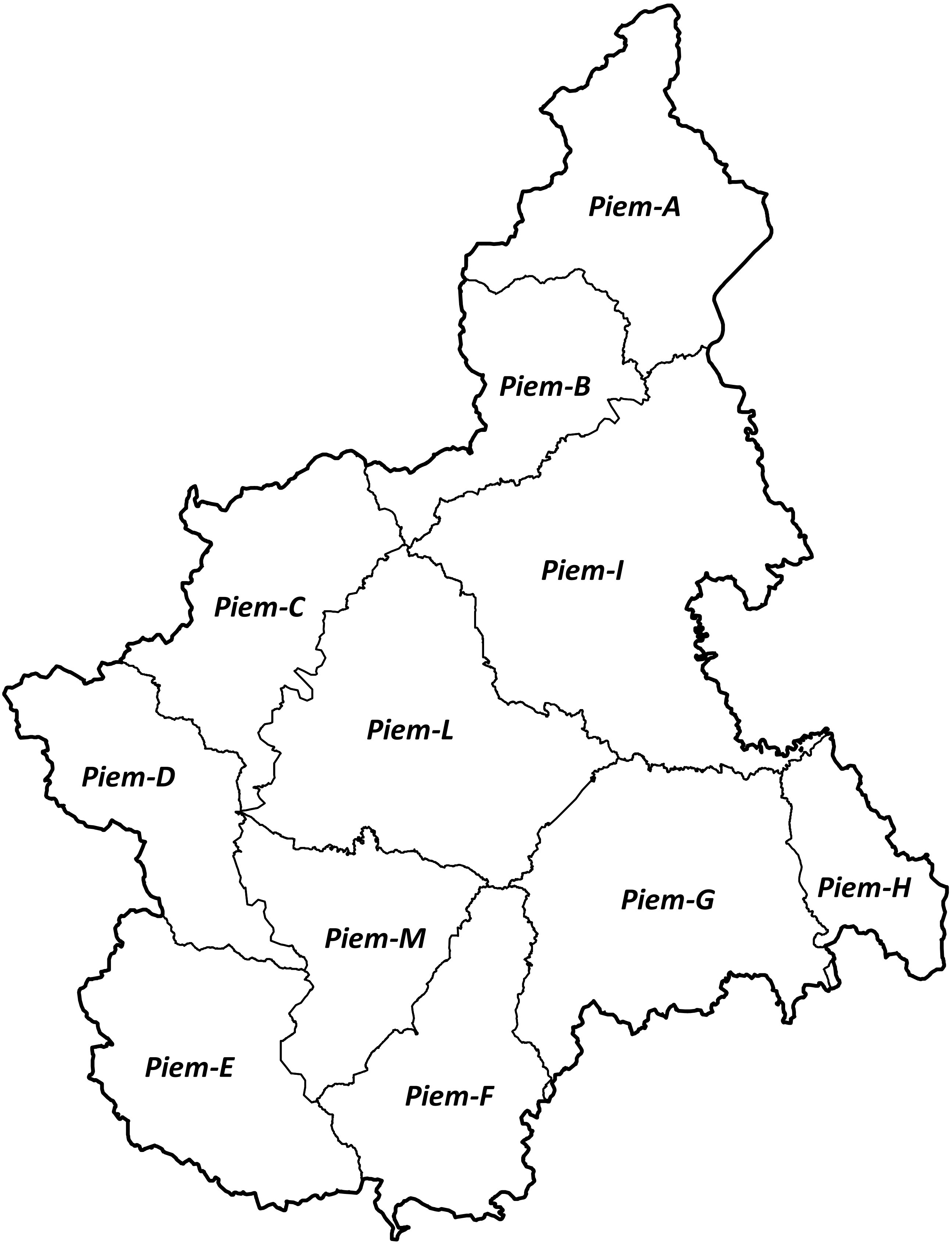

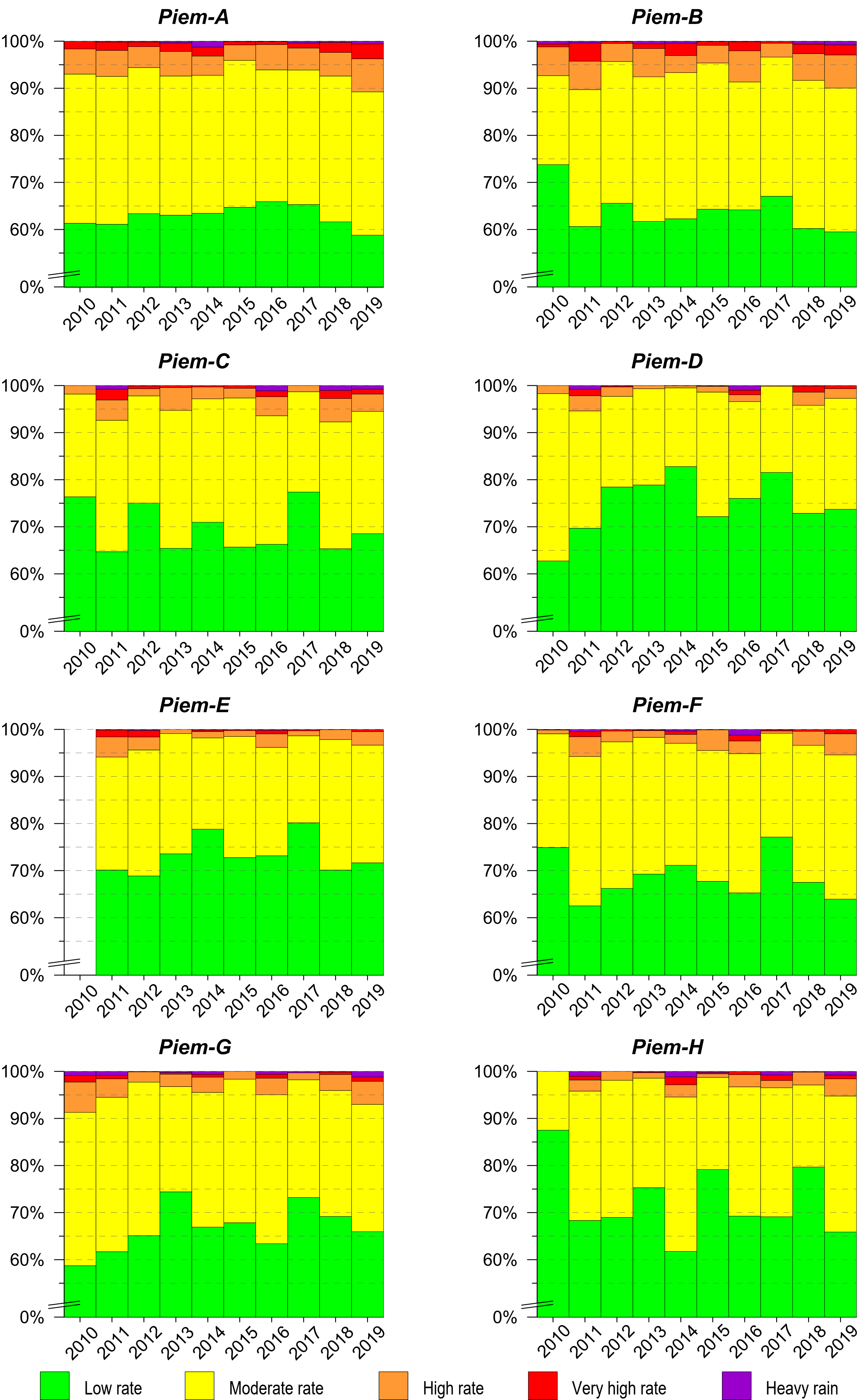

Piem-A
Piem-B
Piem-C
Piem-D
Piem-E
Piem-F
Piem-G
Piem-H
100%
90%
80%
70%
60%
0%
2010
2011
2012
2013
2014
2015
2016
2017
2018
2019
Low rate
Moderate rate
High rate
Very high rate
Heavy rain

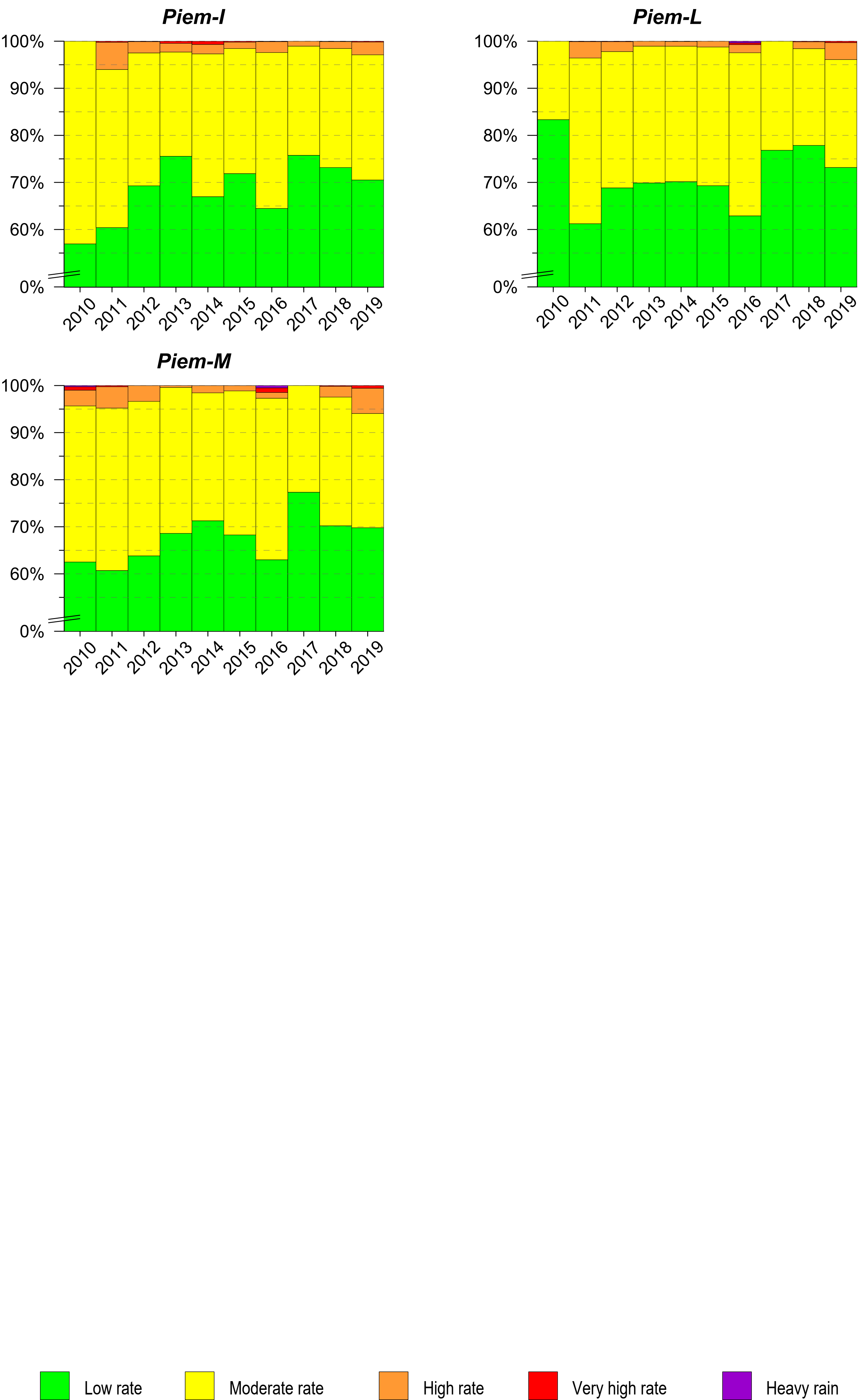
Piem-I
Piem-L
Piem-M
100%
90%
80%
70%
60%
0%
2010
2011
2012
2013
2014
2015
2016
2017
2018
2019
Low rate
Moderate rate
High rate
Very high rate
Heavy rain

# 12 - Puglia Region

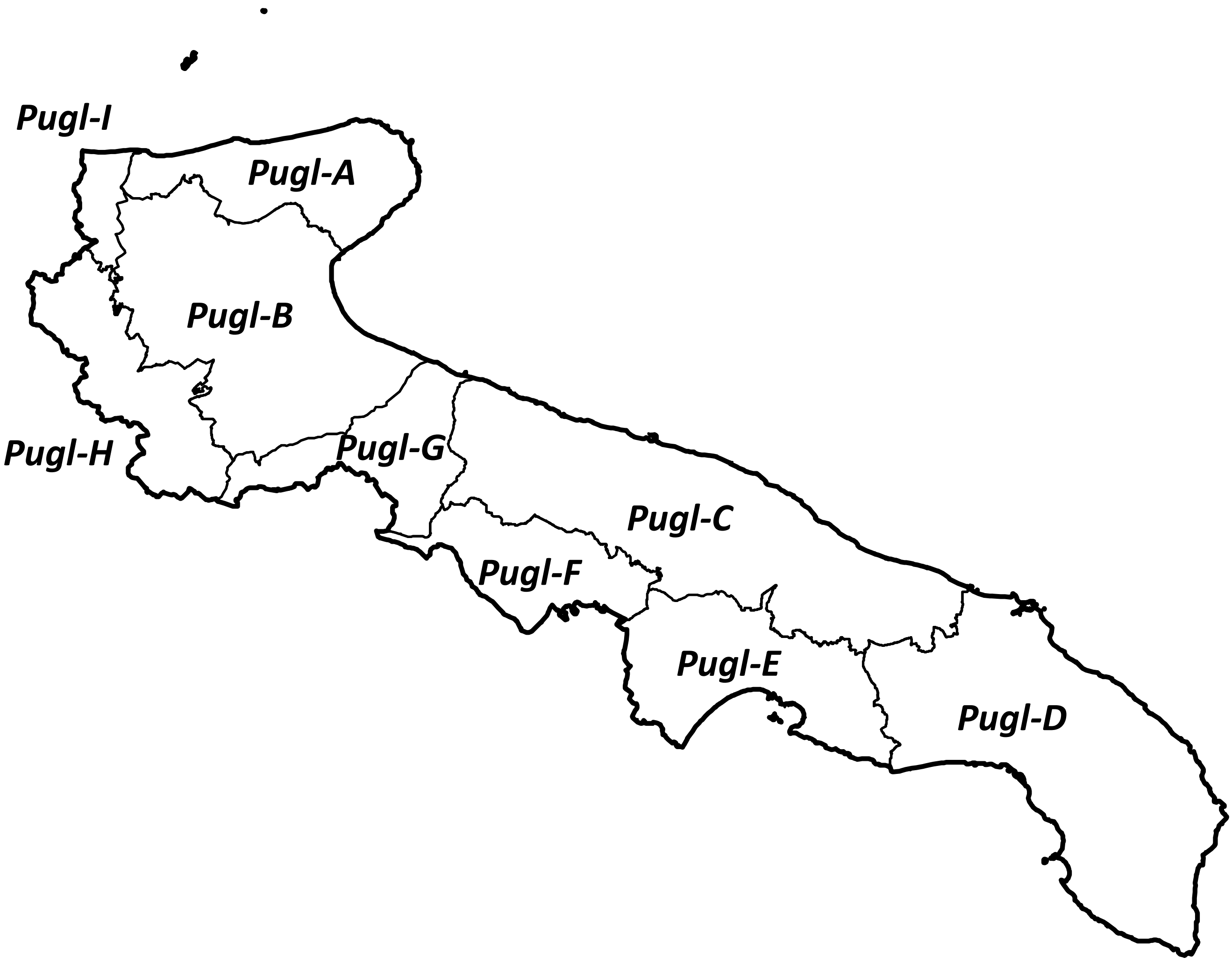

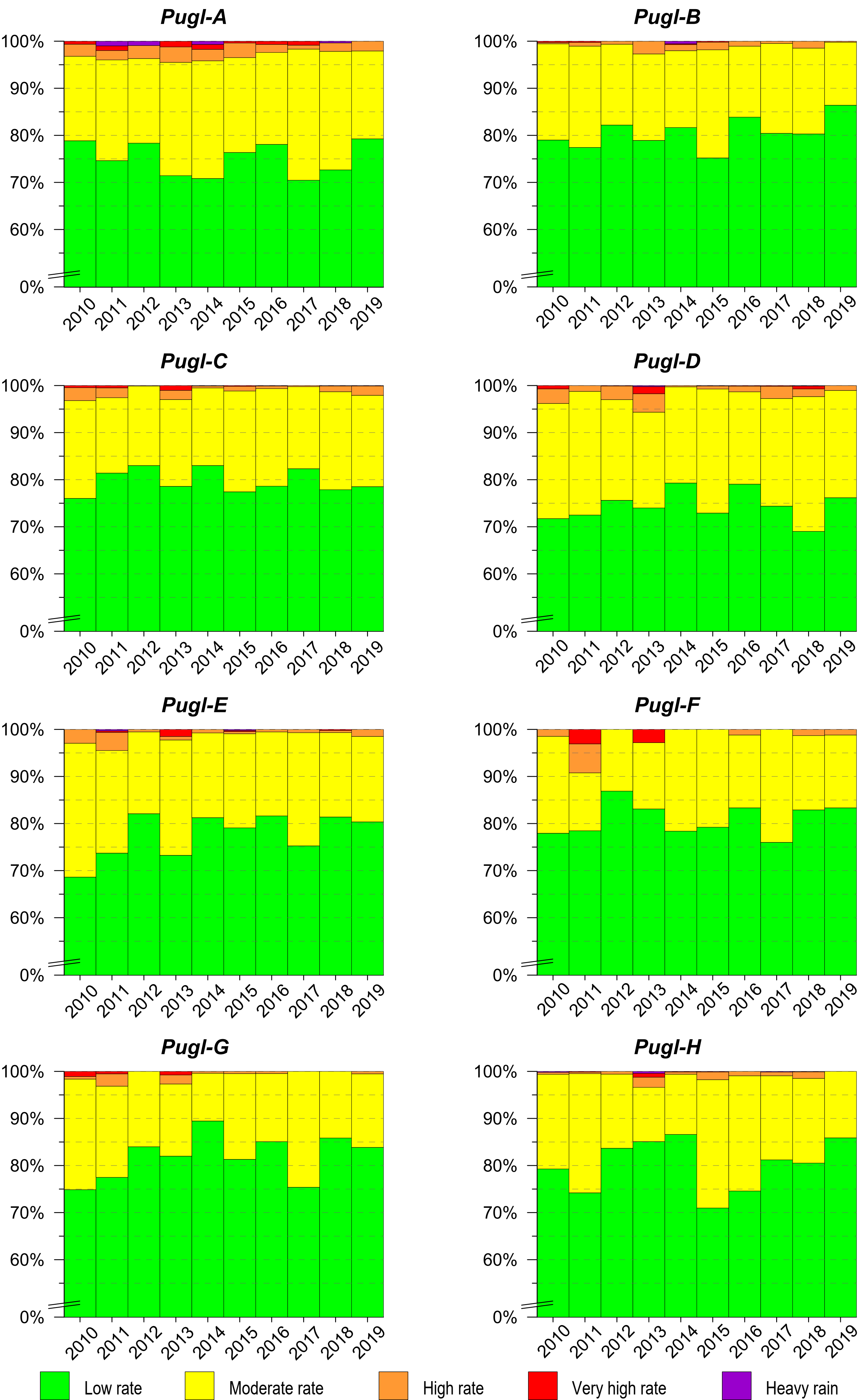
Pugl-A
Pugl-B
Pugl-C
Pugl-D
Pugl-E
Pugl-F
Pugl-G
Pugl-H
100%
90%
80%
70%
60%
0%
2010
2011
2012
2013
2014
2015
2016
2017
2018
2019
Low rate
Moderate rate
High rate
Very high rate
Heavy rain

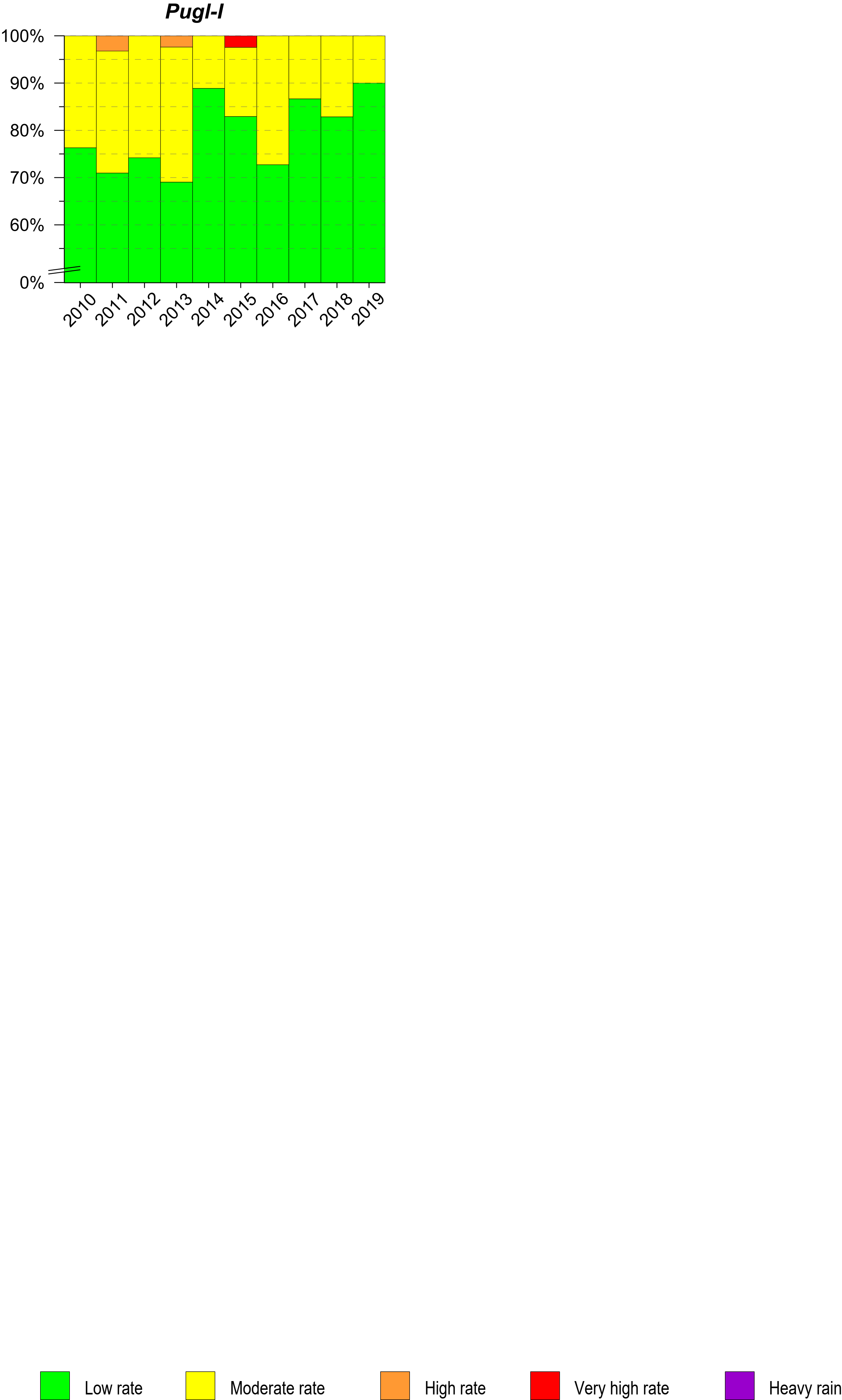
Pugl-I
100%
90%
80%
70%
60%
0%
2010
2011
2012
2013
2014
2015
2016
2017
2018
2019
Low rate
Moderate rate
High rate
Very high rate
Heavy rain

# 20 - Sardegna Region

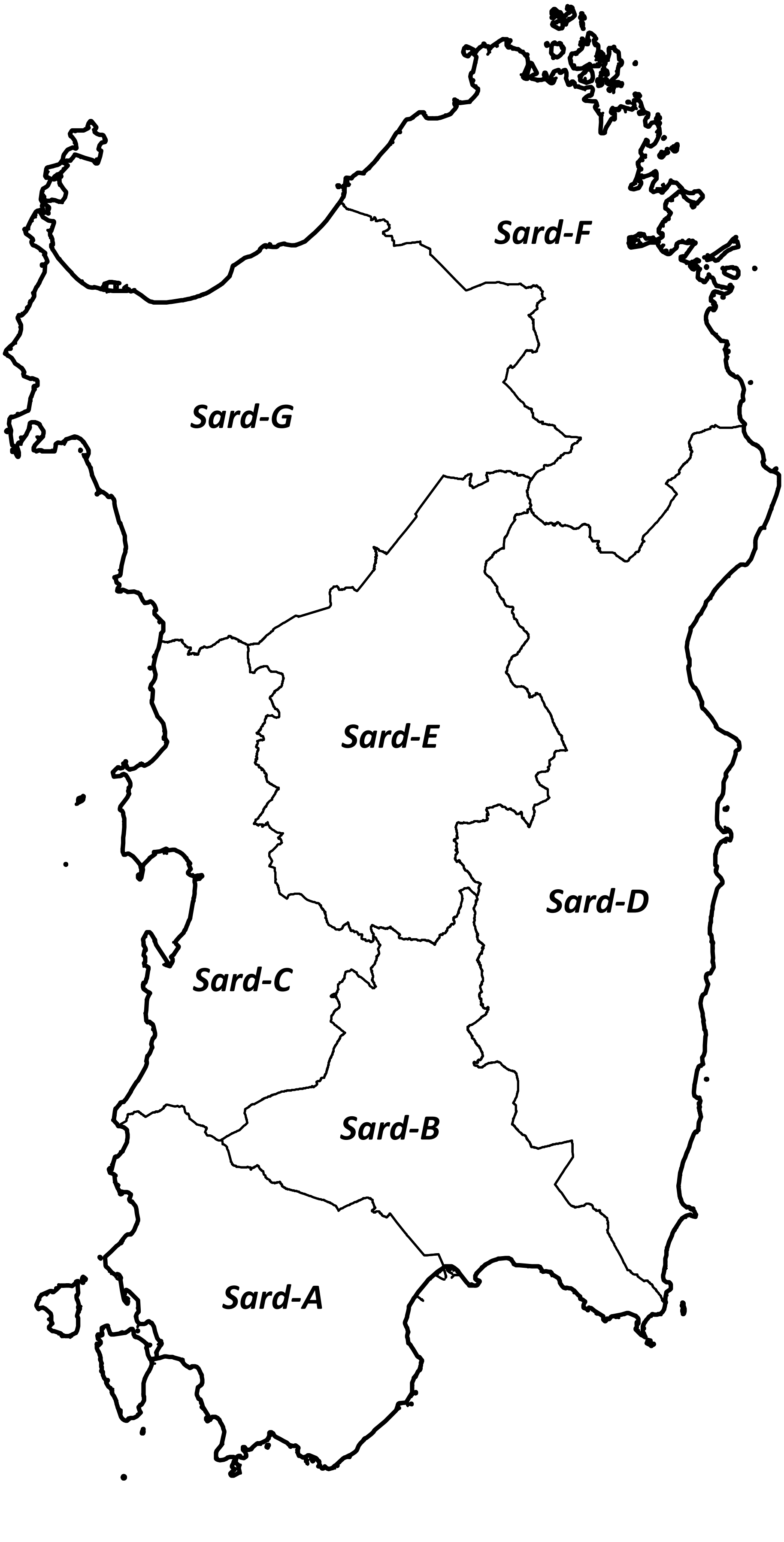

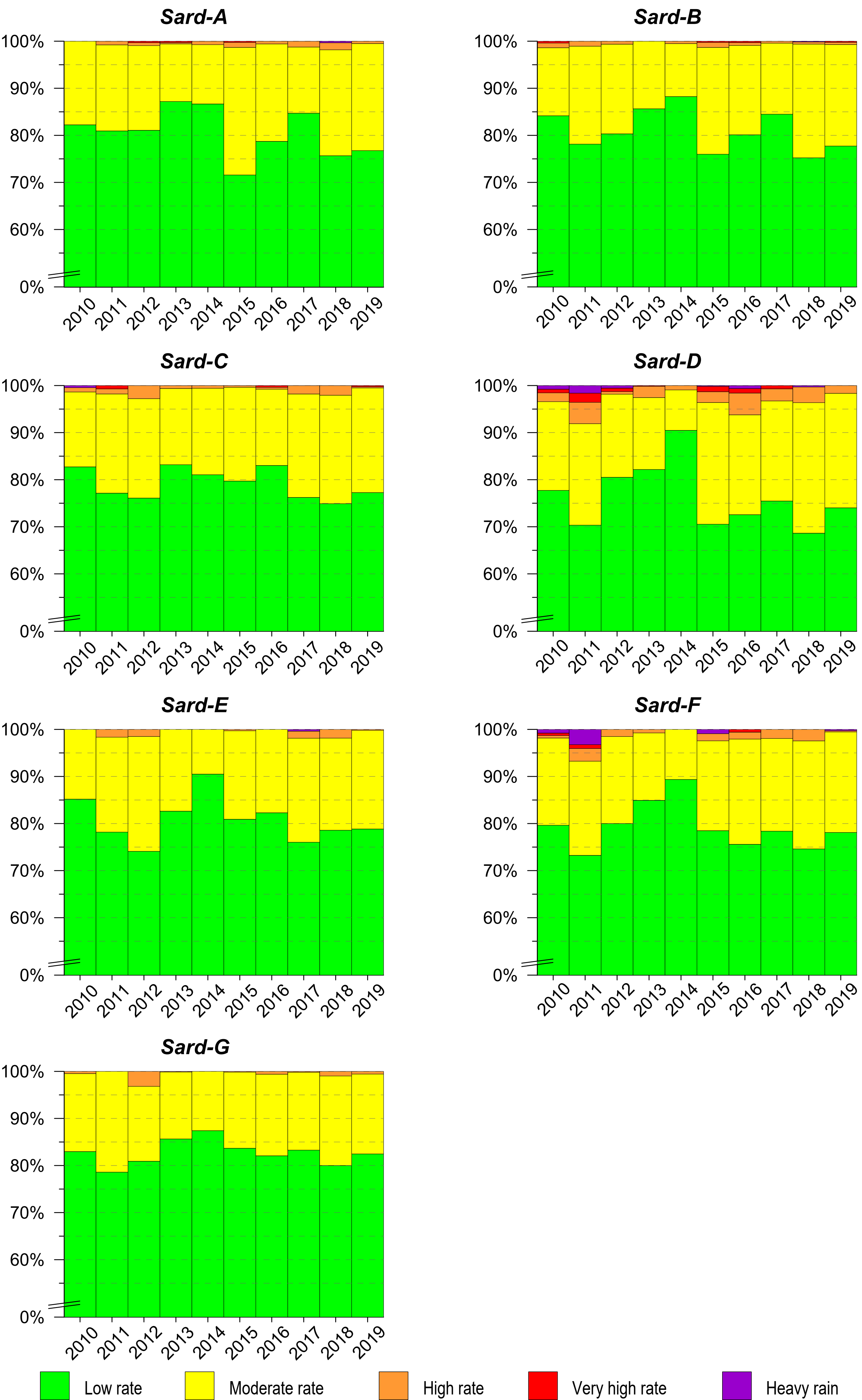
Sard-A
Sard-B
Sard-C
Sard-D
Sard-E
Sard-F
Sard-G
100%
90%
80%
70%
60%
0%
2010
2011
2012
2013
2014
2015
2016
2017
2018
2019
Low rate
Moderate rate
High rate
Very high rate
Heavy rain

# 19 - Sicilia Region

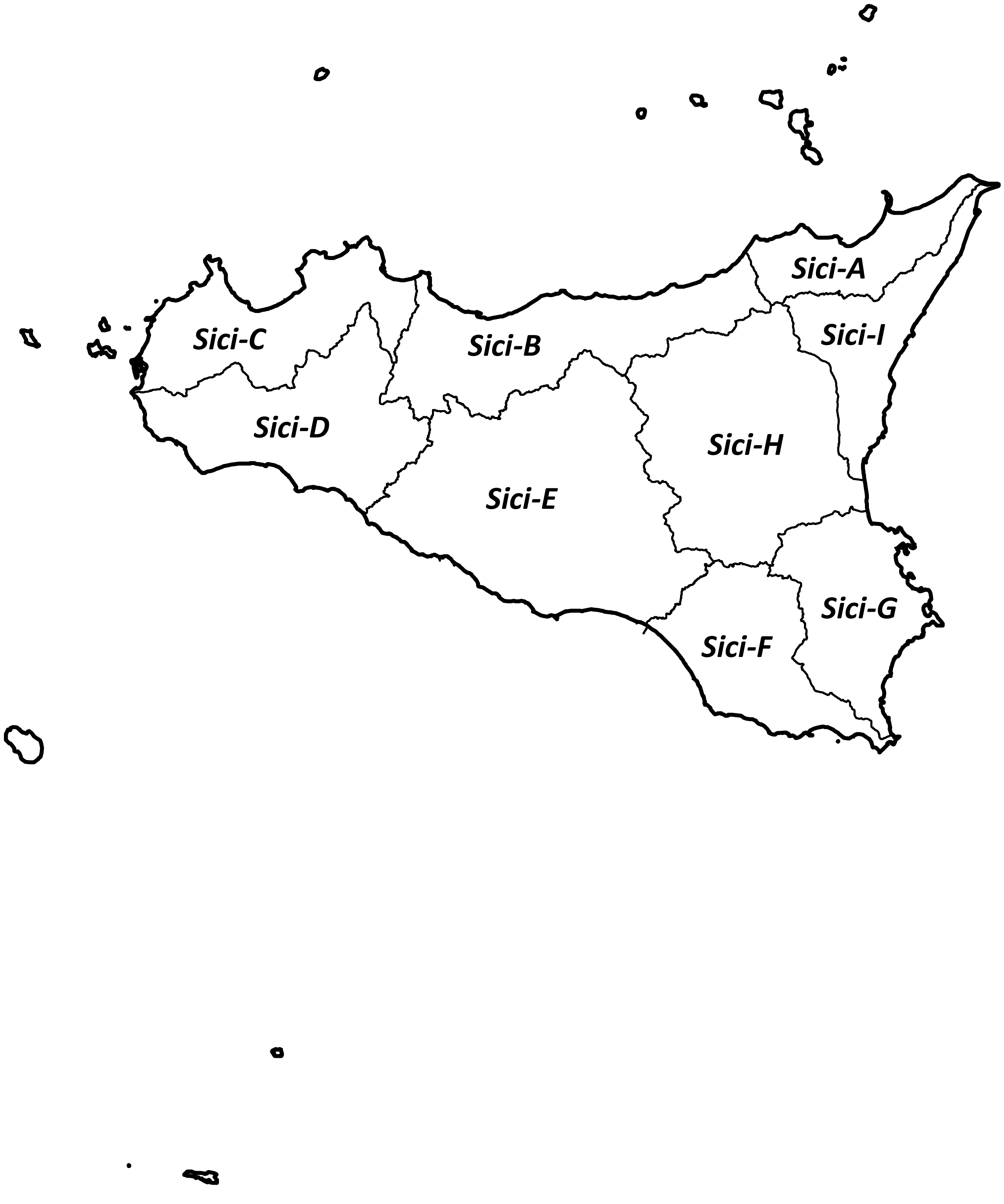

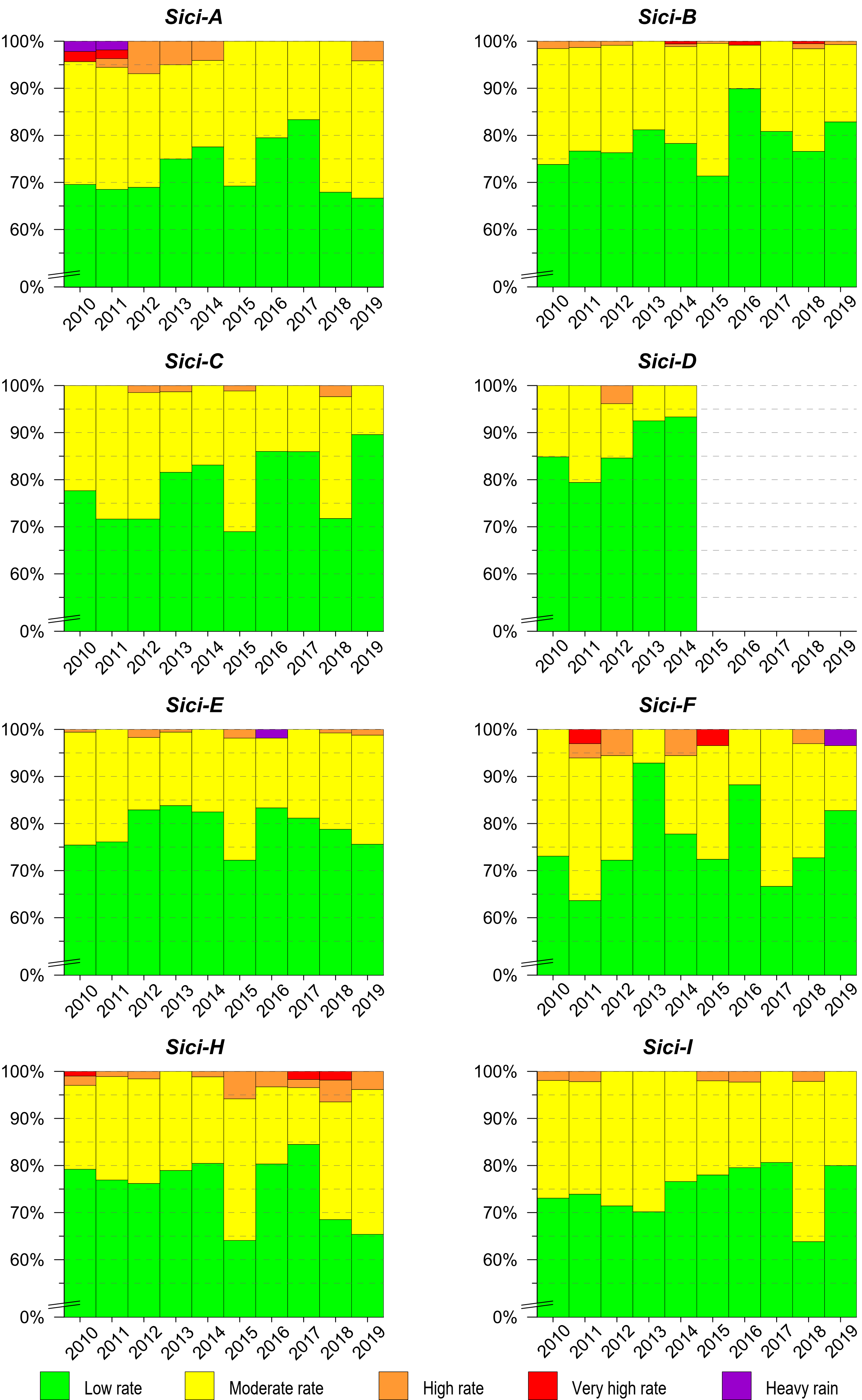

Sici-A
Sici-B
Sici-C
Sici-D
Sici-E
Sici-F
Sici-H
Sici-I
100%
90%
80%
70%
60%
0%
2010
2011
2012
2013
2014
2015
2016
2017
2018
2019
Low rate
Moderate rate
High rate
Very high rate
Heavy rain

# 17 - Toscana Region

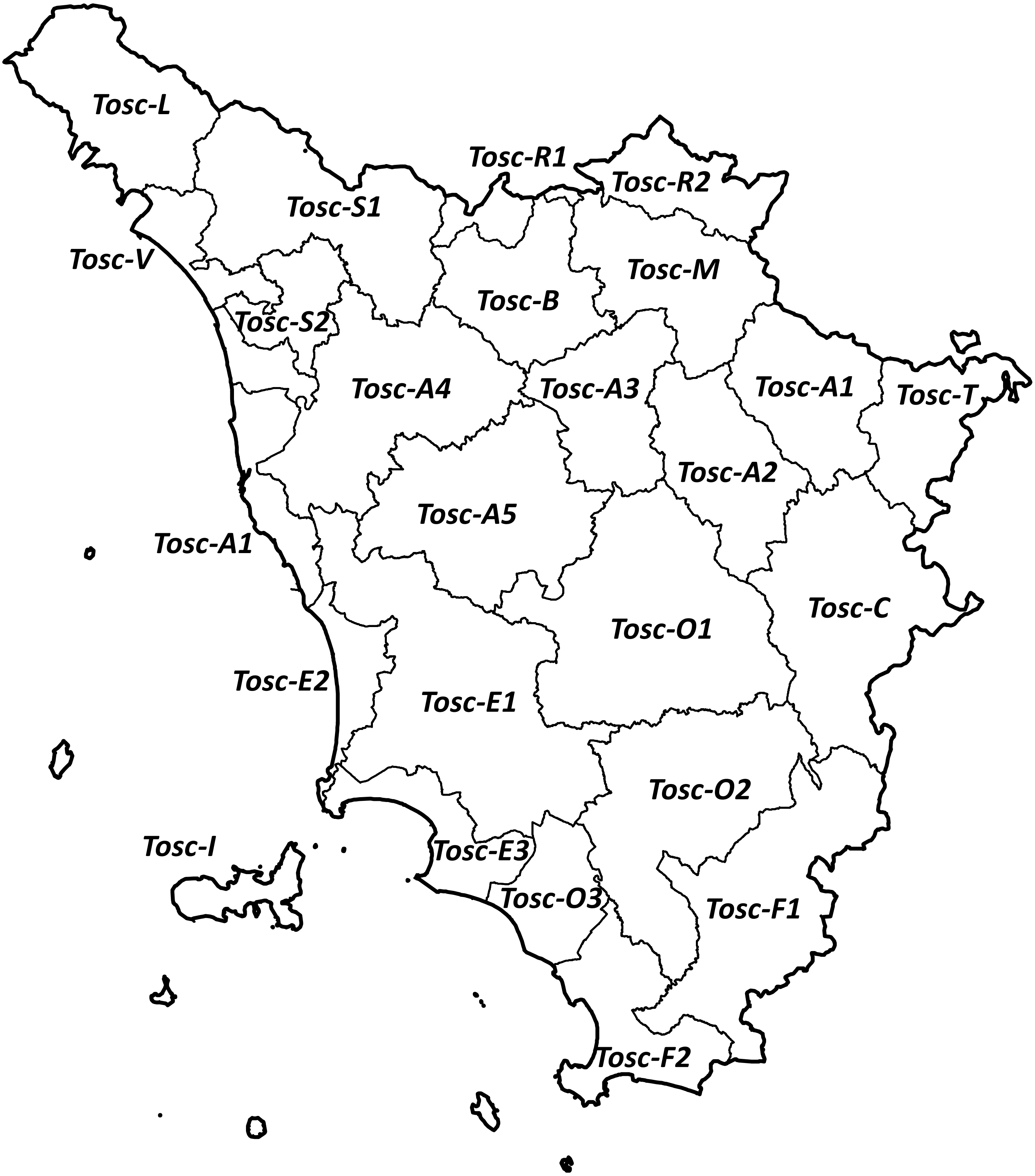

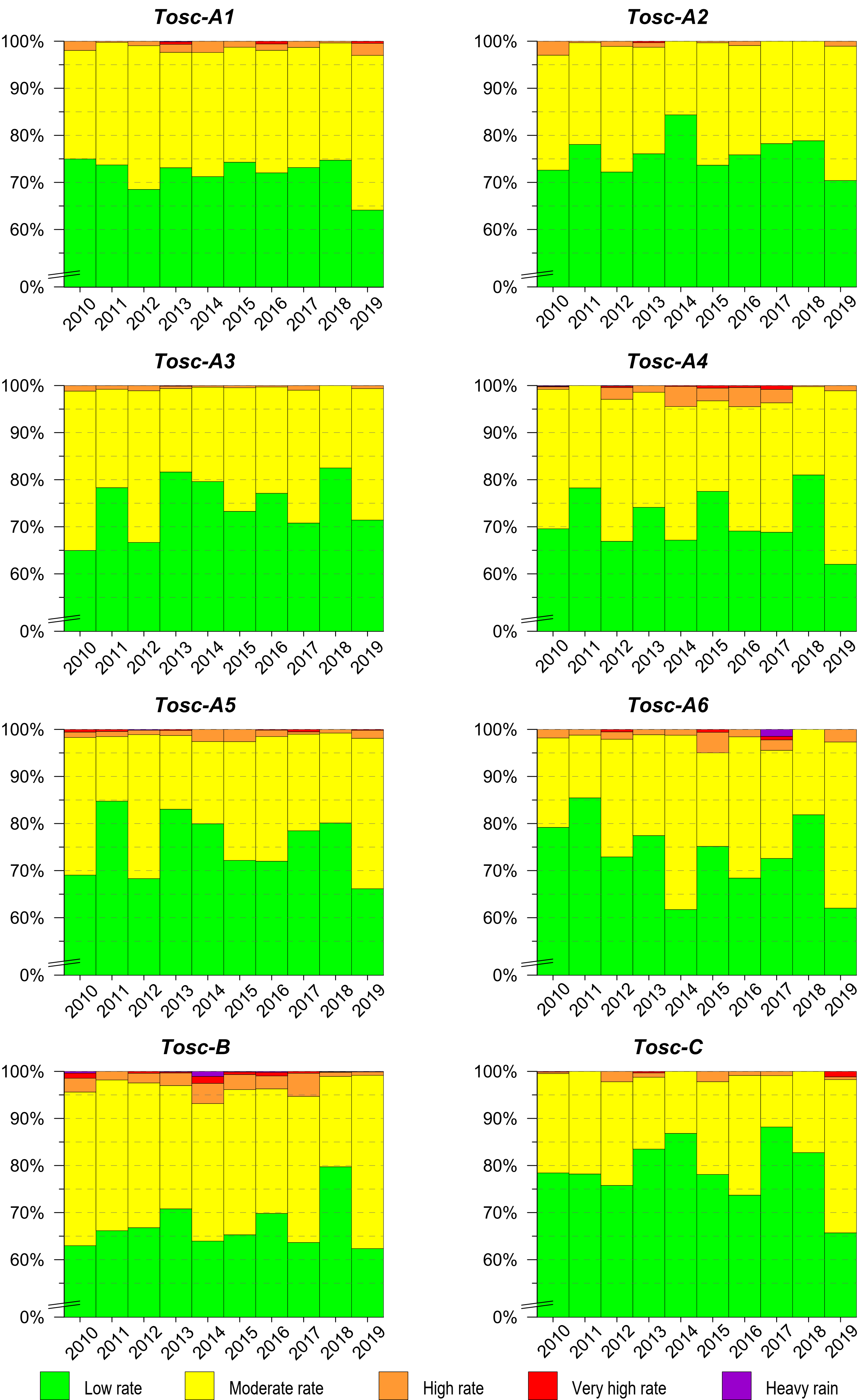
Tosc-A1
Tosc-A2
Tosc-A3
Tosc-A4
Tosc-A5
Tosc-A6
Tosc-B
Tosc-C
100%
90%
80%
70%
60%
0%
2010
2011
2012
2013
2014
2015
2016
2017
2018
2019
Low rate
Moderate rate
High rate
Very high rate
Heavy rain

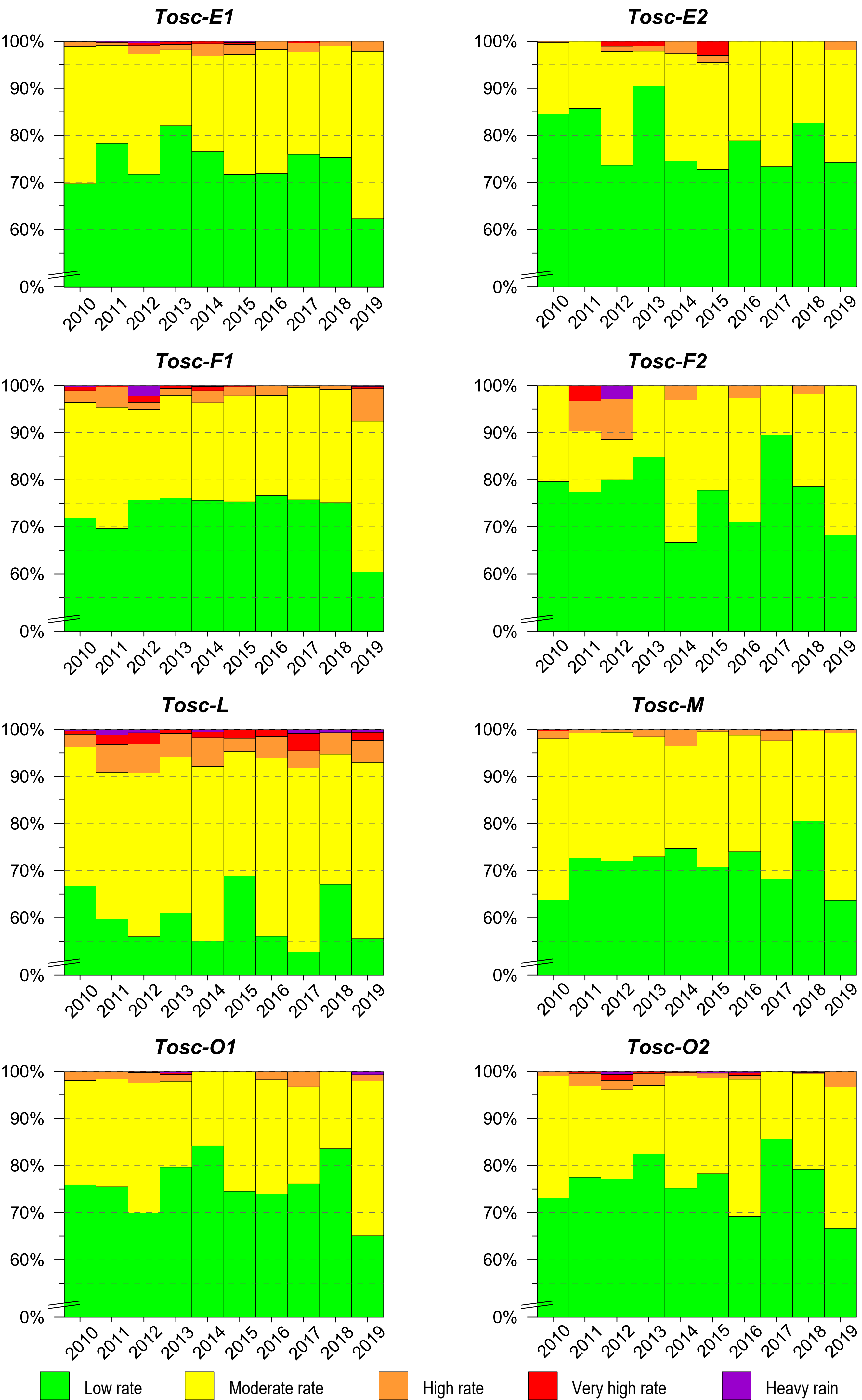

Tosc-E1
Tosc-E2
Tosc-F1
Tosc-F2
Tosc-L
Tosc-M
Tosc-O1
Tosc-O2
100%
90%
80%
70%
60%
0%
2010
2011
2012
2013
2014
2015
2016
2017
2018
2019
Low rate
Moderate rate
High rate
Very high rate
Heavy rain

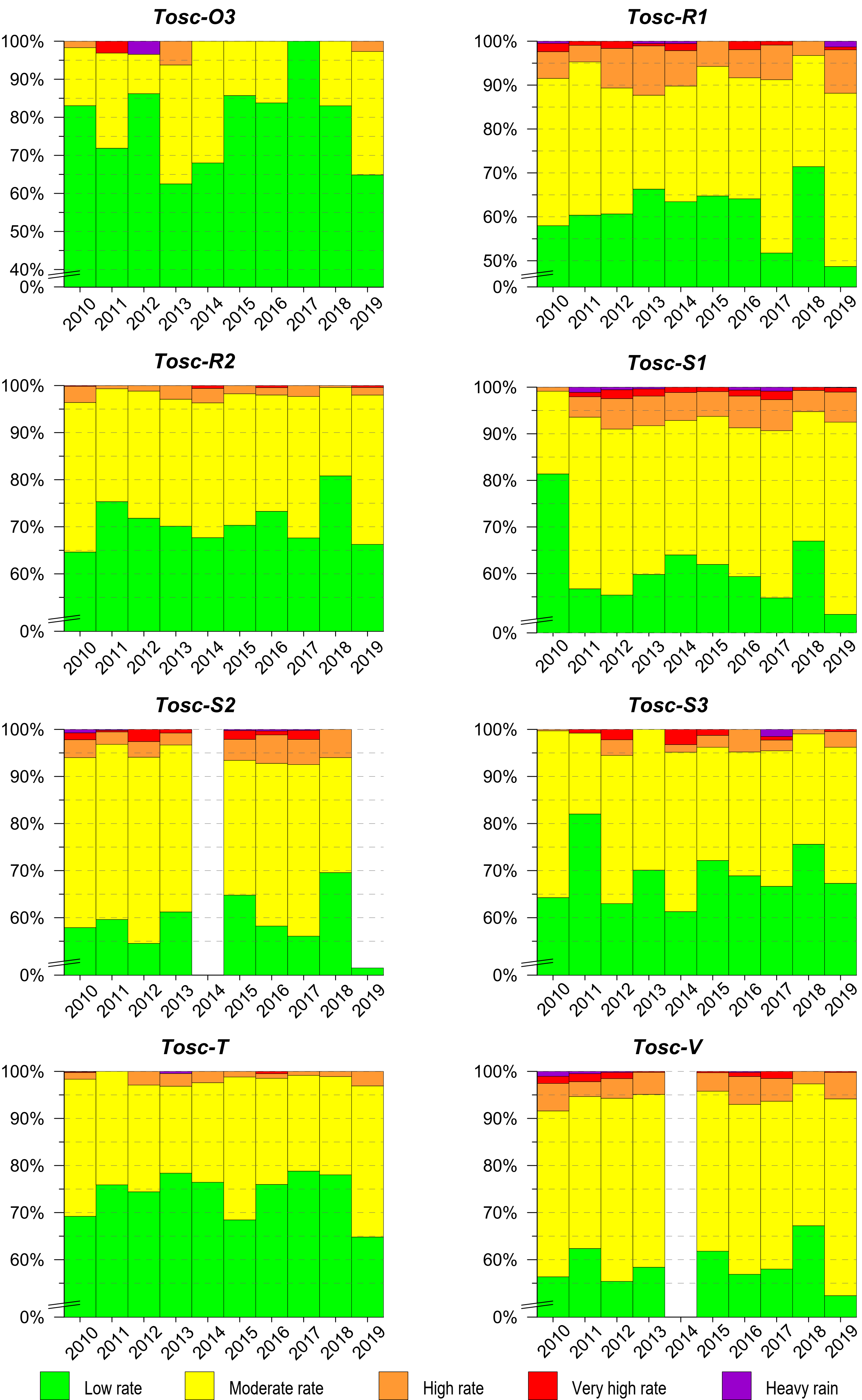

Tosc-O3
Tosc-R1
Tosc-R2
Tosc-S1
Tosc-S2
Tosc-S3
Tosc-T
Tosc-V
100%
90%
80%
70%
60%
50%
40%
0%
2010
2011
2012
2013
2014
2015
2016
2017
2018
2019
Low rate
Moderate rate
High rate
Very high rate
Heavy rain

# 4 - Trentino-Alto Adige Region

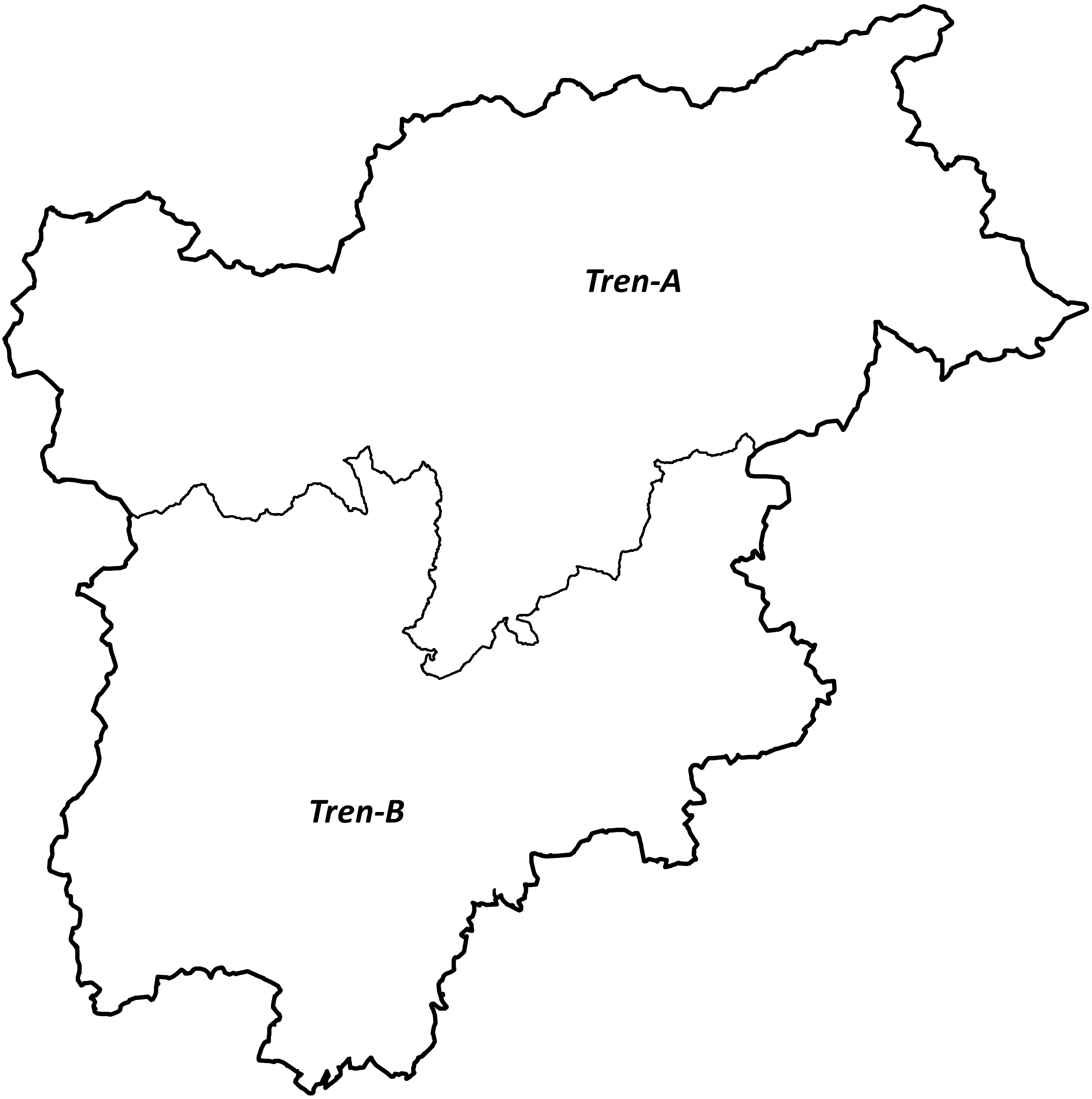

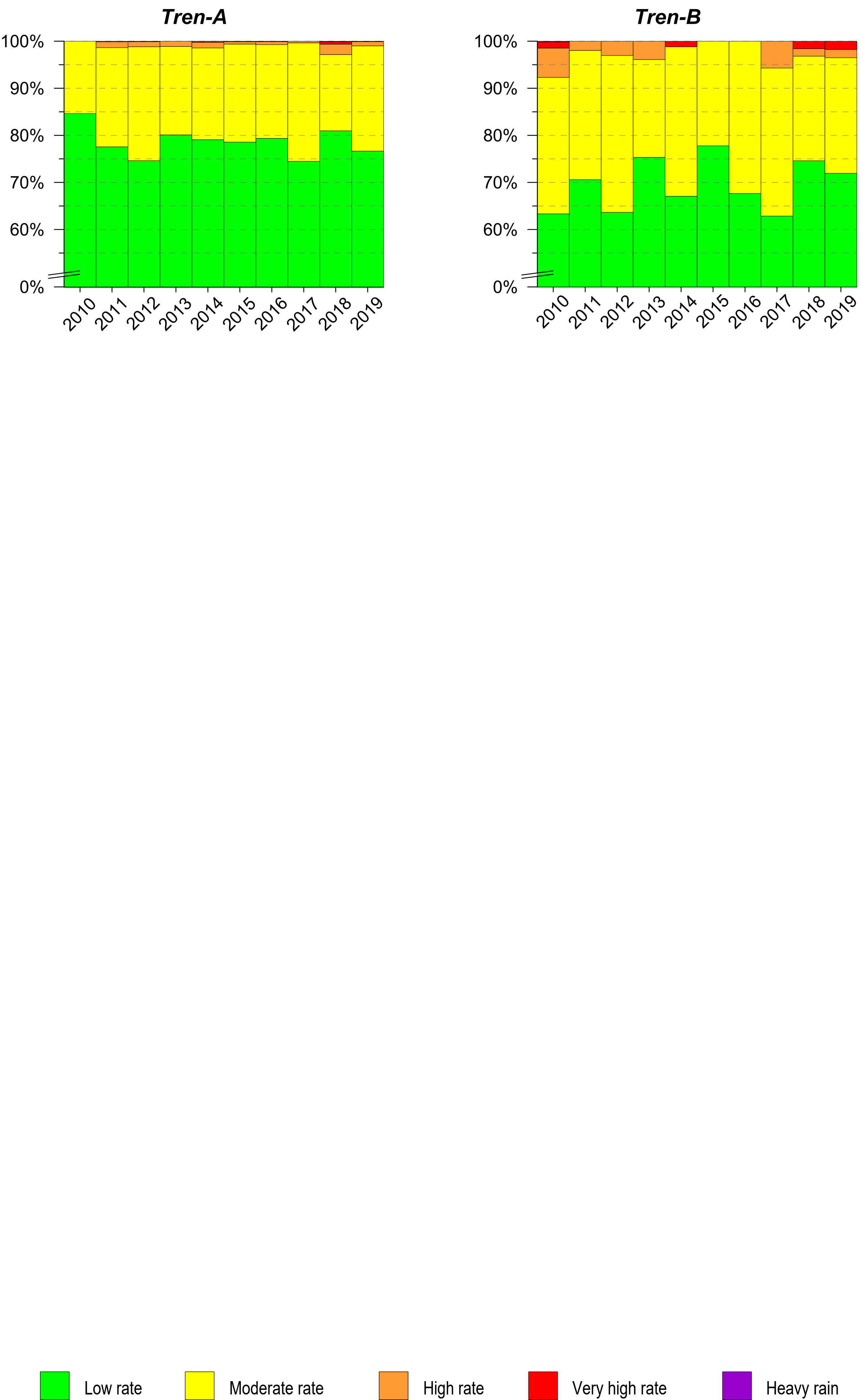
Tren-A
Tren-B
100%
90%
80%
70%
60%
0%
2010
2011
2012
2013
2014
2015
2016
2017
2018
2019
Low rate
Moderate rate
High rate
Very high rate
Heavy rain

# 9 - Umbria Region

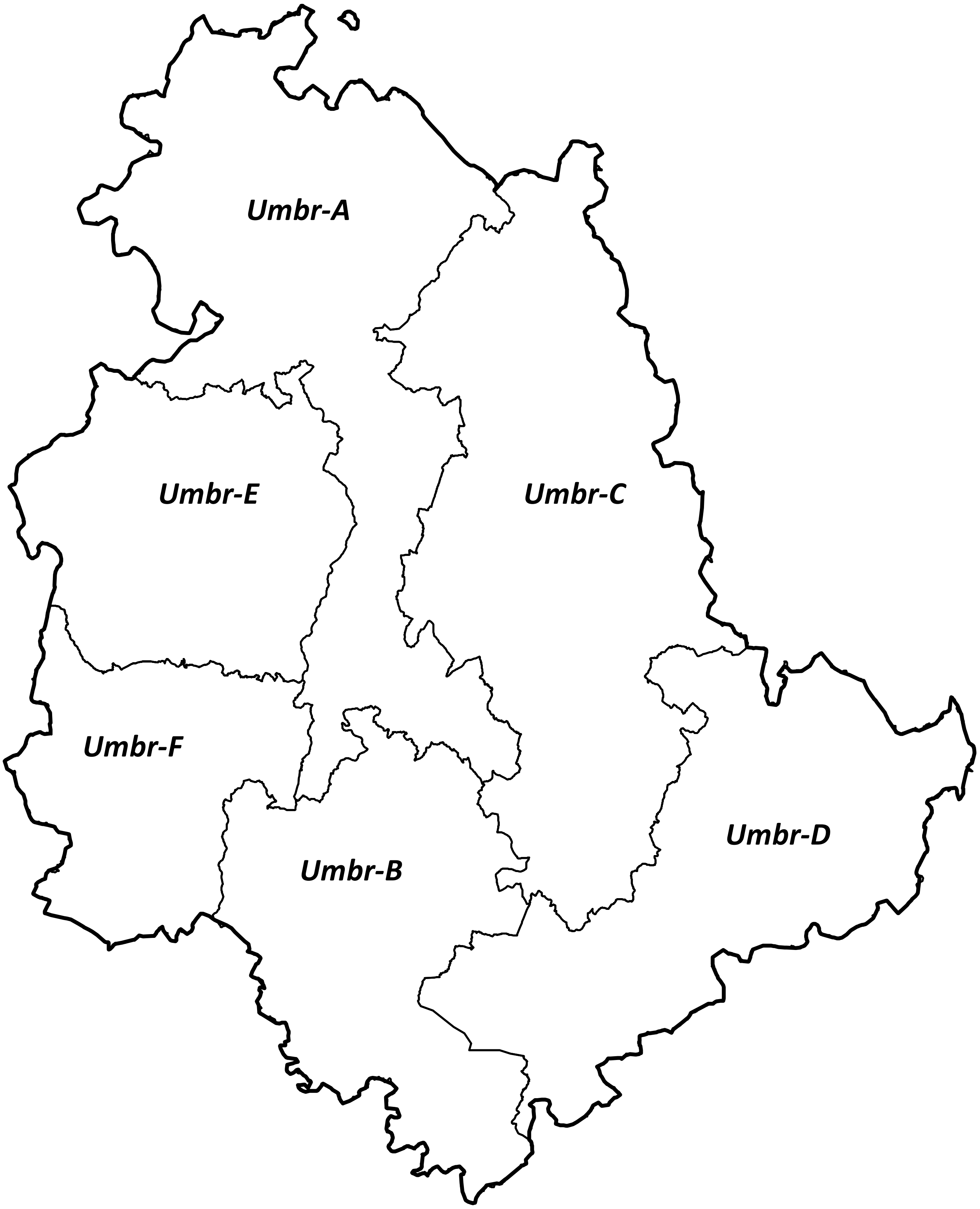

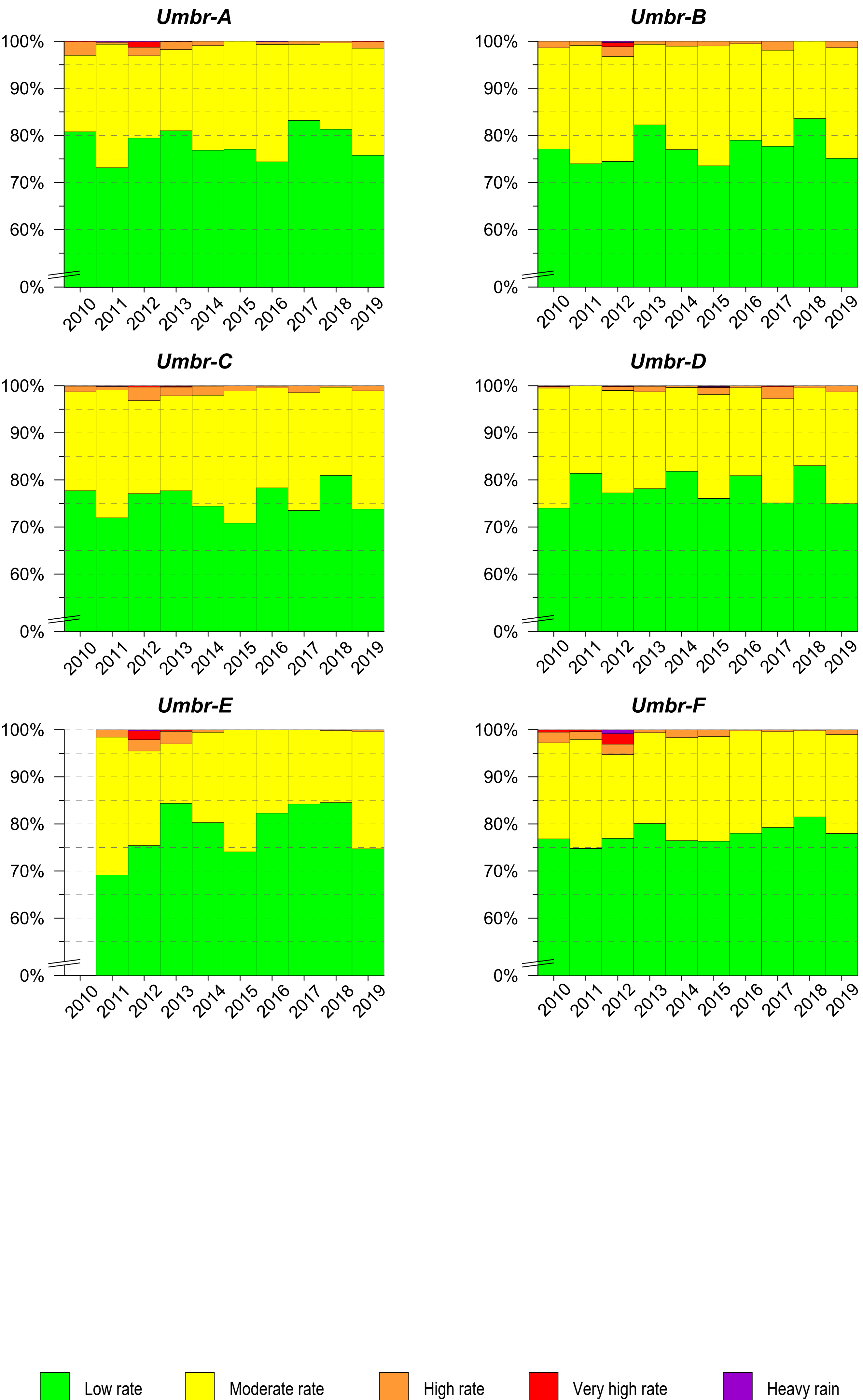

Umbr-A
Umbr-B
Umbr-C
Umbr-D
Umbr-E
Umbr-F
100%
90%
80%
70%
60%
0%
2010
2011
2012
2013
2014
2015
2016
2017
2018
2019
Low rate
Moderate rate
High rate
Very high rate
Heavy rain

# 1 - Valle d'Aosta Region

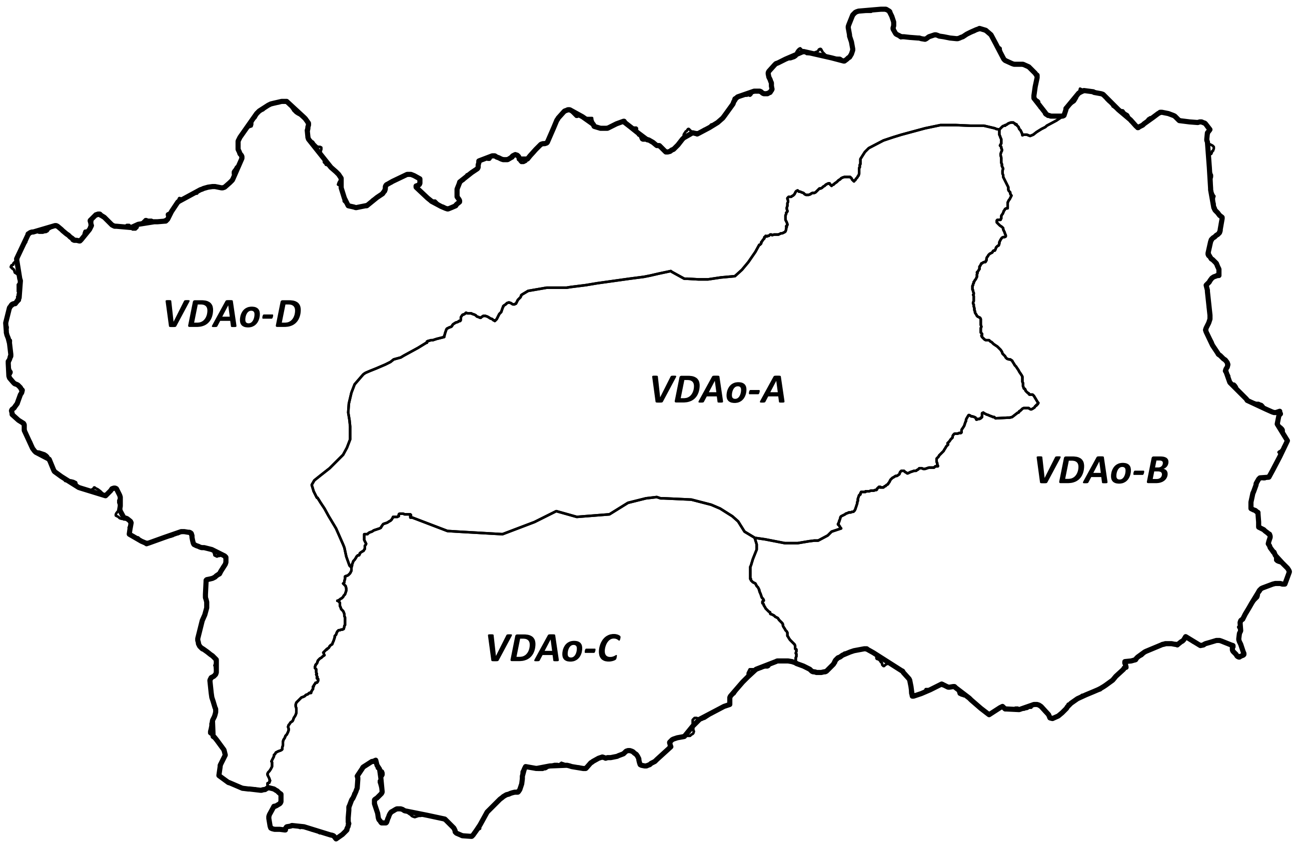

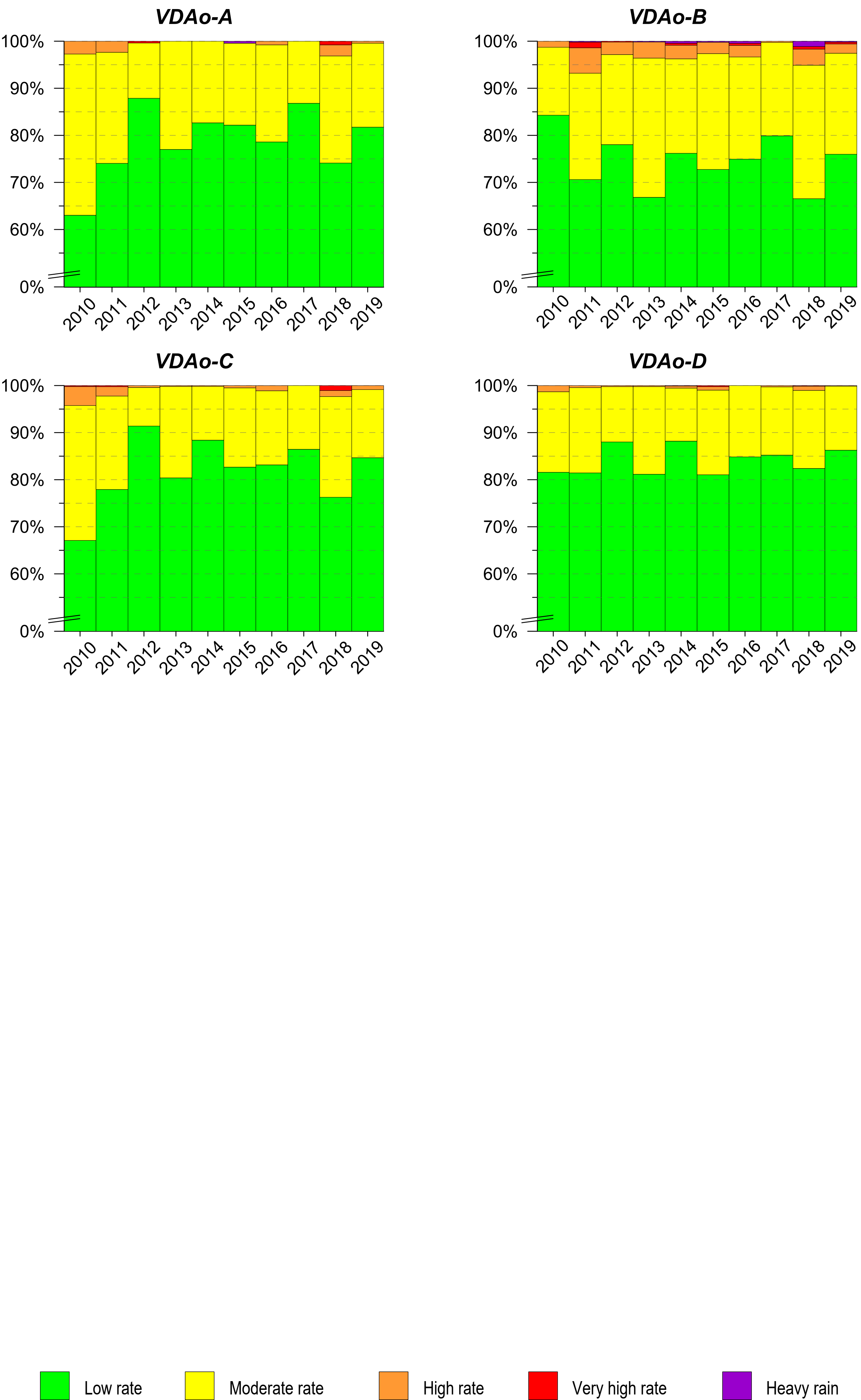
VDAo-A
VDAo-B
VDAo-C
VDAo-D
100%
90%
80%
70%
60%
0%
2010
2011
2012
2013
2014
2015
2016
2017
2018
2019
Low rate
Moderate rate
High rate
Very high rate
Heavy rain

# 5 - Veneto Region

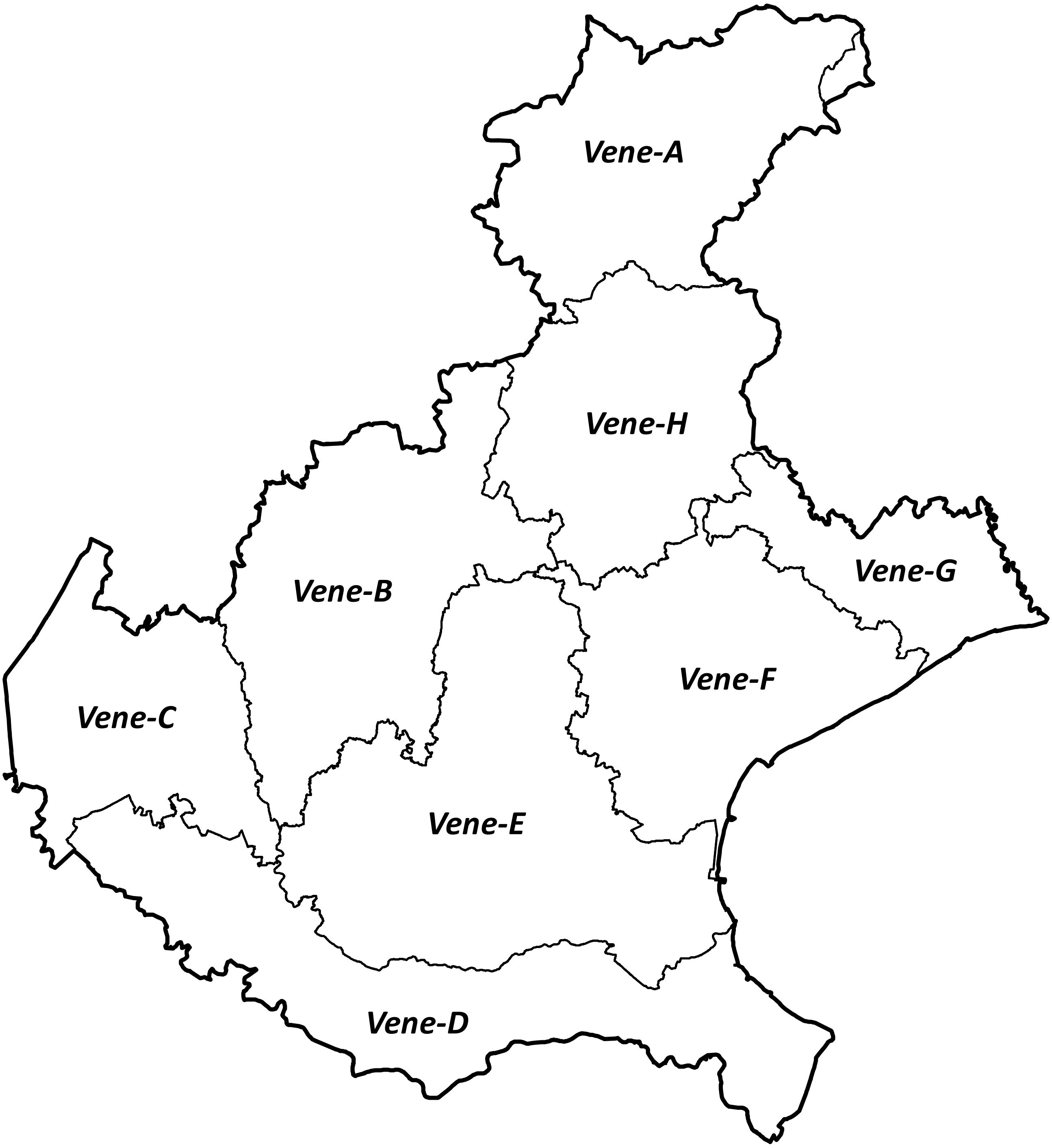

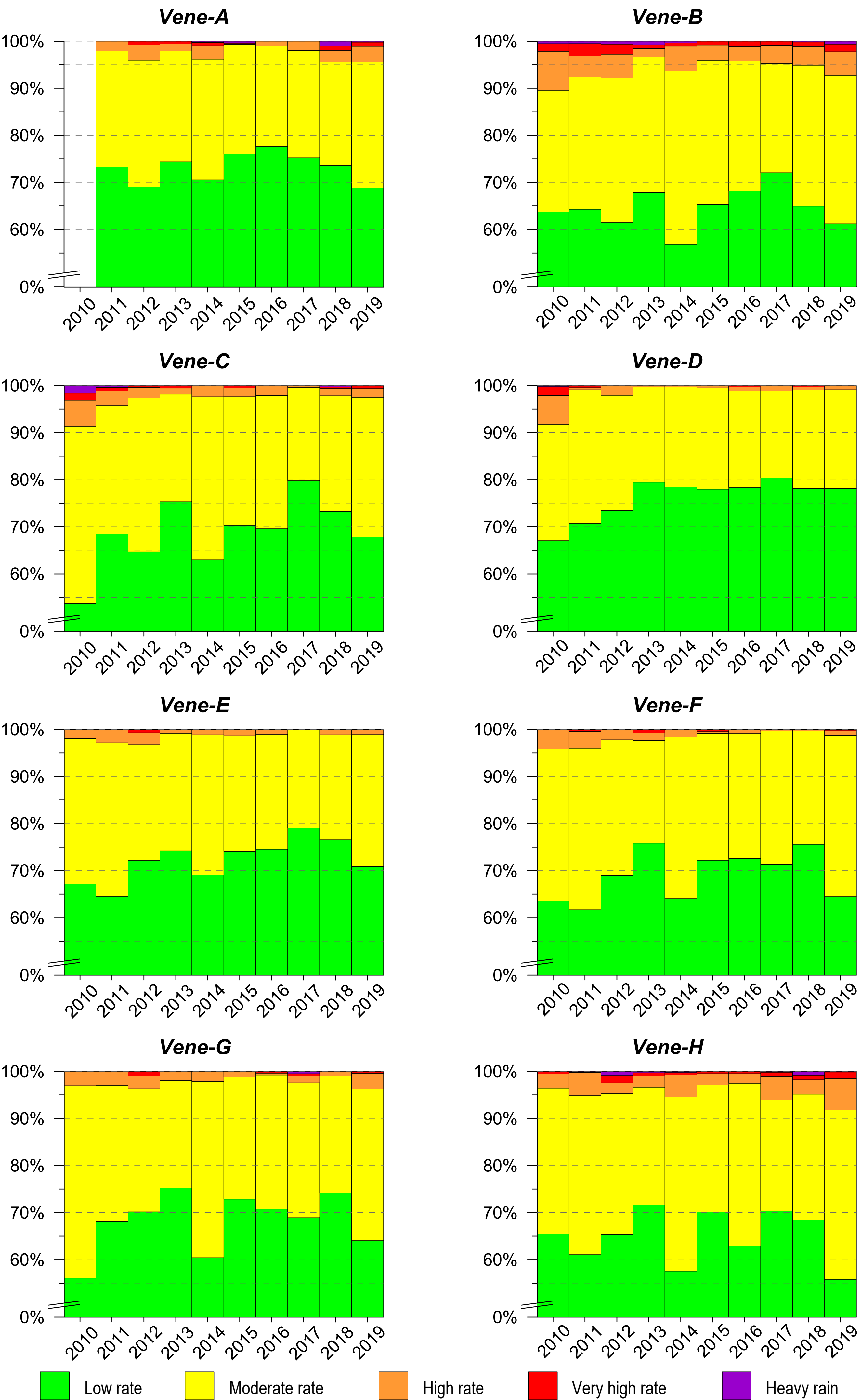
Vene-A
Vene-B
Vene-C
Vene-D
Vene-E
Vene-F
Vene-G
Vene-H
100%
90%
80%
70%
60%
0%
2010
2011
2012
2013
2014
2015
2016
2017
2018
2019
Low rate
Moderate rate
High rate
Very high rate
Heavy rain
