# Supplementary material for: Ten years of pluviometric analyses in Italy for civil protection purposes
Source: Sci Rep. 2021 Oct 13;11:20302. doi: 10.1038/s41598-021-99874-w (PMC8514502; doi:10.1038/s41598-021-99874-w)
Supplement: Supplementary file 3 — Supplementary Information 3. [file 41598_2021_99874_MOESM3_ESM.pdf]

Supplementary material

Suppl. 2B

Frequency events > 150 mm/day

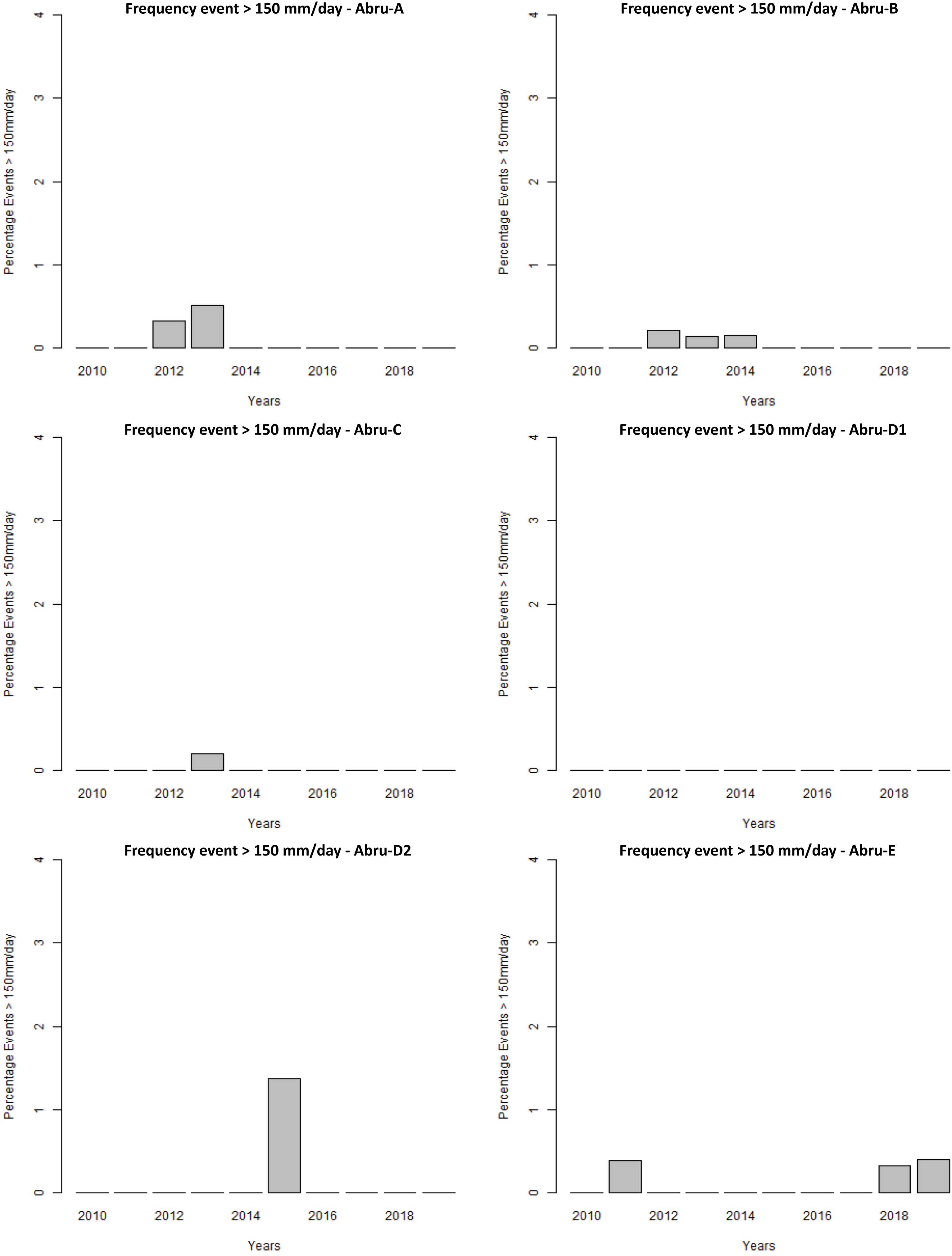
Frequency event > 150 mm/day - Abru-A
Percentage Events > 150mm/day
Years
0
1
2
3
4
2010
2012
2014
2016
2018
Frequency event > 150 mm/day - Abru-B
Percentage Events > 150mm/day
Years
0
1
2
3
4
2010
2012
2014
2016
2018
Frequency event > 150 mm/day - Abru-C
Percentage Events > 150mm/day
Years
0
1
2
3
4
2010
2012
2014
2016
2018
Frequency event > 150 mm/day - Abru-D1
Percentage Events > 150mm/day
Years
0
1
2
3
4
2010
2012
2014
2016
2018
Frequency event > 150 mm/day - Abru-D2
Percentage Events > 150mm/day
Years
0
1
2
3
4
2010
2012
2014
2016
2018
Frequency event > 150 mm/day - Abru-E
Percentage Events > 150mm/day
Years
0
1
2
3
4
2010
2012
2014
2016
2018

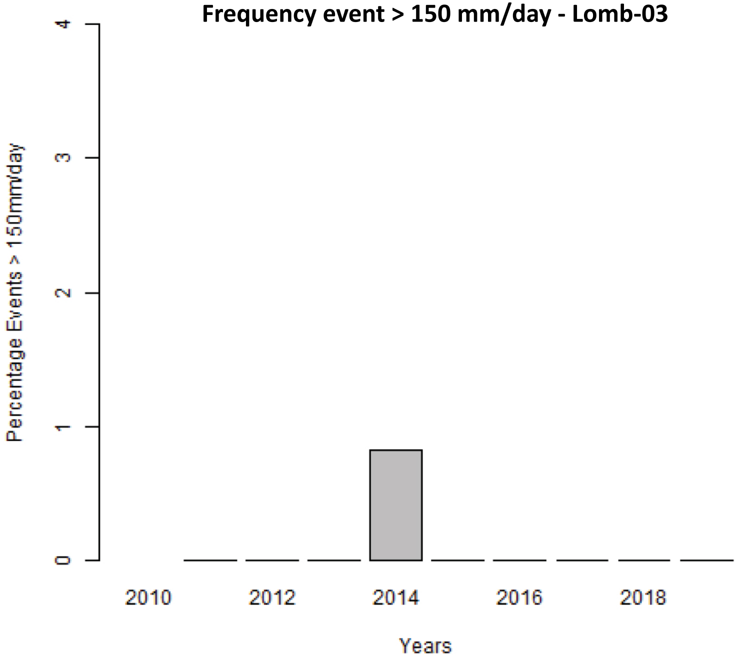
Frequency event > 150 mm/day - Lomb-03
Percentage Events > 150mm/day
0
1
2
3
4
2010
2012
2014
2016
2018
Years

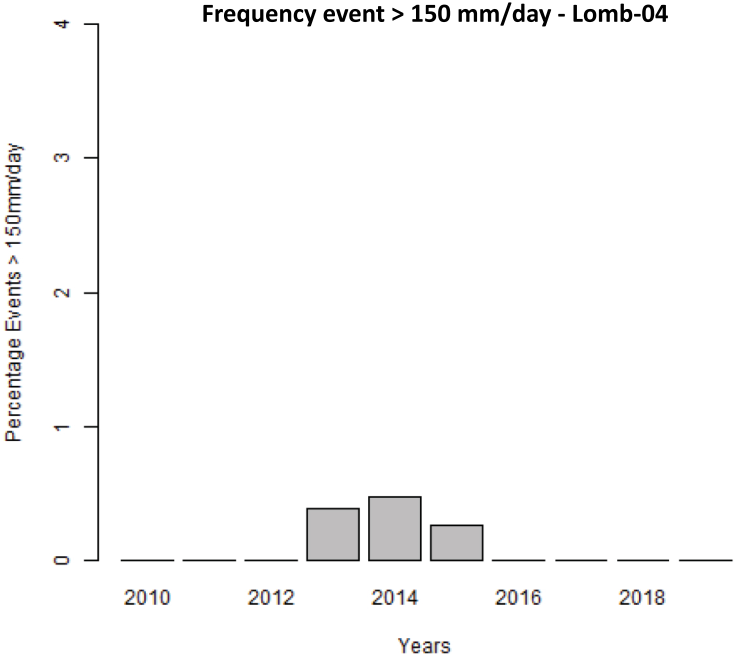
Frequency event > 150 mm/day - Lomb-04
Percentage Events > 150mm/day
0
1
2
3
4
2010
2012
2014
2016
2018
Years

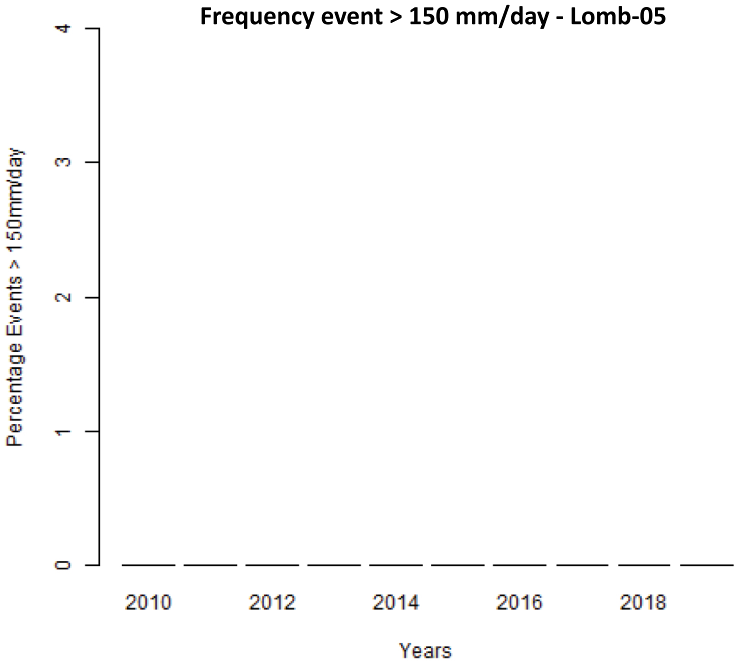
Frequency event > 150 mm/day - Lomb-05
Percentage Events > 150mm/day
0
1
2
3
4
2010
2012
2014
2016
2018
Years

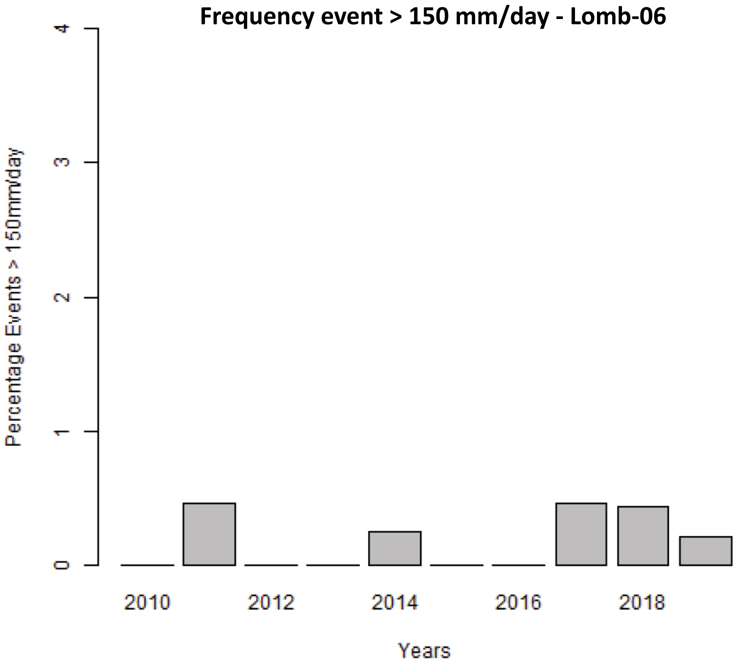
Frequency event > 150 mm/day - Lomb-06
Percentage Events > 150mm/day
0
1
2
3
4
2010
2012
2014
2016
2018
Years

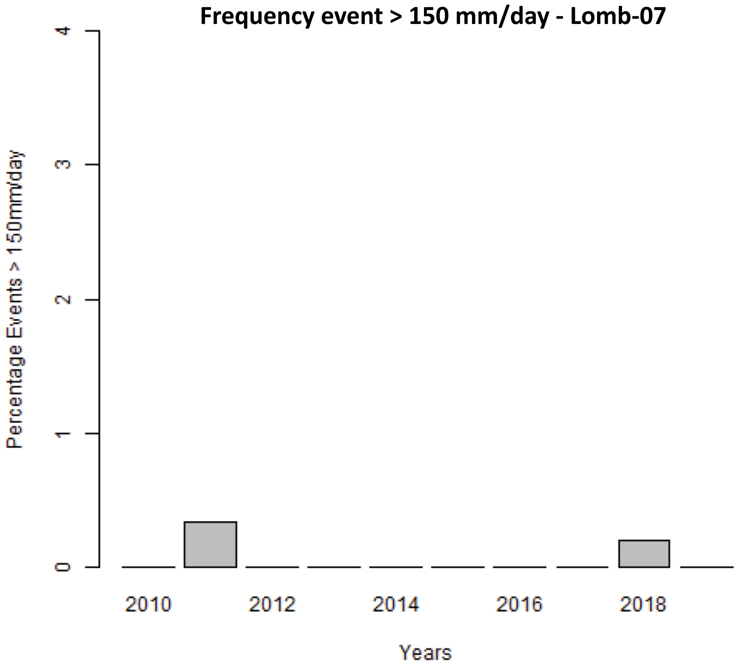
Frequency event > 150 mm/day - Lomb-07
Percentage Events > 150mm/day
0
1
2
3
4
2010
2012
2014
2016
2018
Years

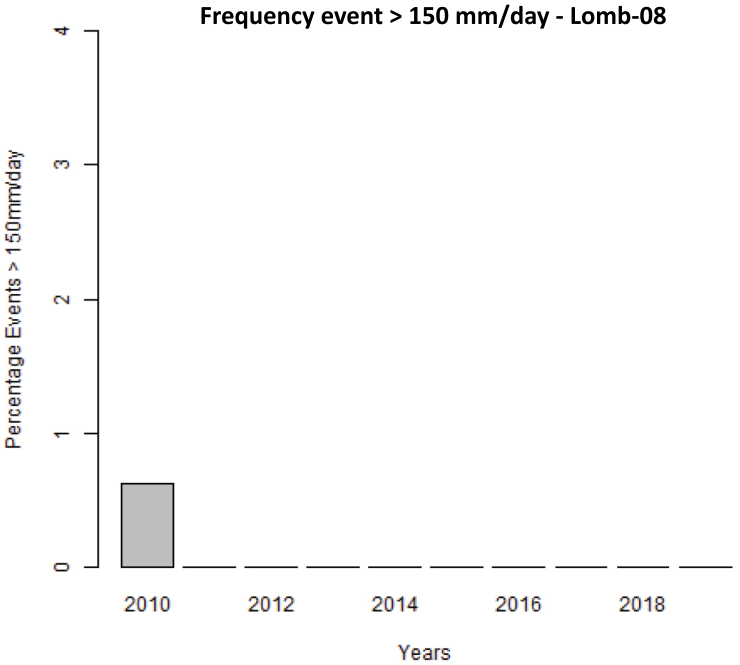
Frequency event > 150 mm/day - Lomb-08
Percentage Events > 150mm/day
0
1
2
3
4
2010
2012
2014
2016
2018
Years

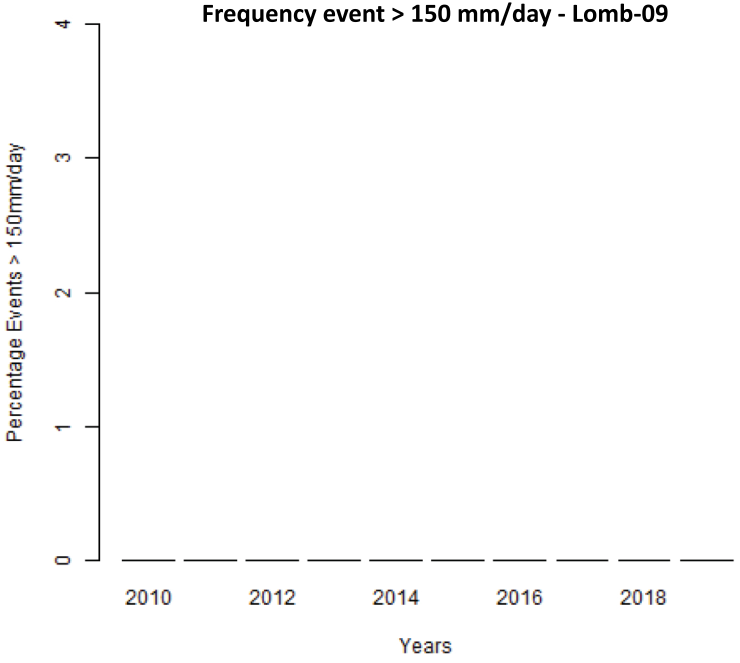
Frequency event > 150 mm/day - Lomb-09
Percentage Events > 150mm/day
0
1
2
3
4
2010
2012
2014
2016
2018
Years
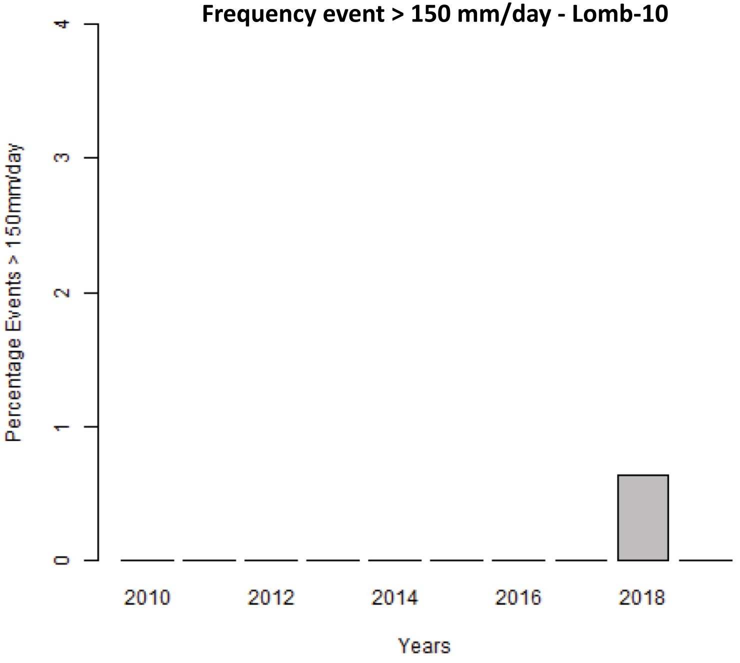
Frequency event > 150 mm/day - Lomb-10
Percentage Events > 150mm/day
0
1
2
3
4
2010
2012
2014
2016
2018
Years
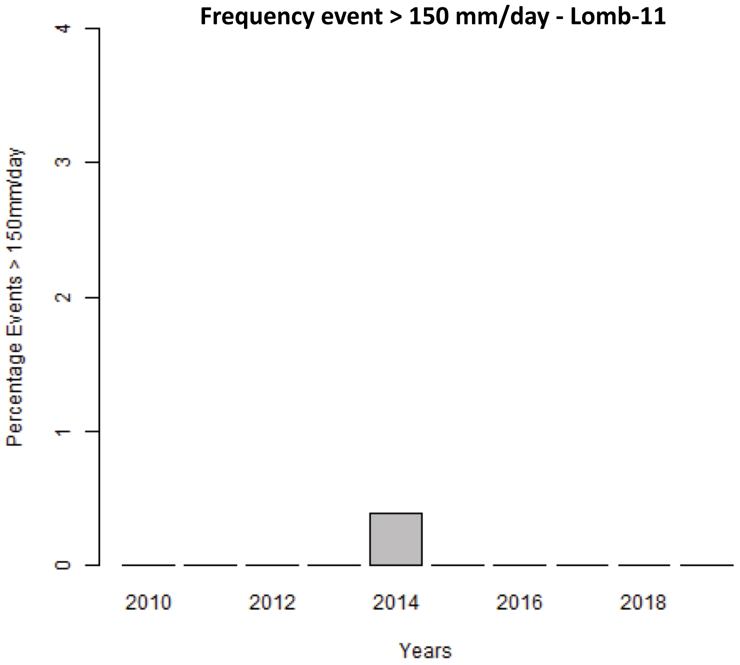
Frequency event > 150 mm/day - Lomb-11
Percentage Events > 150mm/day
0
1
2
3
4
2010
2012
2014
2016
2018
Years
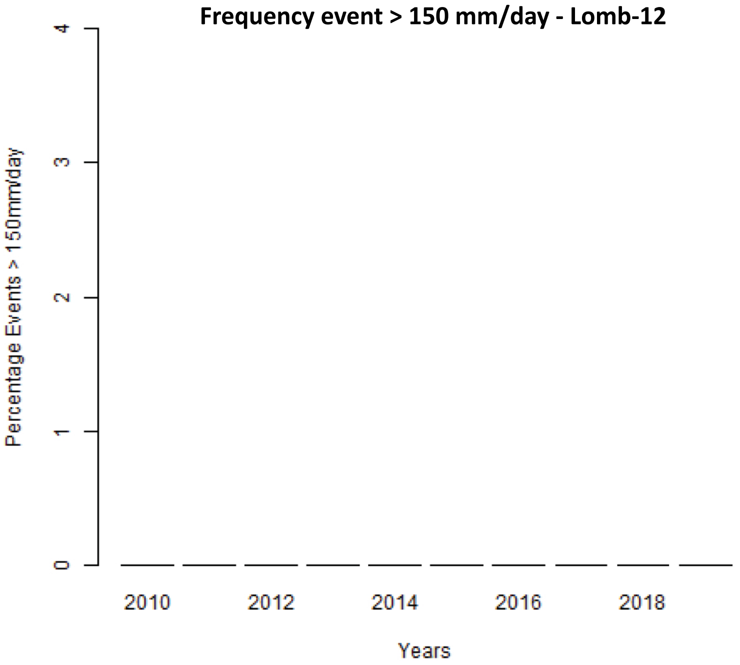
Frequency event > 150 mm/day - Lomb-12
Percentage Events > 150mm/day
0
1
2
3
4
2010
2012
2014
2016
2018
Years
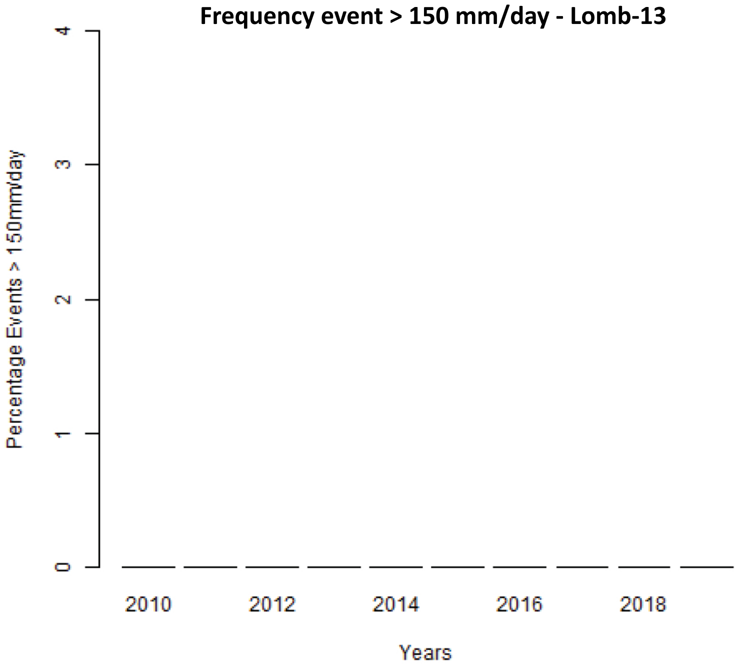
Frequency event > 150 mm/day - Lomb-13
Percentage Events > 150mm/day
0
1
2
3
4
2010
2012
2014
2016
2018
Years
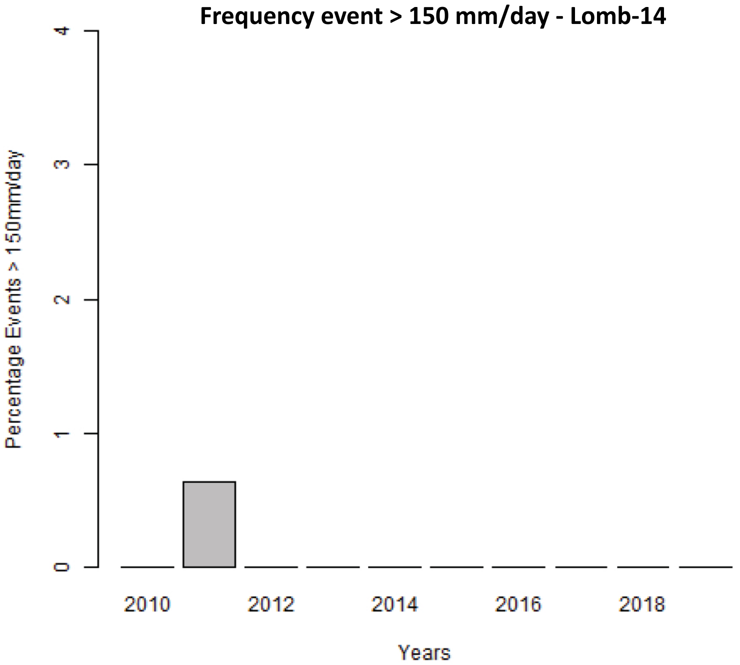
Frequency event > 150 mm/day - Lomb-14
Percentage Events > 150mm/day
0
1
2
3
4
2010
2012
2014
2016
2018
Years

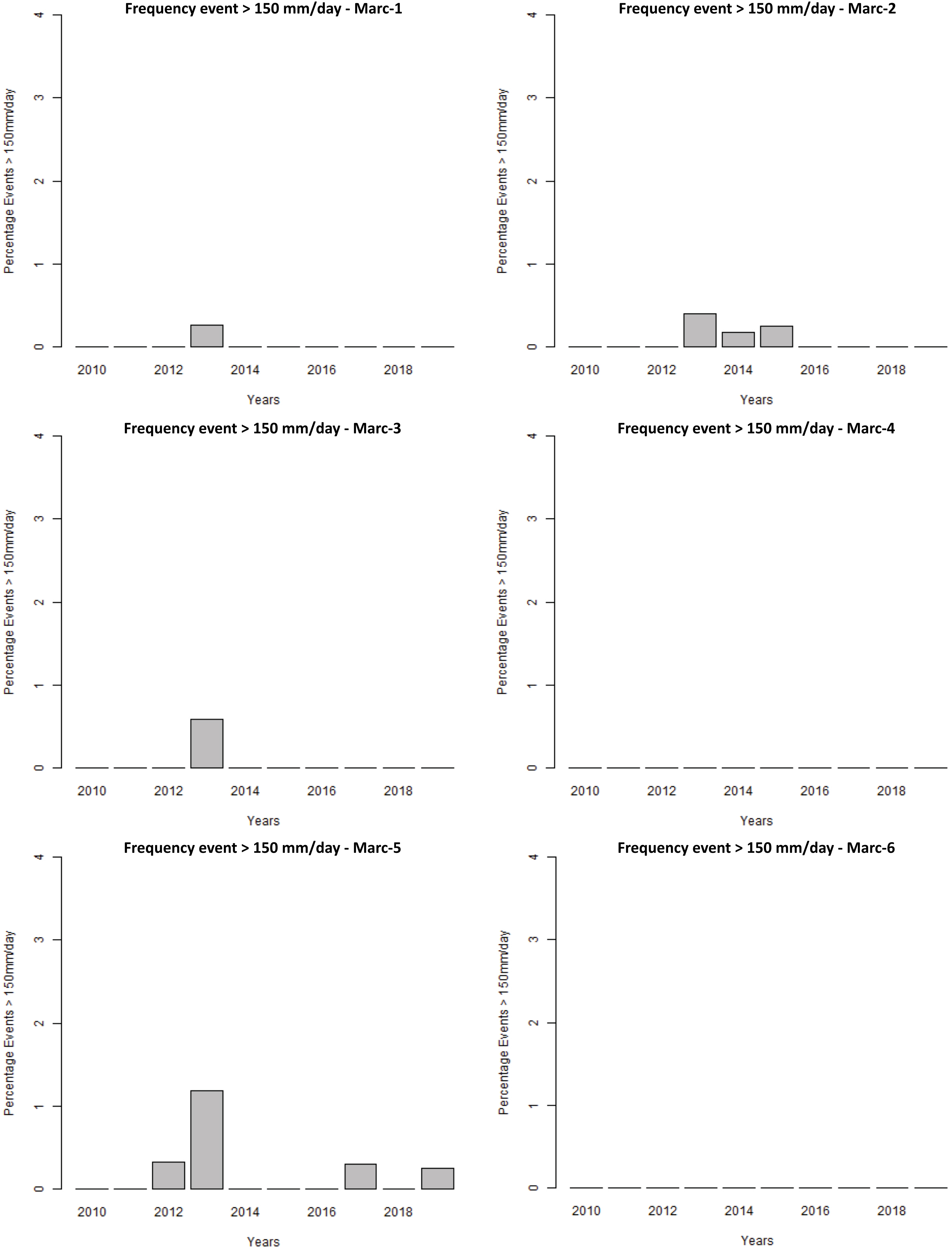

Frequency event > 150 mm/day - Marc-1
Frequency event > 150 mm/day - Marc-2
Frequency event > 150 mm/day - Marc-3
Frequency event > 150 mm/day - Marc-4
Frequency event > 150 mm/day - Marc-5
Frequency event > 150 mm/day - Marc-6
Percentage Events > 150mm/day
Years
0
1
2
3
4
2010
2012
2014
2016
2018

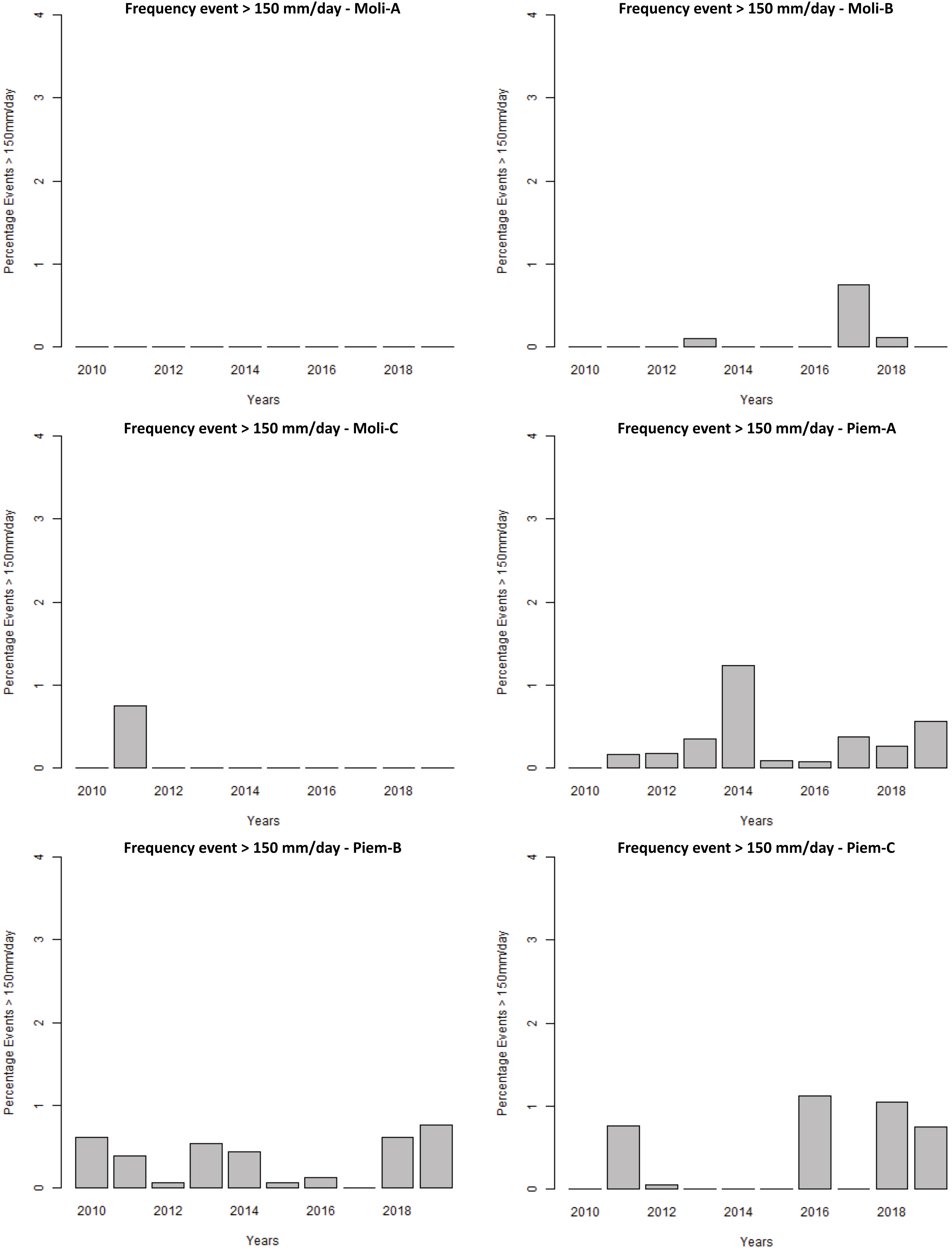

Frequency event > 150 mm/day - Moli-A
Frequency event > 150 mm/day - Moli-B
Frequency event > 150 mm/day - Moli-C
Frequency event > 150 mm/day - Piem-A
Frequency event > 150 mm/day - Piem-B
Frequency event > 150 mm/day - Piem-C
Percentage Events > 150mm/day
Years
0
1
2
3
4
2010
2012
2014
2016
2018

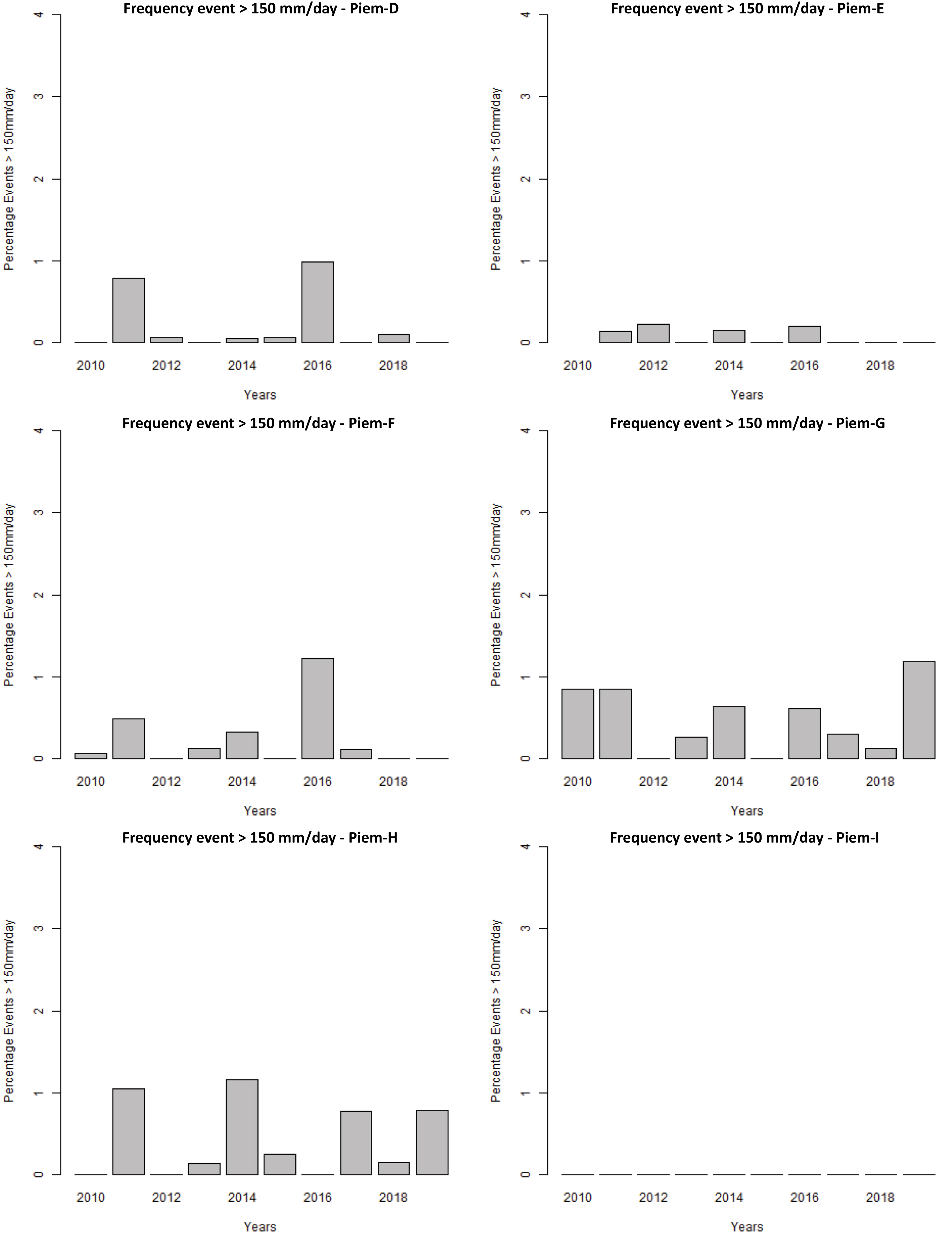
Frequency event > 150 mm/day - Piem-D
Frequency event > 150 mm/day - Piem-E
Frequency event > 150 mm/day - Piem-F
Frequency event > 150 mm/day - Piem-G
Frequency event > 150 mm/day - Piem-H
Frequency event > 150 mm/day - Piem-I
Percentage Events > 150mm/day
Years
0
1
2
3
4
2010
2012
2014
2016
2018

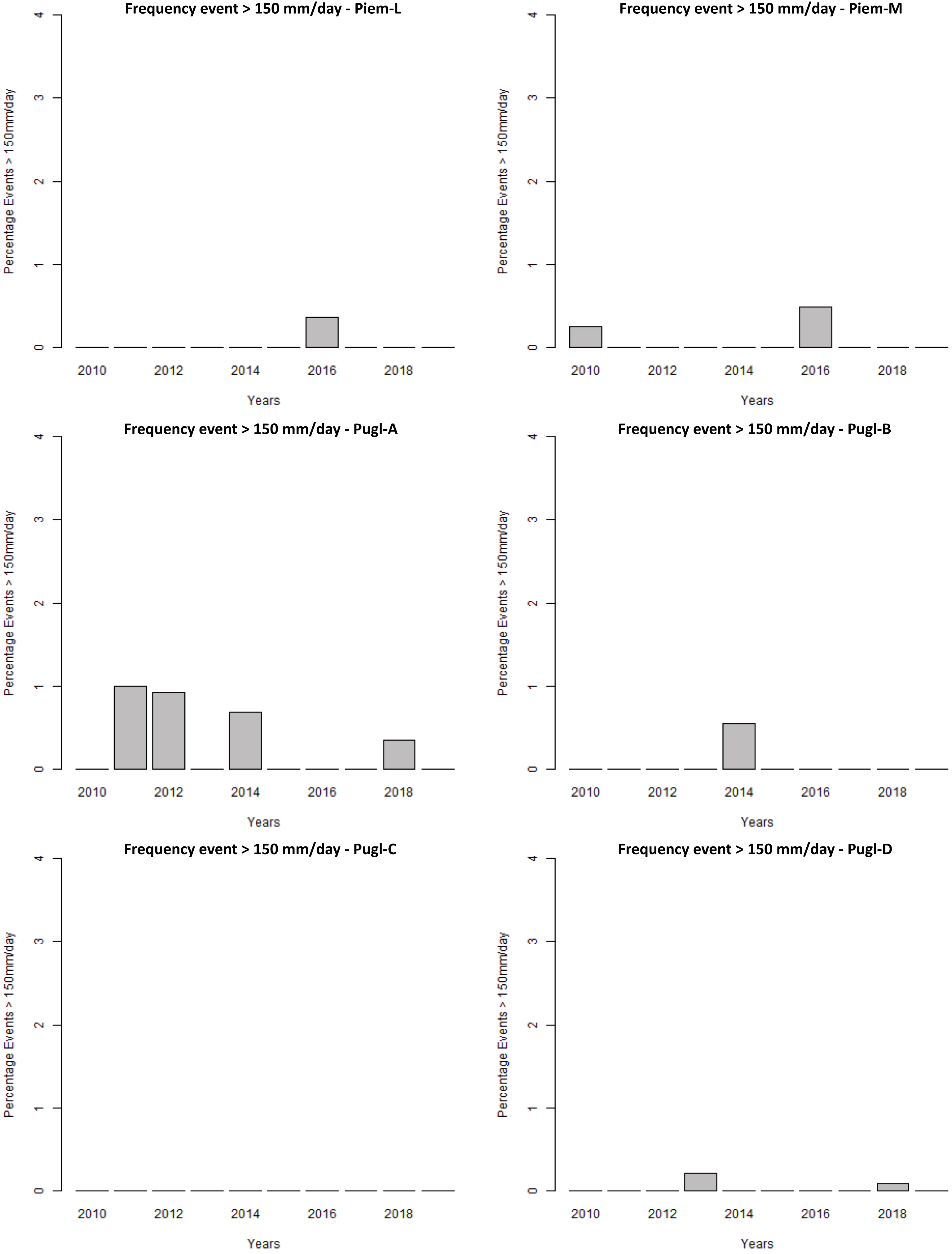
Frequency event > 150 mm/day - Piem-L
Frequency event > 150 mm/day - Piem-M
Frequency event > 150 mm/day - Pugl-A
Frequency event > 150 mm/day - Pugl-B
Frequency event > 150 mm/day - Pugl-C
Frequency event > 150 mm/day - Pugl-D
Percentage Events > 150mm/day
Years
0
1
2
3
4
2010
2012
2014
2016
2018

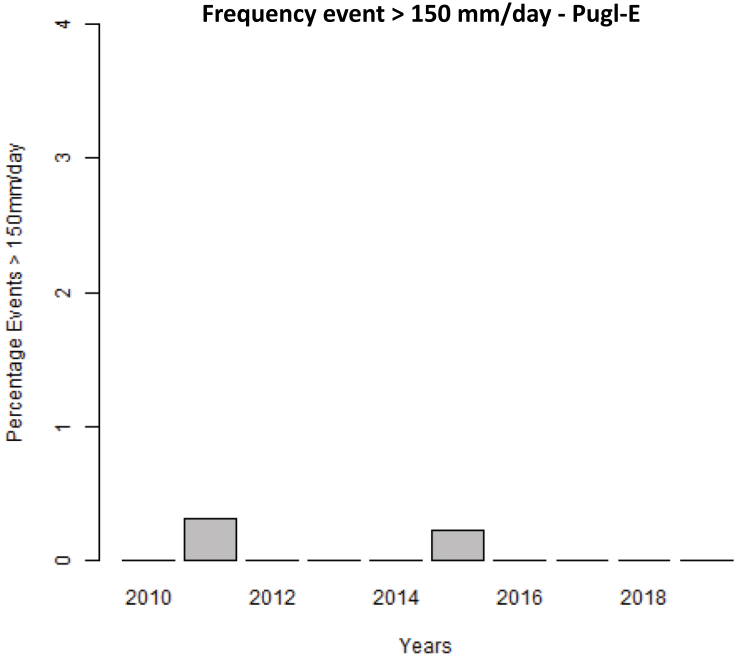
Frequency event > 150 mm/day - Pugl-E
Percentage Events > 150mm/day
0
1
2
3
4
2010
2012
2014
2016
2018
Years

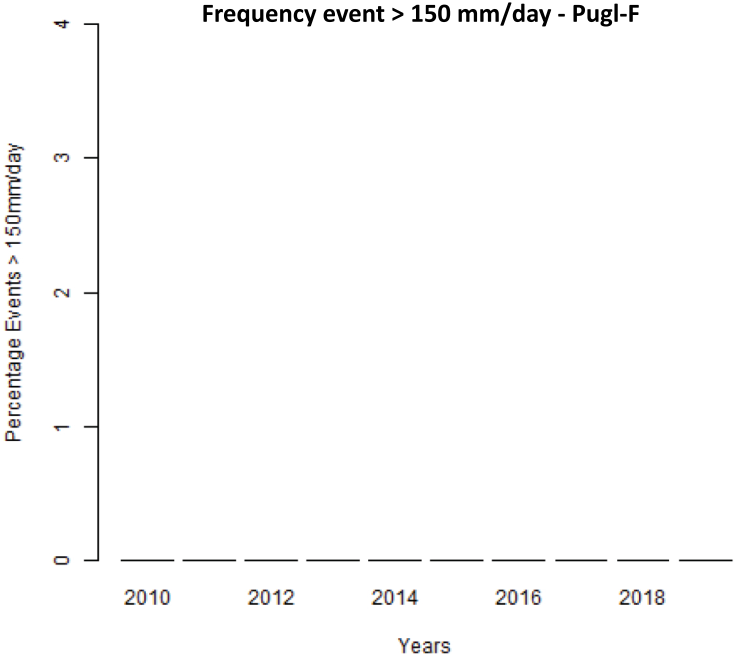
Frequency event > 150 mm/day - Pugl-F
Percentage Events > 150mm/day
0
1
2
3
4
2010
2012
2014
2016
2018
Years

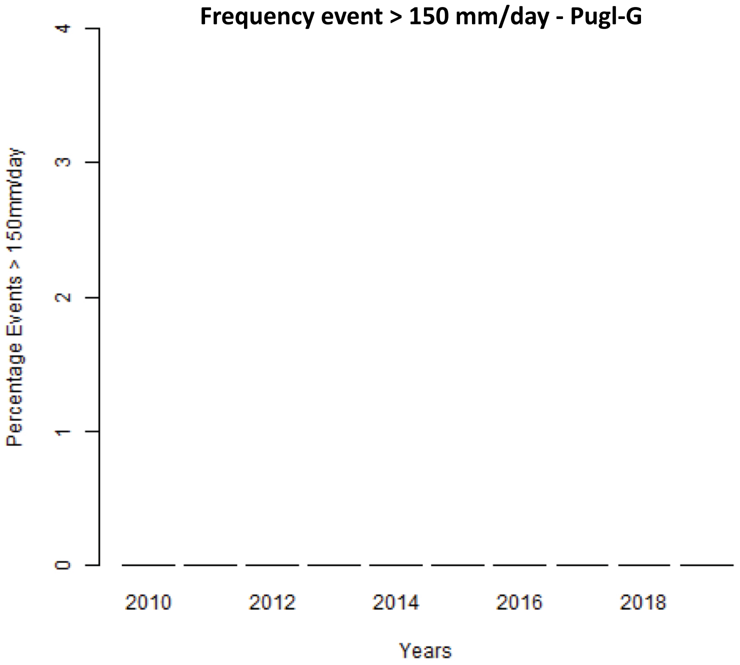
Frequency event > 150 mm/day - Pugl-G
Percentage Events > 150mm/day
0
1
2
3
4
2010
2012
2014
2016
2018
Years

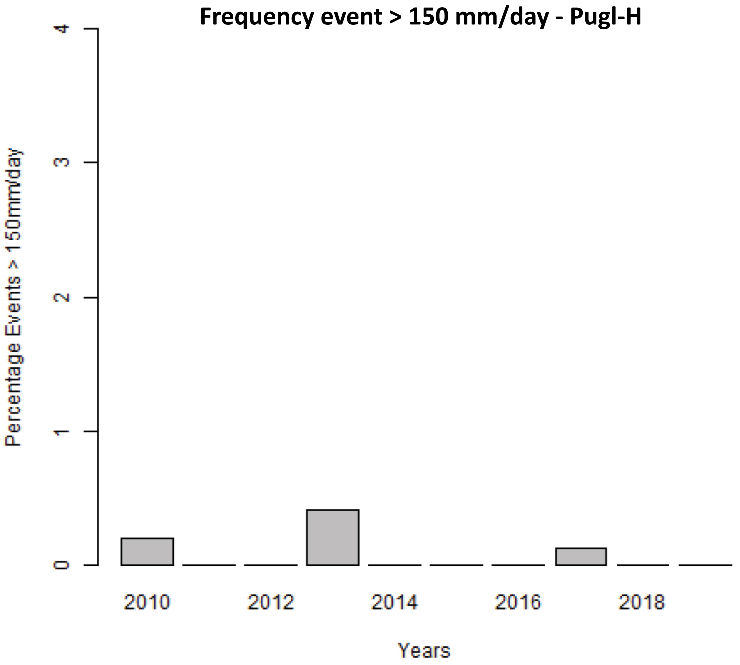
Frequency event > 150 mm/day - Pugl-H
Percentage Events > 150mm/day
0
1
2
3
4
2010
2012
2014
2016
2018
Years

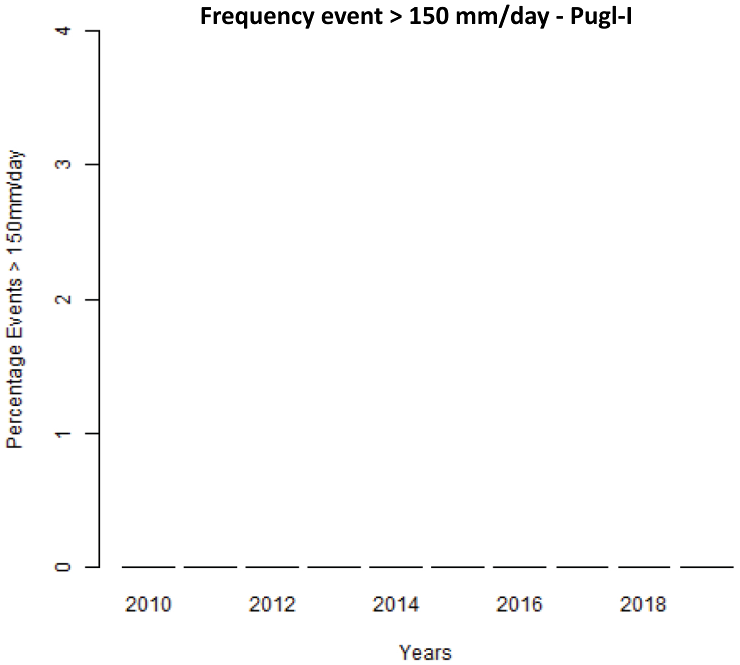
Frequency event > 150 mm/day - Pugl-I
Percentage Events > 150mm/day
0
1
2
3
4
2010
2012
2014
2016
2018
Years

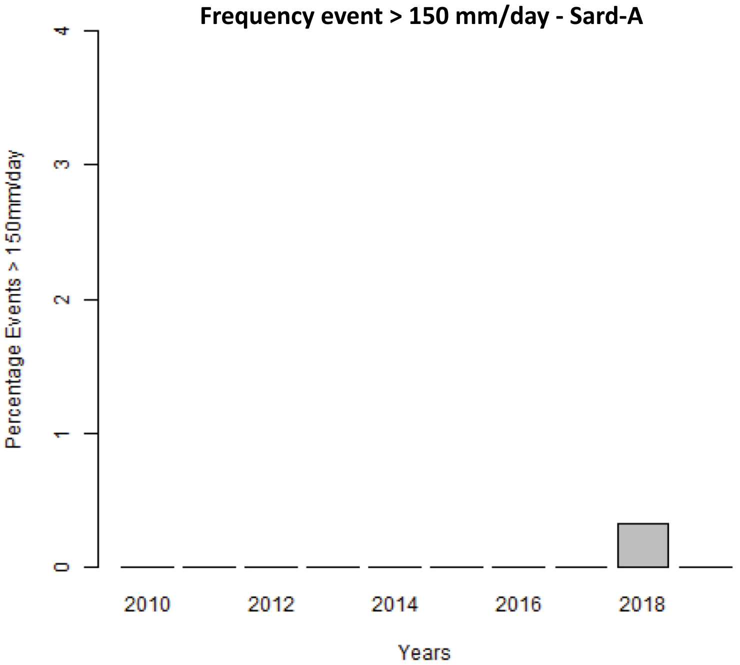
Frequency event > 150 mm/day - Sard-A
Percentage Events > 150mm/day
0
1
2
3
4
2010
2012
2014
2016
2018
Years

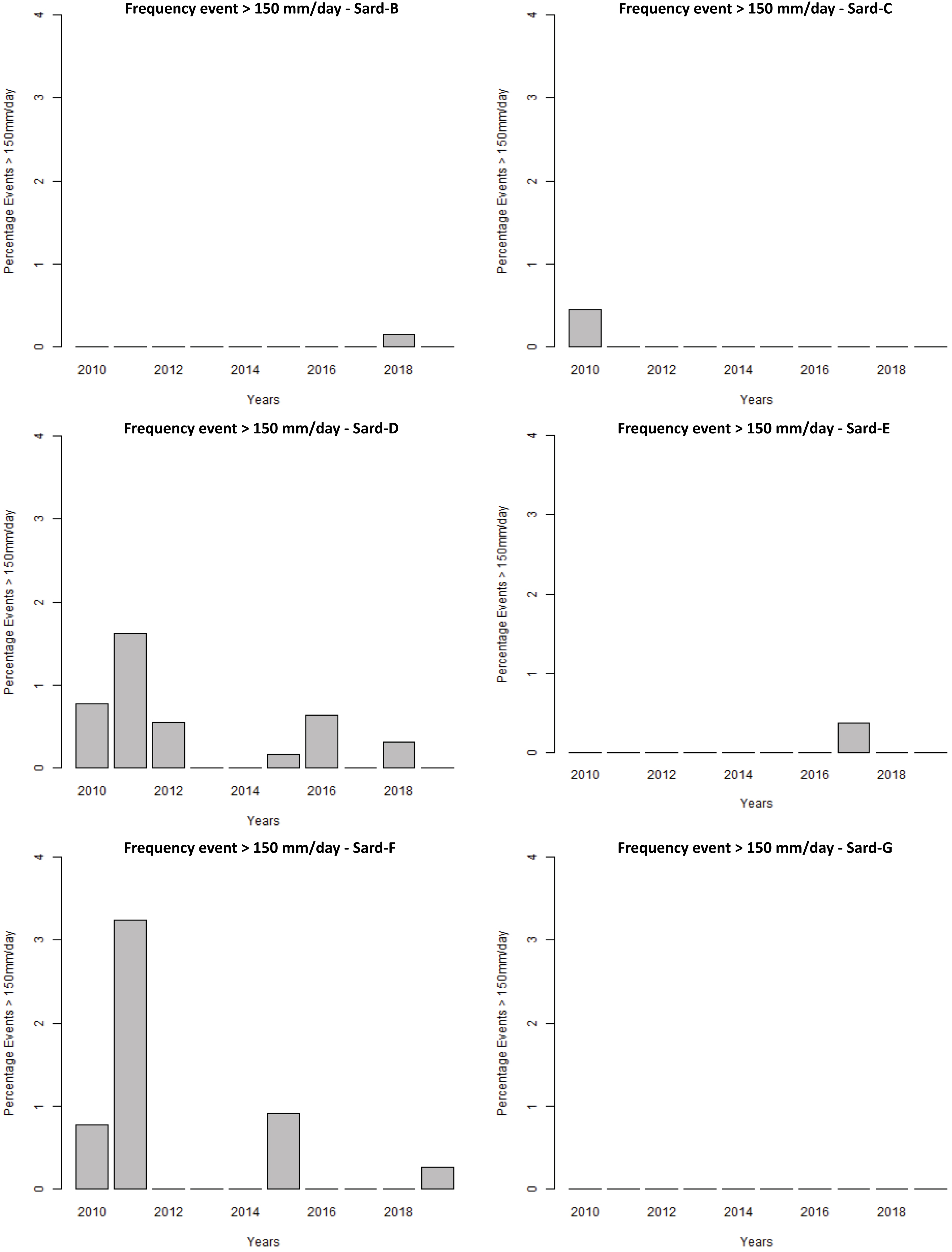
Frequency event > 150 mm/day - Sard-B
Frequency event > 150 mm/day - Sard-C
Frequency event > 150 mm/day - Sard-D
Frequency event > 150 mm/day - Sard-E
Frequency event > 150 mm/day - Sard-F
Frequency event > 150 mm/day - Sard-G
Percentage Events > 150mm/day
Years
0
1
2
3
4
2010
2012
2014
2016
2018

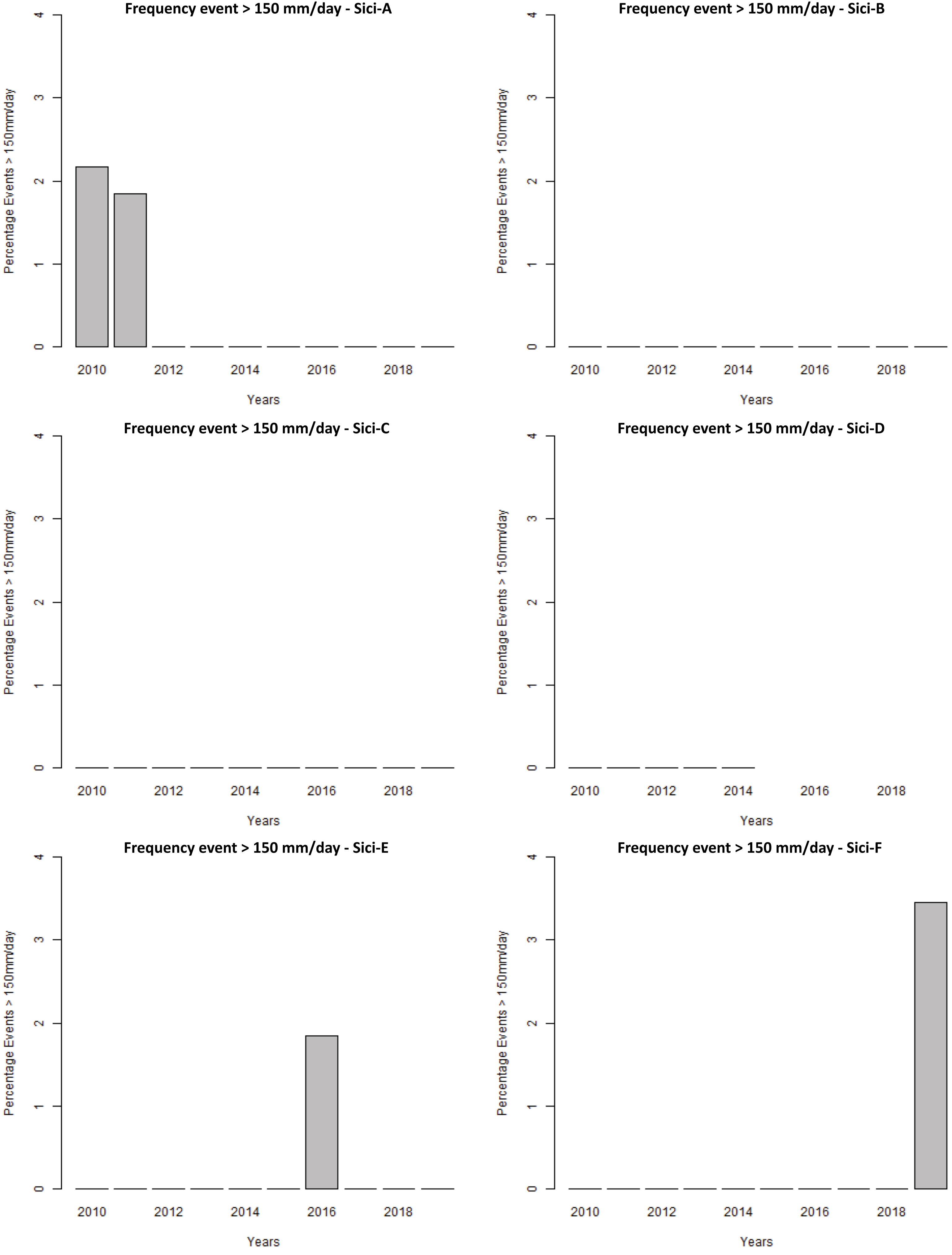
Frequency event > 150 mm/day - Sici-A
Frequency event > 150 mm/day - Sici-B
Frequency event > 150 mm/day - Sici-C
Frequency event > 150 mm/day - Sici-D
Frequency event > 150 mm/day - Sici-E
Frequency event > 150 mm/day - Sici-F
Percentage Events > 150mm/day
Years
2010
2012
2014
2016
2018
0
1
2
3
4

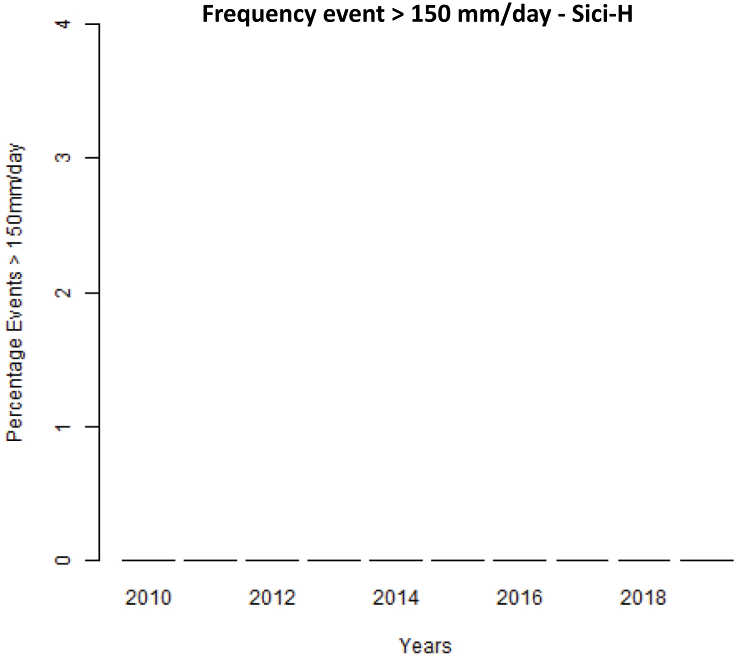
Frequency event > 150 mm/day - Sici-H
Percentage Events > 150mm/day
0
1
2
3
4
2010
2012
2014
2016
2018
Years

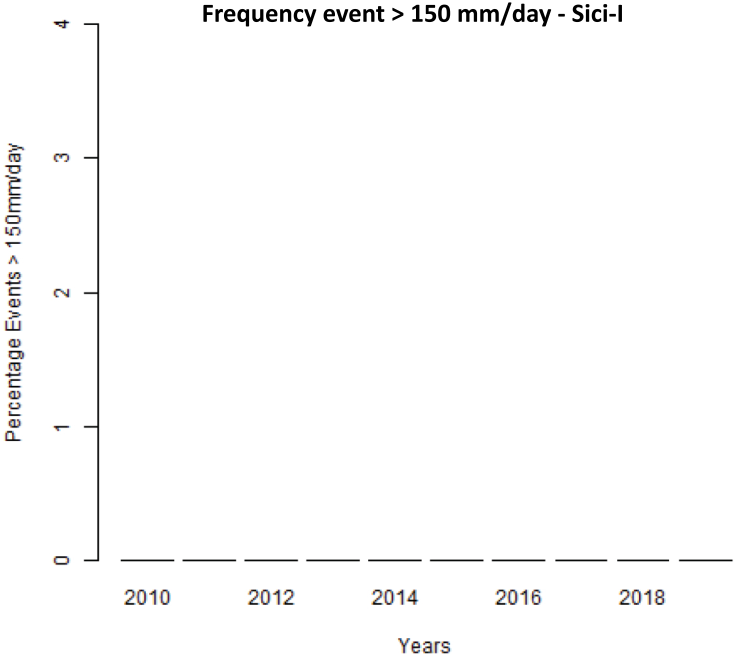
Frequency event > 150 mm/day - Sici-I
Percentage Events > 150mm/day
0
1
2
3
4
2010
2012
2014
2016
2018
Years

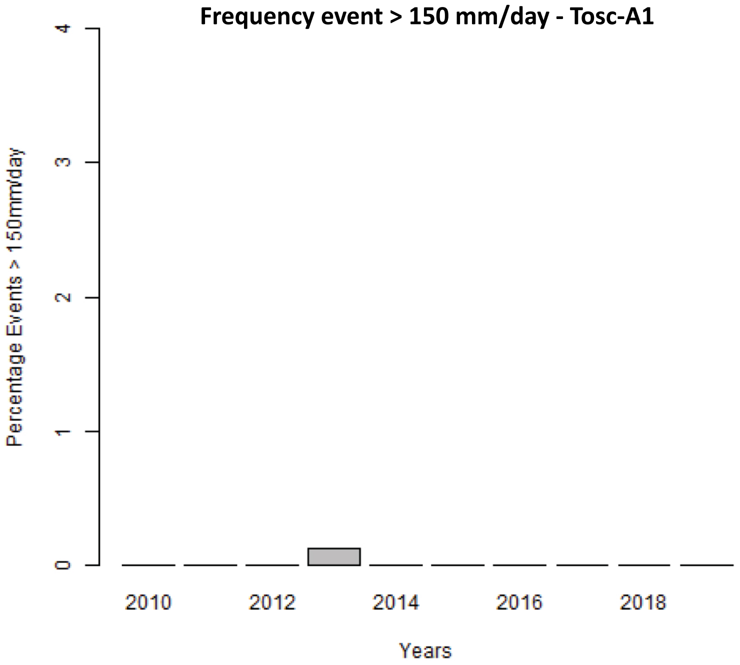
Frequency event > 150 mm/day - Tosc-A1
Percentage Events > 150mm/day
0
1
2
3
4
2010
2012
2014
2016
2018
Years

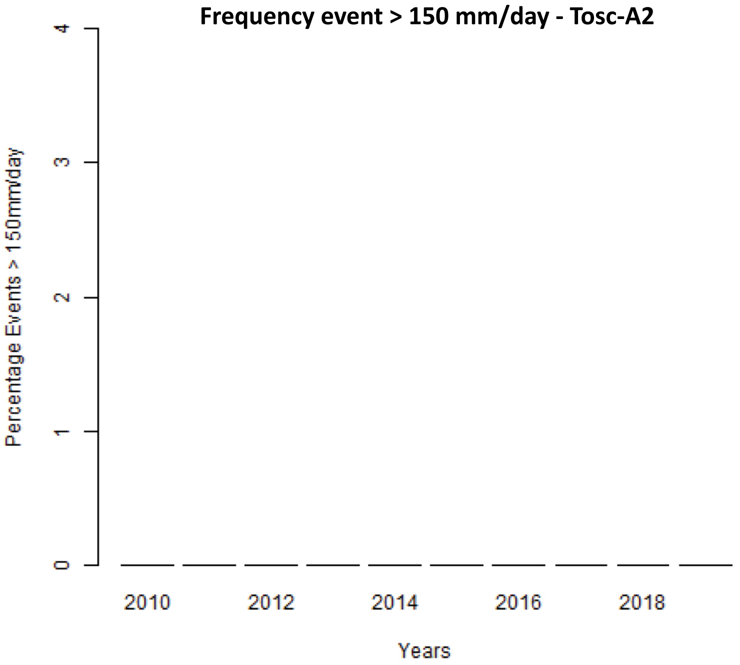
Frequency event > 150 mm/day - Tosc-A2
Percentage Events > 150mm/day
0
1
2
3
4
2010
2012
2014
2016
2018
Years

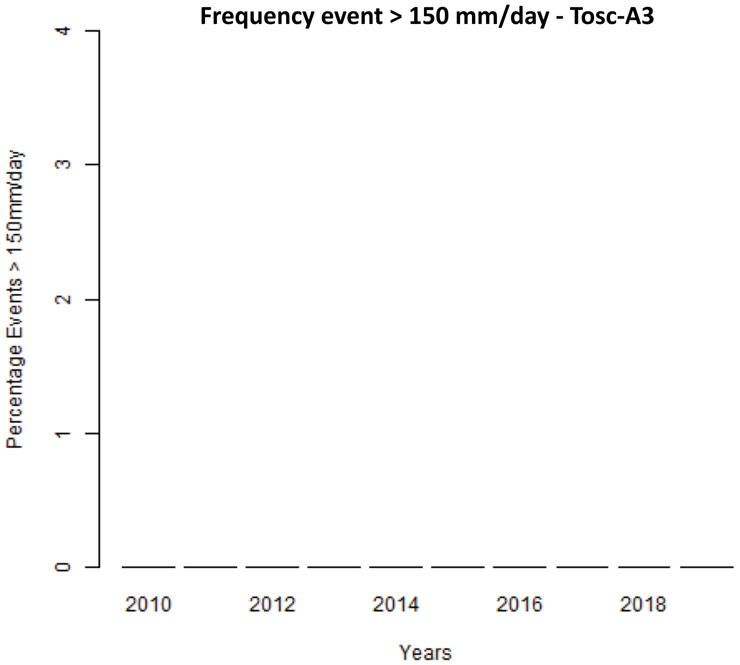
Frequency event > 150 mm/day - Tosc-A3
Percentage Events > 150mm/day
0
1
2
3
4
2010
2012
2014
2016
2018
Years

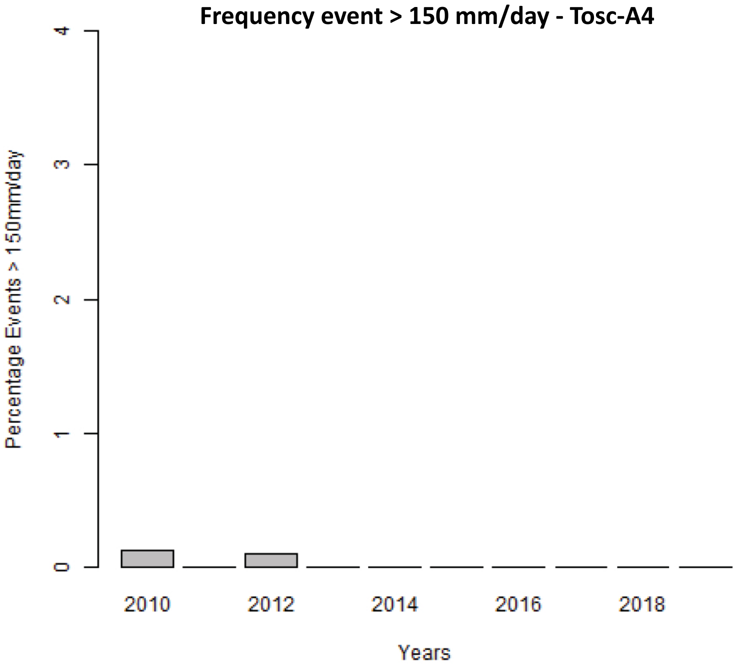
Frequency event > 150 mm/day - Tosc-A4
Percentage Events > 150mm/day
0
1
2
3
4
2010
2012
2014
2016
2018
Years

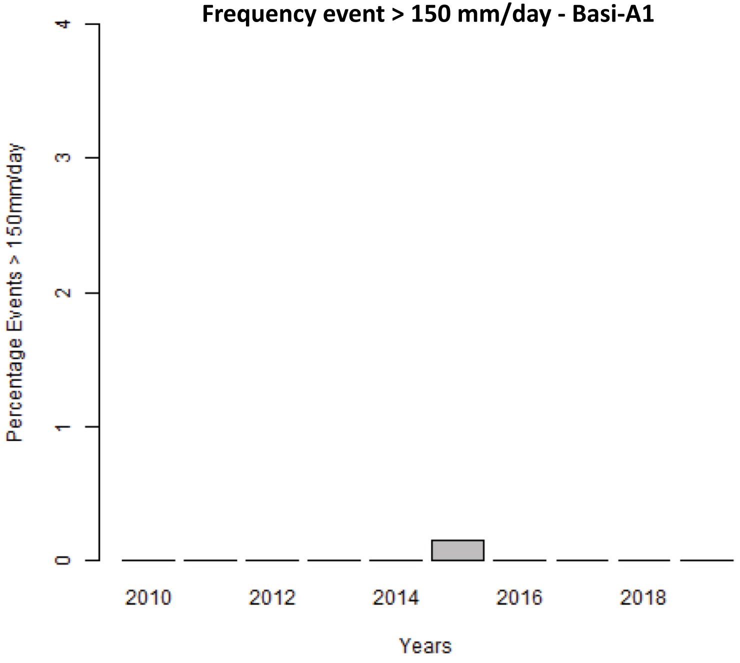
Frequency event > 150 mm/day - Basi-A1
Percentage Events > 150mm/day
0
1
2
3
4
2010
2012
2014
2016
2018
Years

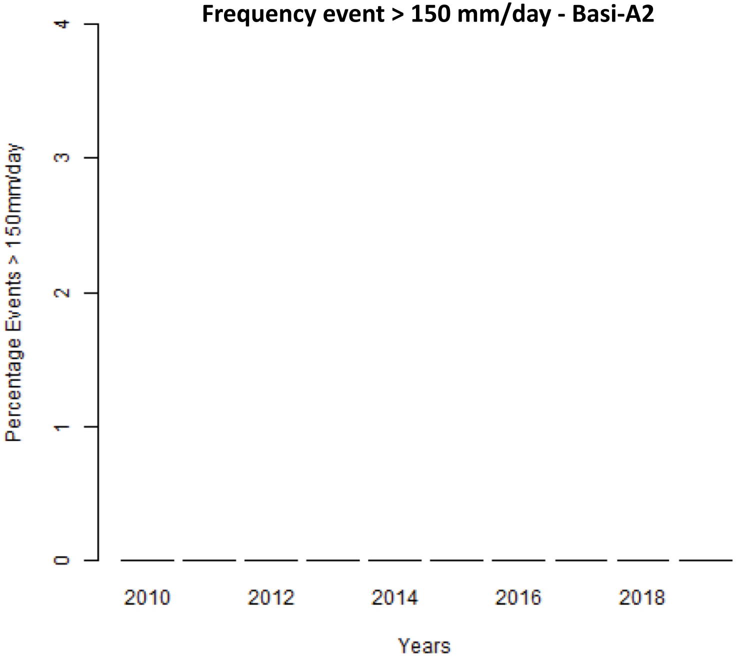
Frequency event > 150 mm/day - Basi-A2
Percentage Events > 150mm/day
0
1
2
3
4
2010
2012
2014
2016
2018
Years

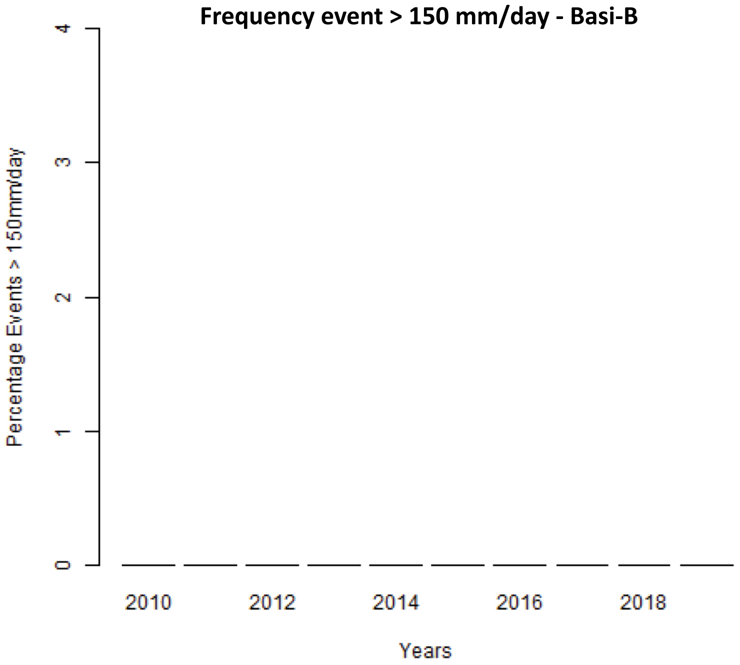
Frequency event > 150 mm/day - Basi-B
Percentage Events > 150mm/day
0
1
2
3
4
2010
2012
2014
2016
2018
Years

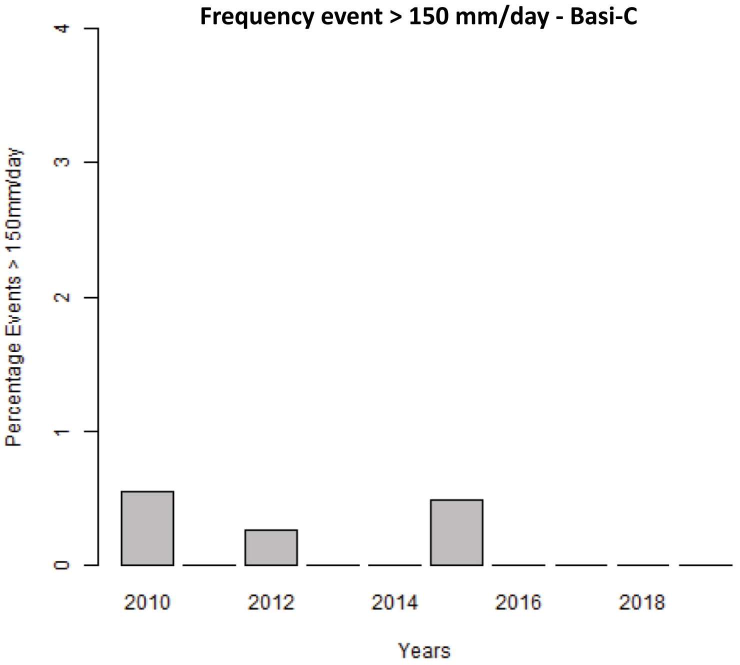
Frequency event > 150 mm/day - Basi-C
Percentage Events > 150mm/day
0
1
2
3
4
2010
2012
2014
2016
2018
Years

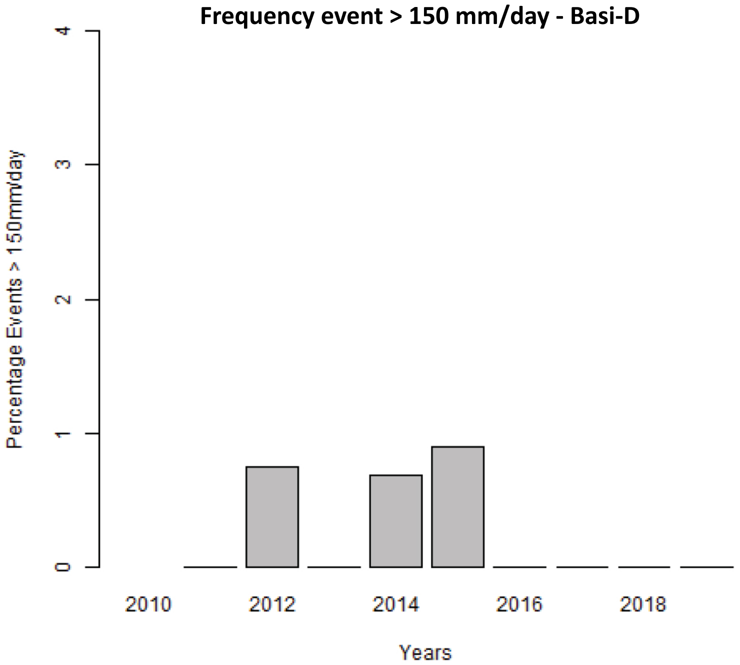
Frequency event > 150 mm/day - Basi-D
Percentage Events > 150mm/day
0
1
2
3
4
2010
2012
2014
2016
2018
Years

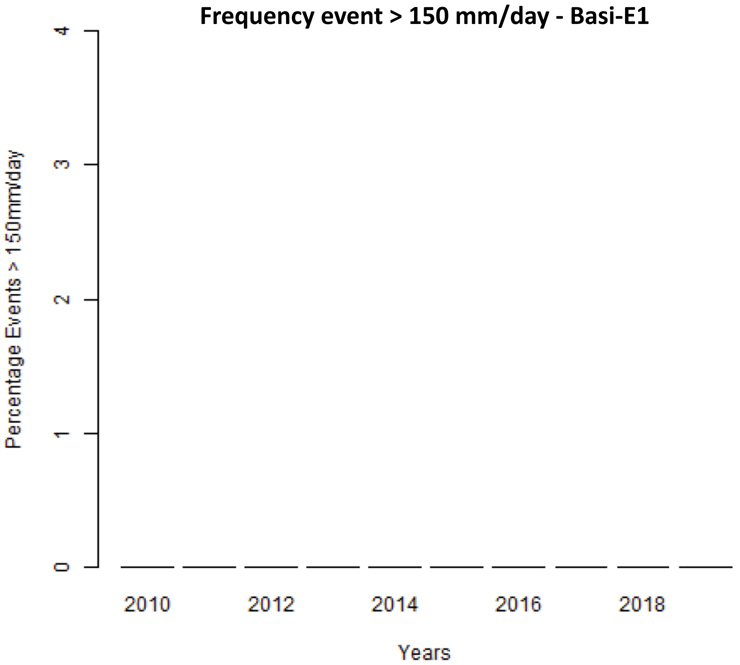
Frequency event > 150 mm/day - Basi-E1
Percentage Events > 150mm/day
0
1
2
3
4
2010
2012
2014
2016
2018
Years

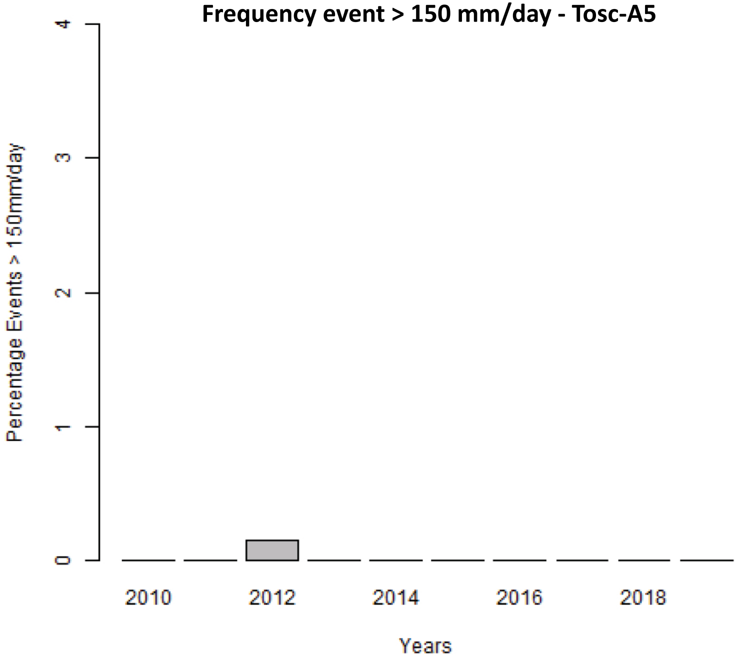
Frequency event > 150 mm/day - Tosc-A5
Percentage Events > 150mm/day
0
1
2
3
4
2010
2012
2014
2016
2018
Years

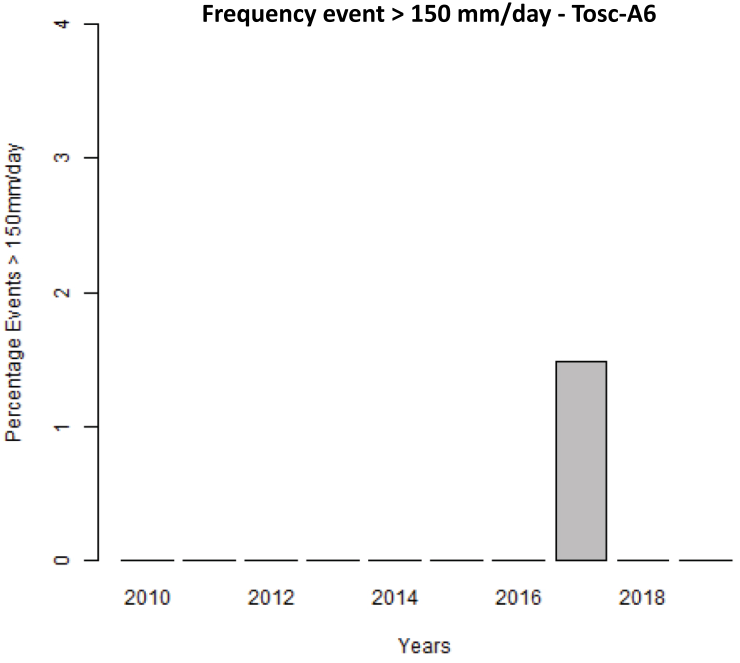
Frequency event > 150 mm/day - Tosc-A6
Percentage Events > 150mm/day
0
1
2
3
4
2010
2012
2014
2016
2018
Years

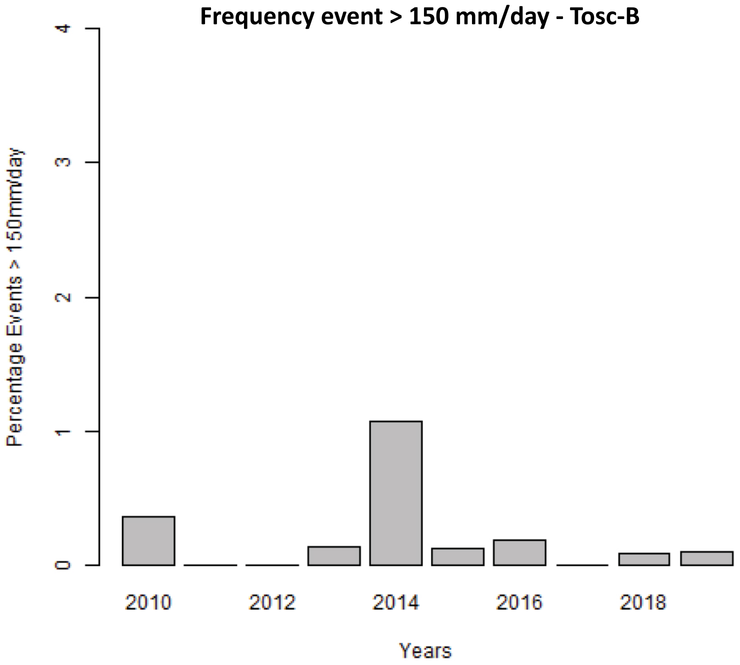
Frequency event > 150 mm/day - Tosc-B
Percentage Events > 150mm/day
0
1
2
3
4
2010
2012
2014
2016
2018
Years

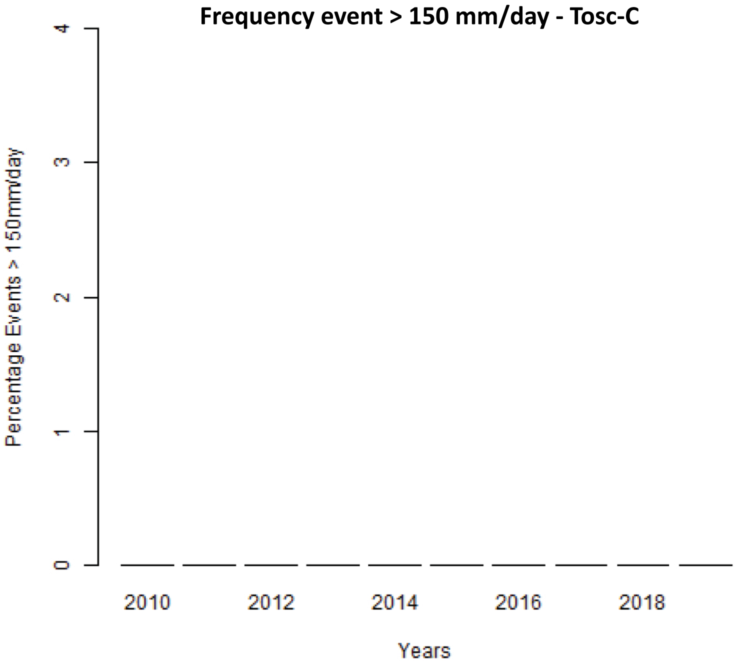
Frequency event > 150 mm/day - Tosc-C
Percentage Events > 150mm/day
0
1
2
3
4
2010
2012
2014
2016
2018
Years

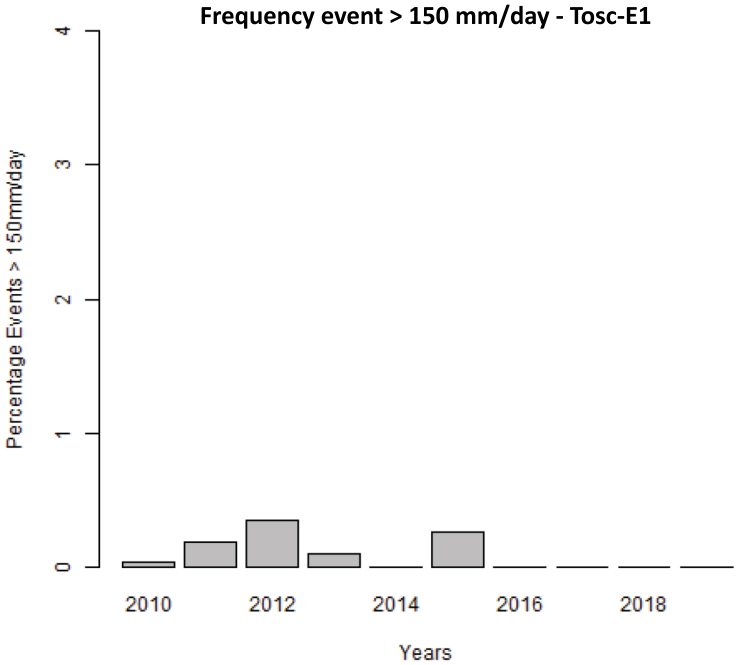
Frequency event > 150 mm/day - Tosc-E1
Percentage Events > 150mm/day
0
1
2
3
4
2010
2012
2014
2016
2018
Years

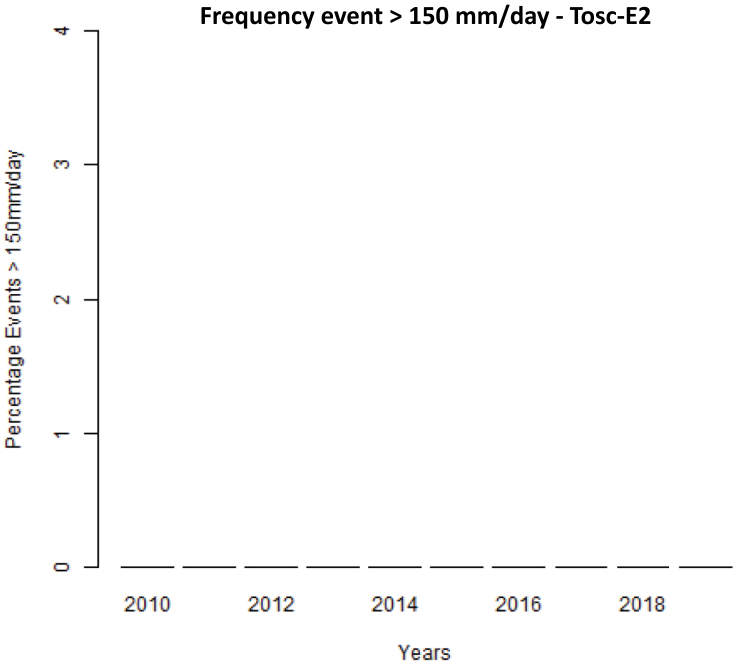
Frequency event > 150 mm/day - Tosc-E2
Percentage Events > 150mm/day
0
1
2
3
4
2010
2012
2014
2016
2018
Years

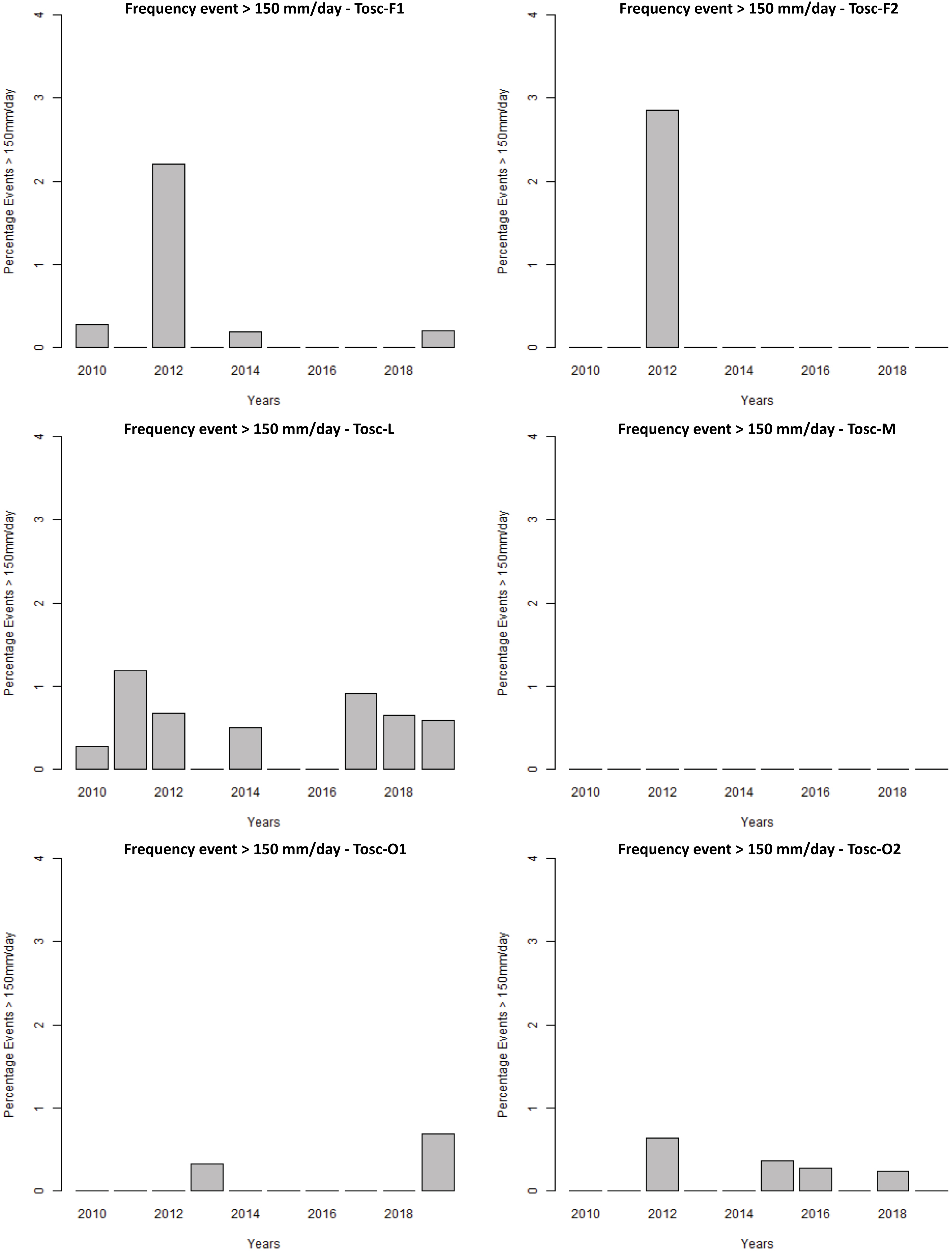
Frequency event > 150 mm/day - Tosc-F1
Frequency event > 150 mm/day - Tosc-F2
Frequency event > 150 mm/day - Tosc-L
Frequency event > 150 mm/day - Tosc-M
Frequency event > 150 mm/day - Tosc-O1
Frequency event > 150 mm/day - Tosc-O2
Percentage Events > 150mm/day
Years
2010
2012
2014
2016
2018
0
1
2
3
4

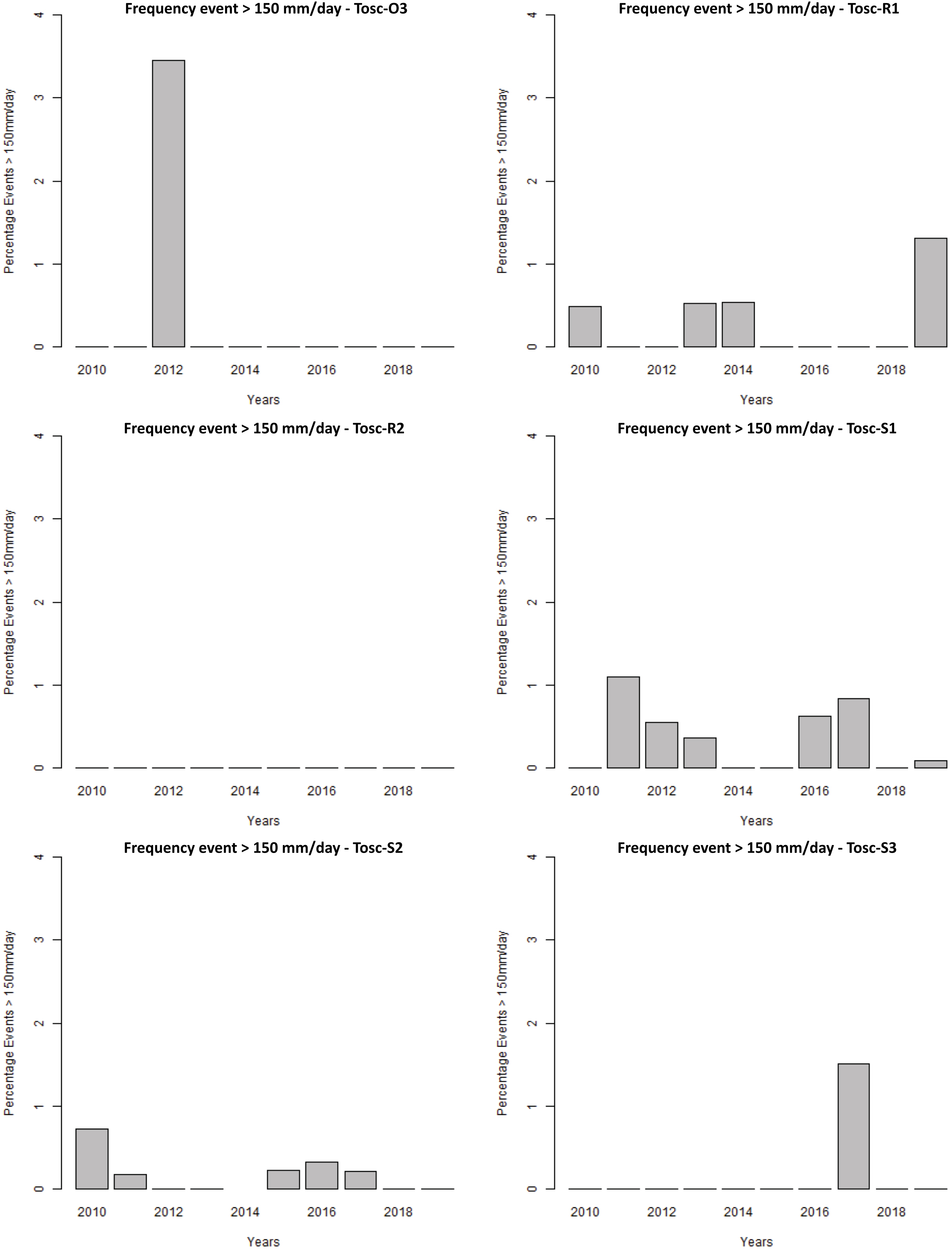
Frequency event > 150 mm/day - Tosc-O3
Frequency event > 150 mm/day - Tosc-R1
Frequency event > 150 mm/day - Tosc-R2
Frequency event > 150 mm/day - Tosc-S1
Frequency event > 150 mm/day - Tosc-S2
Frequency event > 150 mm/day - Tosc-S3
Percentage Events > 150mm/day
0
1
2
3
4
2010
2012
2014
2016
2018
Years

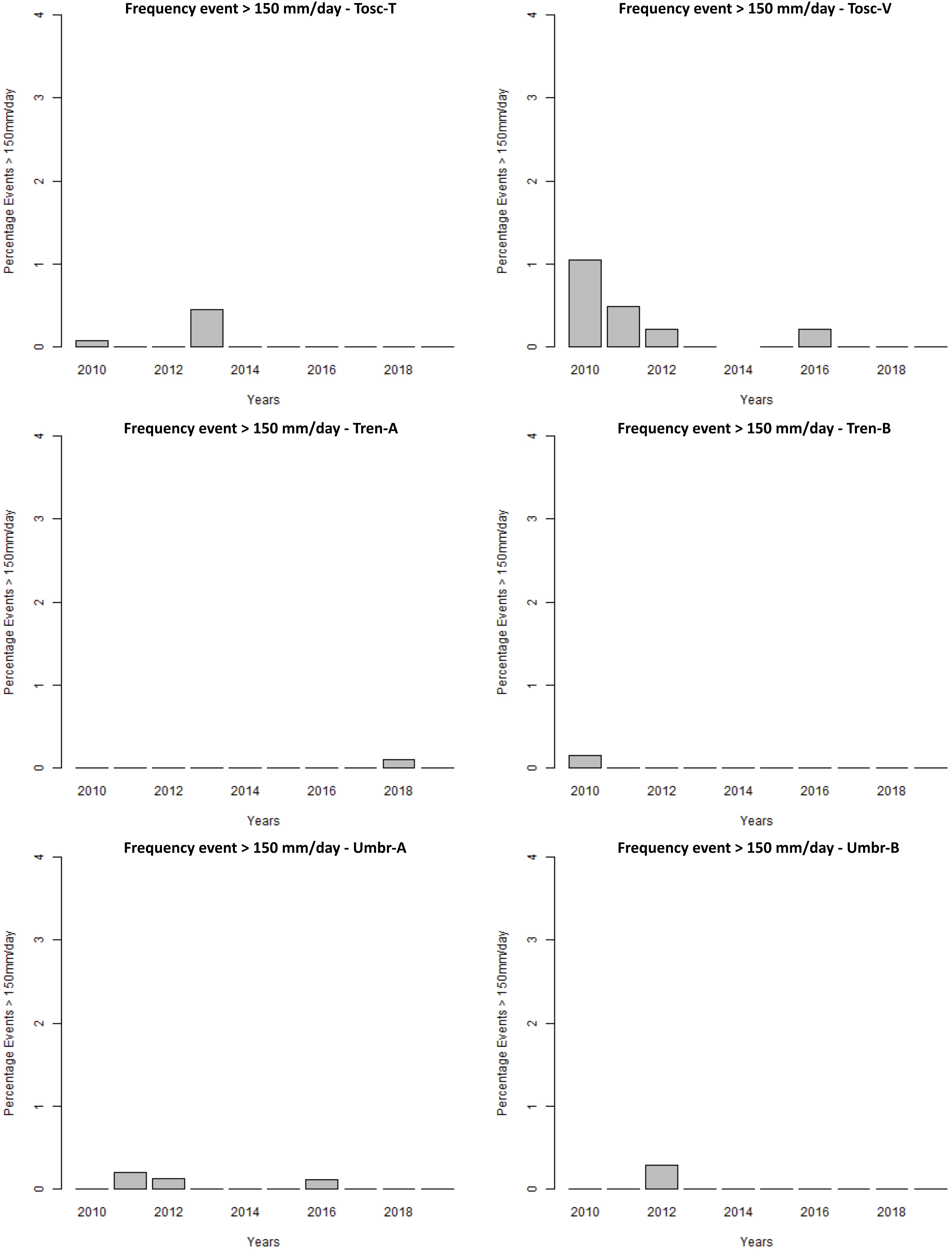
Frequency event > 150 mm/day - Tosc-T
Frequency event > 150 mm/day - Tosc-V
Frequency event > 150 mm/day - Tren-A
Frequency event > 150 mm/day - Tren-B
Frequency event > 150 mm/day - Umbr-A
Frequency event > 150 mm/day - Umbr-B
Percentage Events > 150mm/day
Years
0
1
2
3
4
2010
2012
2014
2016
2018

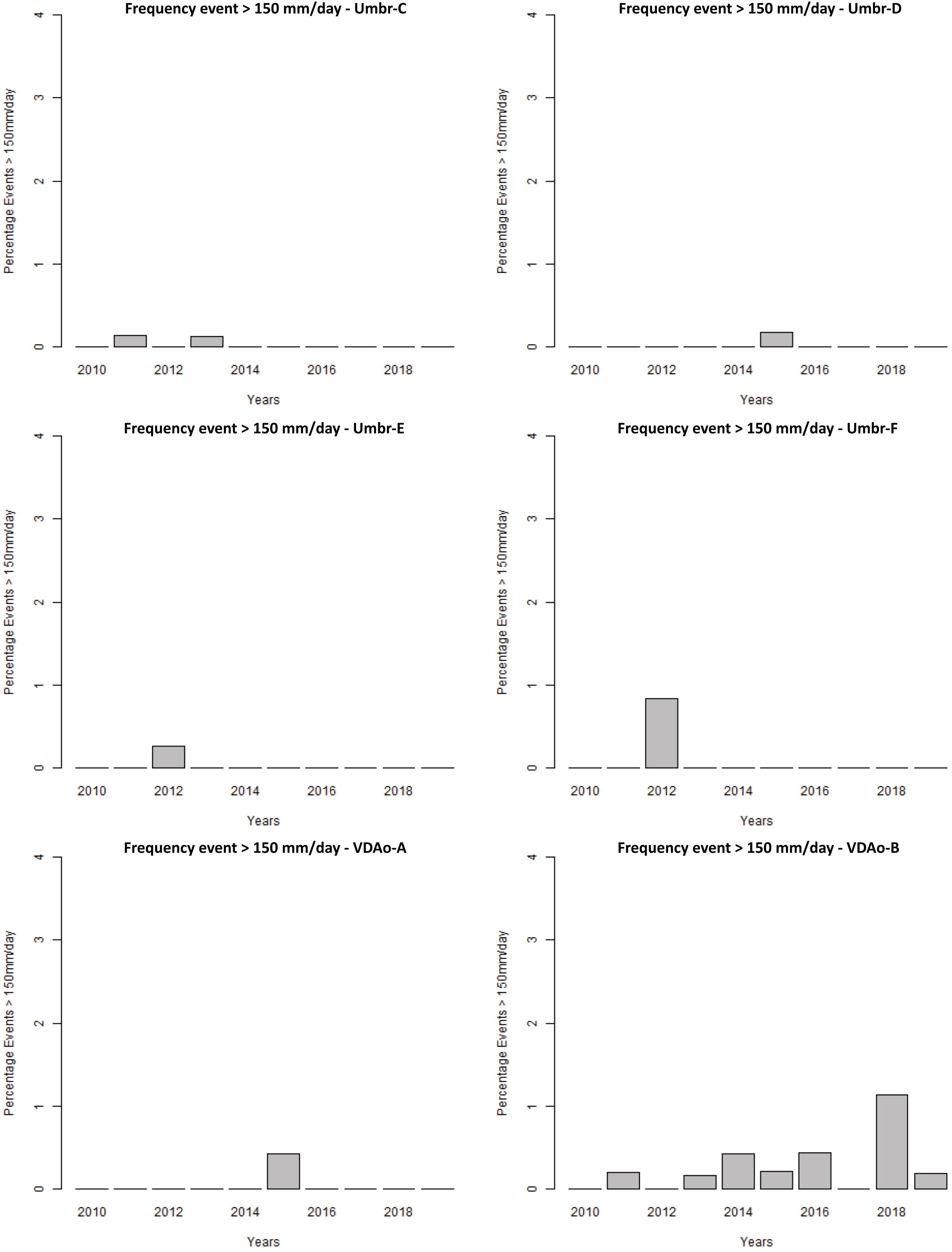
Frequency event > 150 mm/day - Umbr-C
Frequency event > 150 mm/day - Umbr-D
Frequency event > 150 mm/day - Umbr-E
Frequency event > 150 mm/day - Umbr-F
Frequency event > 150 mm/day - VDAo-A
Frequency event > 150 mm/day - VDAo-B
Percentage Events > 150mm/day
Years
0
1
2
3
4
2010
2012
2014
2016
2018

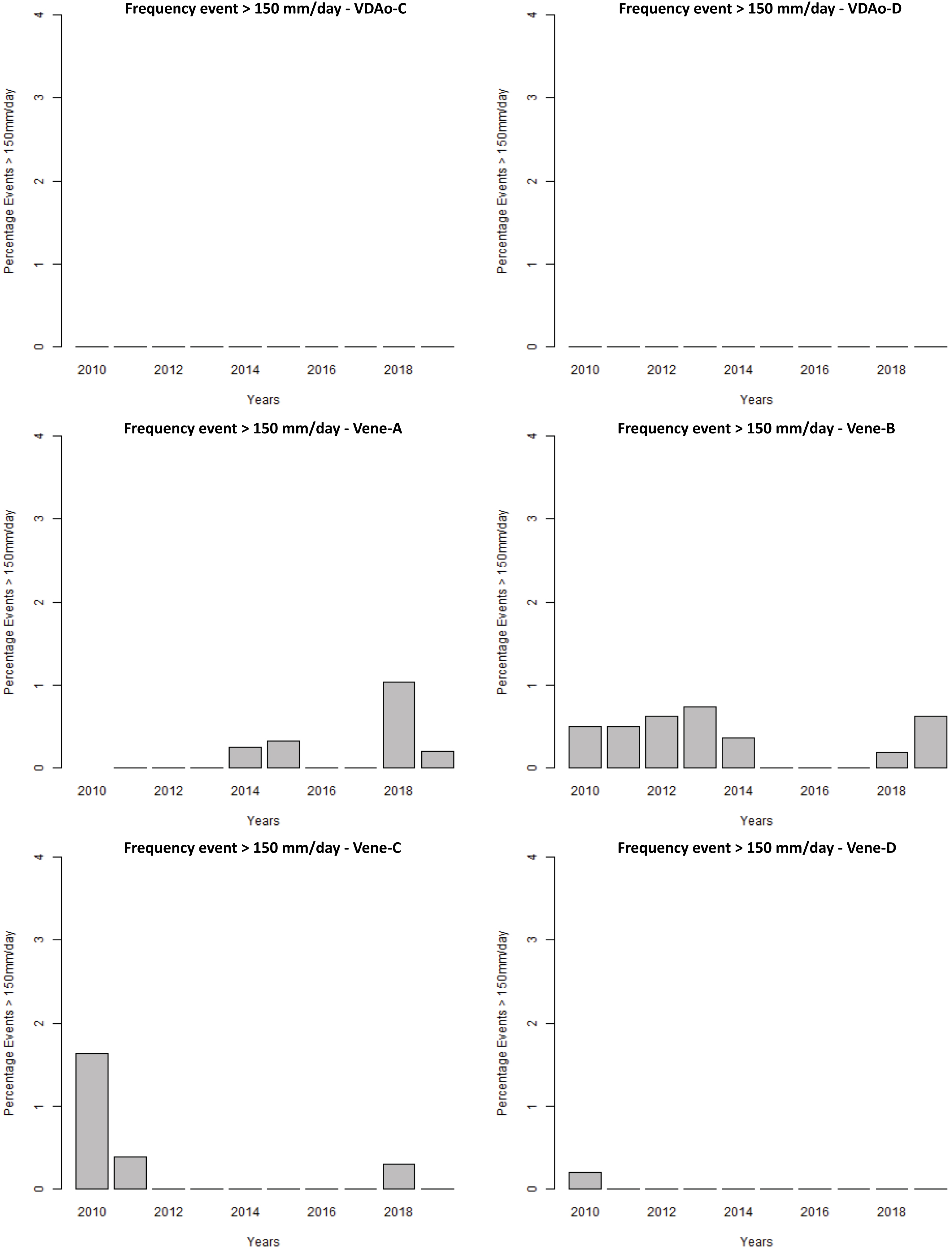
Frequency event > 150 mm/day - VDAo-C
Frequency event > 150 mm/day - VDAo-D
Frequency event > 150 mm/day - Vene-A
Frequency event > 150 mm/day - Vene-B
Frequency event > 150 mm/day - Vene-C
Frequency event > 150 mm/day - Vene-D
Percentage Events > 150mm/day
Years
0
1
2
3
4
2010
2012
2014
2016
2018

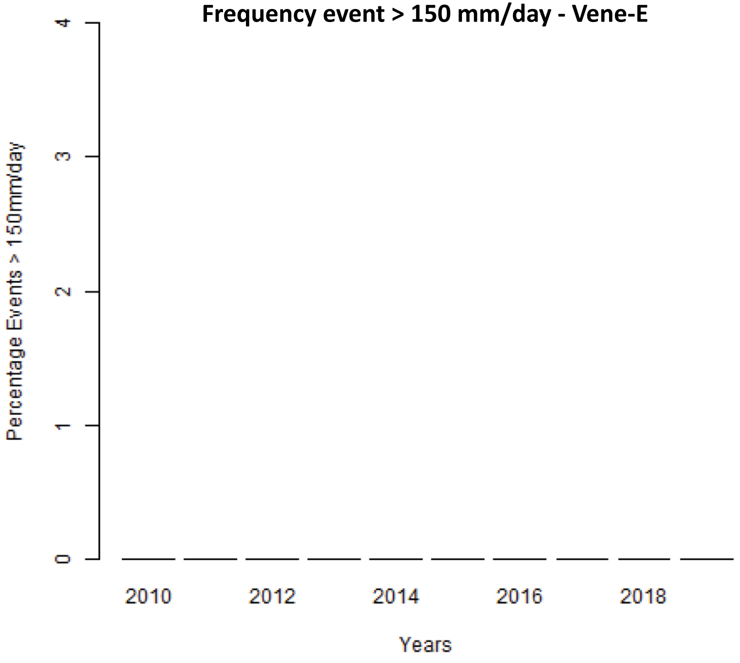
Frequency event > 150 mm/day - Vene-E
Percentage Events > 150mm/day
Years
0
1
2
3
4
2010
2012
2014
2016
2018

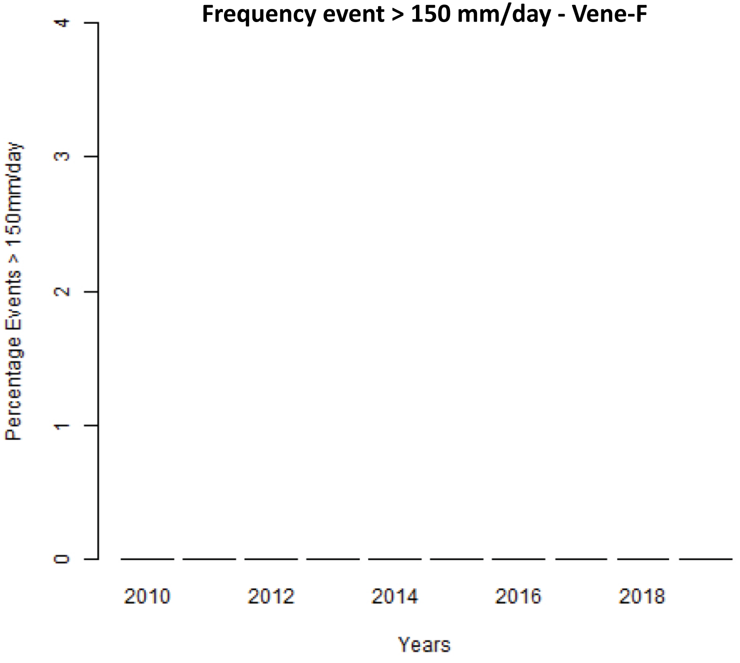
Frequency event > 150 mm/day - Vene-F
Percentage Events > 150mm/day
Years
0
1
2
3
4
2010
2012
2014
2016
2018

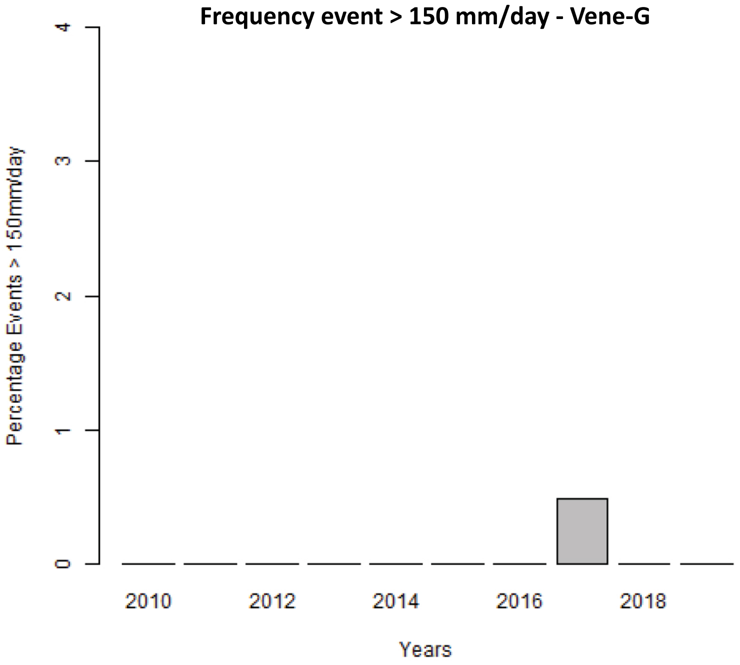
Frequency event > 150 mm/day - Vene-G
Percentage Events > 150mm/day
Years
0
1
2
3
4
2010
2012
2014
2016
2018

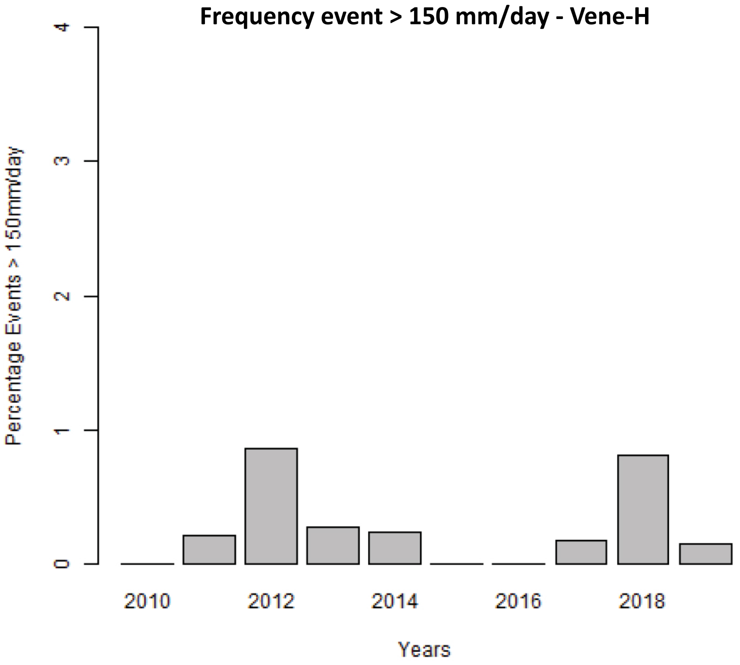
Frequency event > 150 mm/day - Vene-H
Percentage Events > 150mm/day
Years
0
1
2
3
4
2010
2012
2014
2016
2018

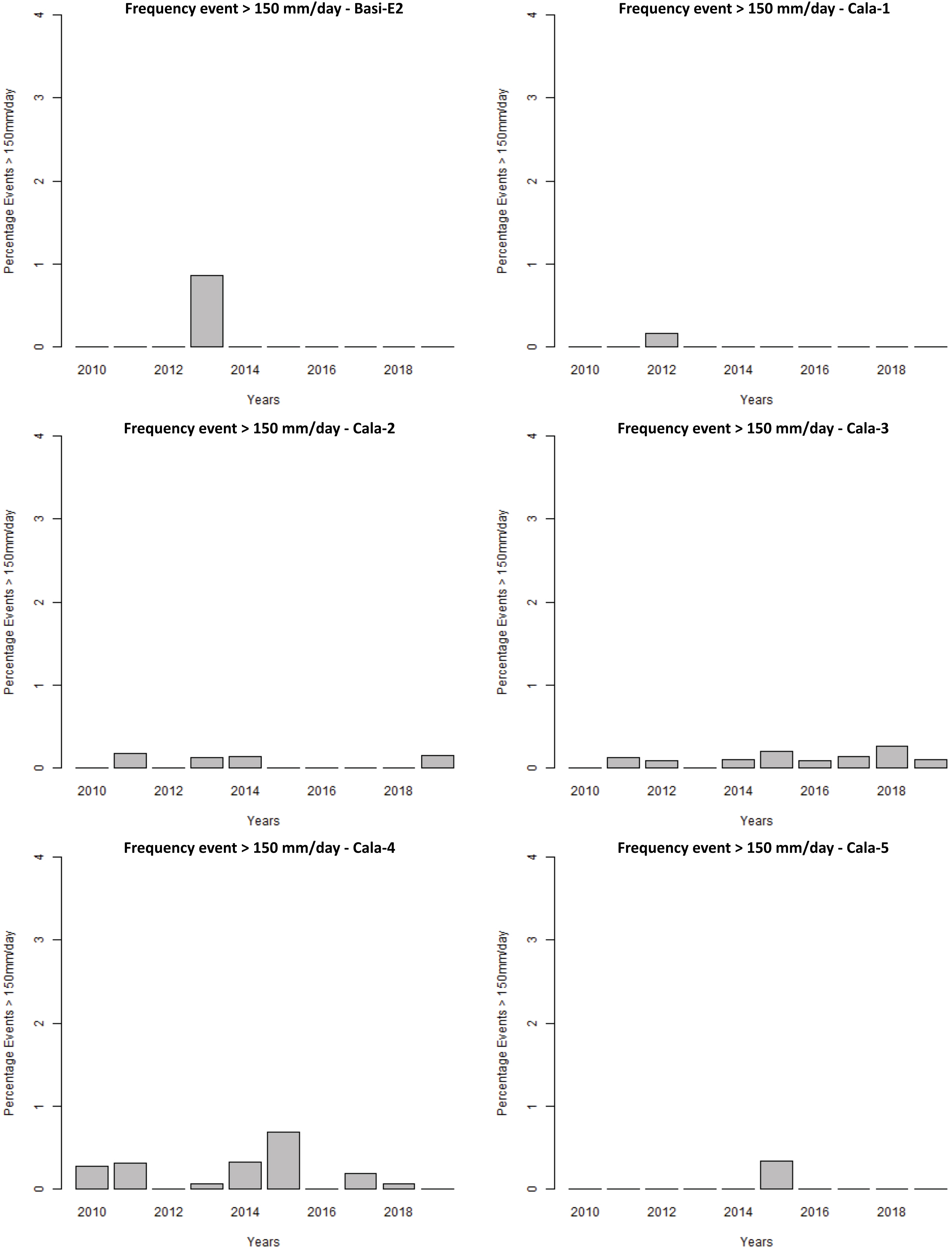
Frequency event > 150 mm/day - Basi-E2
Frequency event > 150 mm/day - Cala-1
Frequency event > 150 mm/day - Cala-2
Frequency event > 150 mm/day - Cala-3
Frequency event > 150 mm/day - Cala-4
Frequency event > 150 mm/day - Cala-5
Percentage Events > 150mm/day
Years
0
1
2
3
4
2010
2012
2014
2016
2018

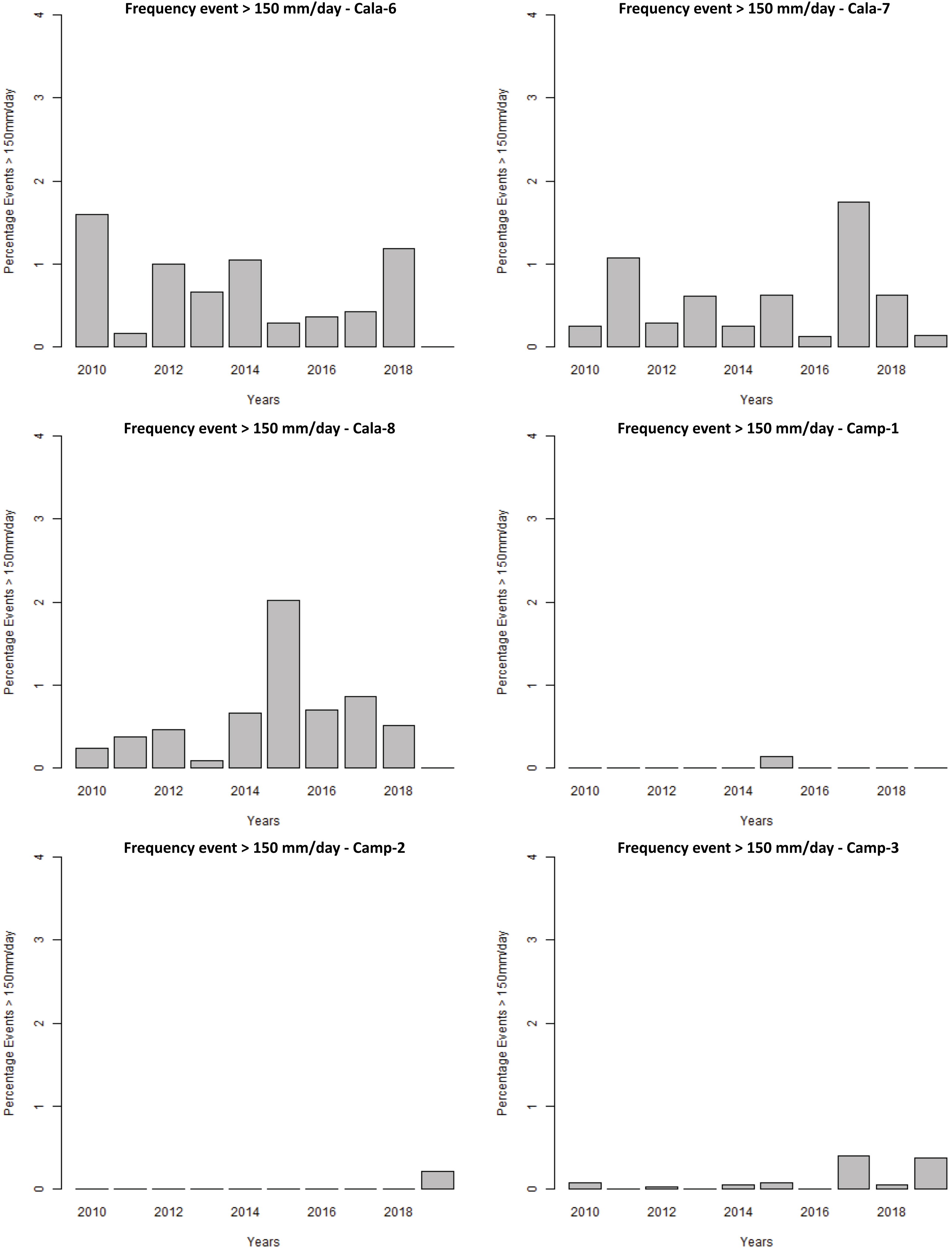
Frequency event > 150 mm/day - Cala-6
Frequency event > 150 mm/day - Cala-7
Frequency event > 150 mm/day - Cala-8
Frequency event > 150 mm/day - Camp-1
Frequency event > 150 mm/day - Camp-2
Frequency event > 150 mm/day - Camp-3
Percentage Events > 150mm/day
Years
0
1
2
3
4
2010
2012
2014
2016
2018

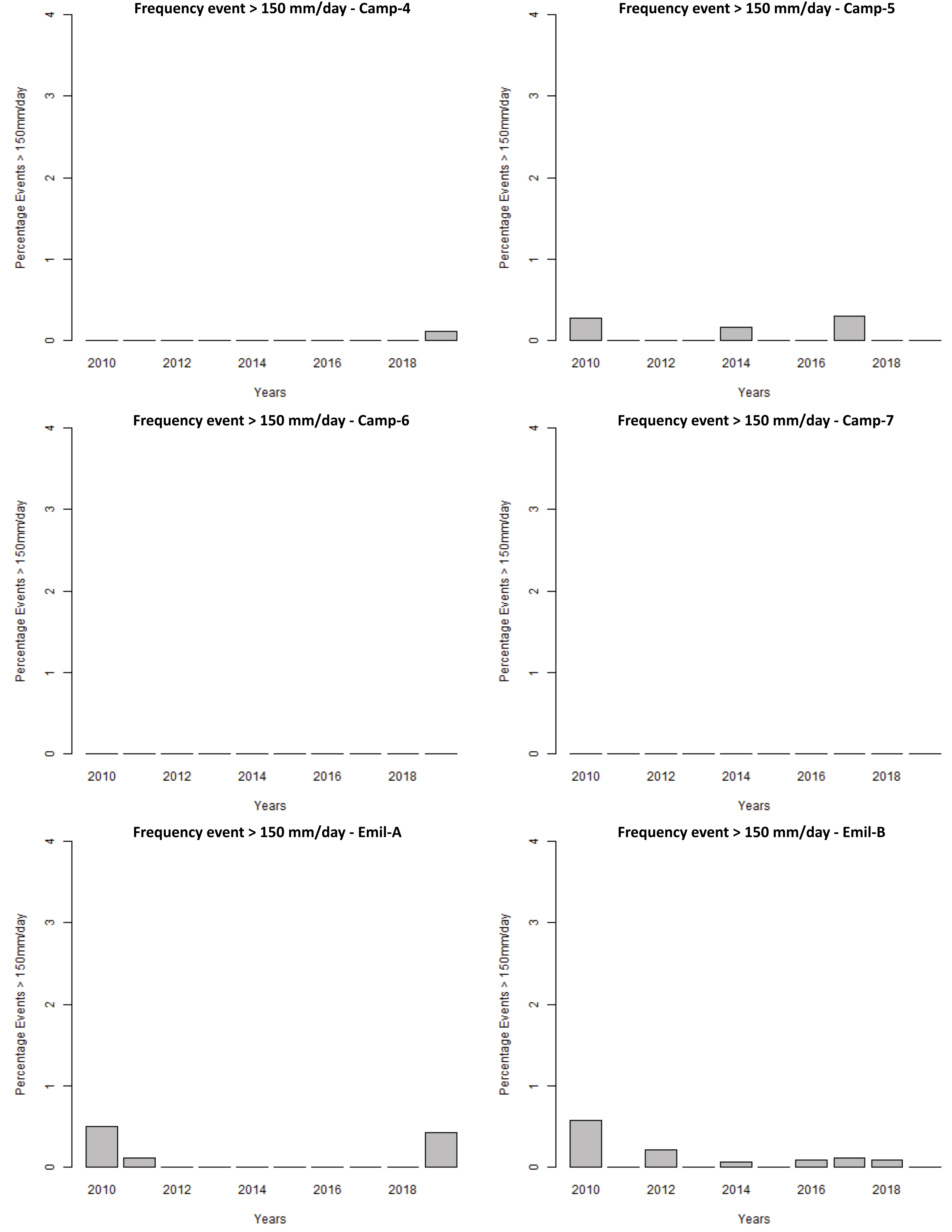
Frequency event > 150 mm/day - Camp-4
Frequency event > 150 mm/day - Camp-5
Frequency event > 150 mm/day - Camp-6
Frequency event > 150 mm/day - Camp-7
Frequency event > 150 mm/day - Emil-A
Frequency event > 150 mm/day - Emil-B
Percentage Events > 150mm/day
Years
0
1
2
3
4
2010
2012
2014
2016
2018

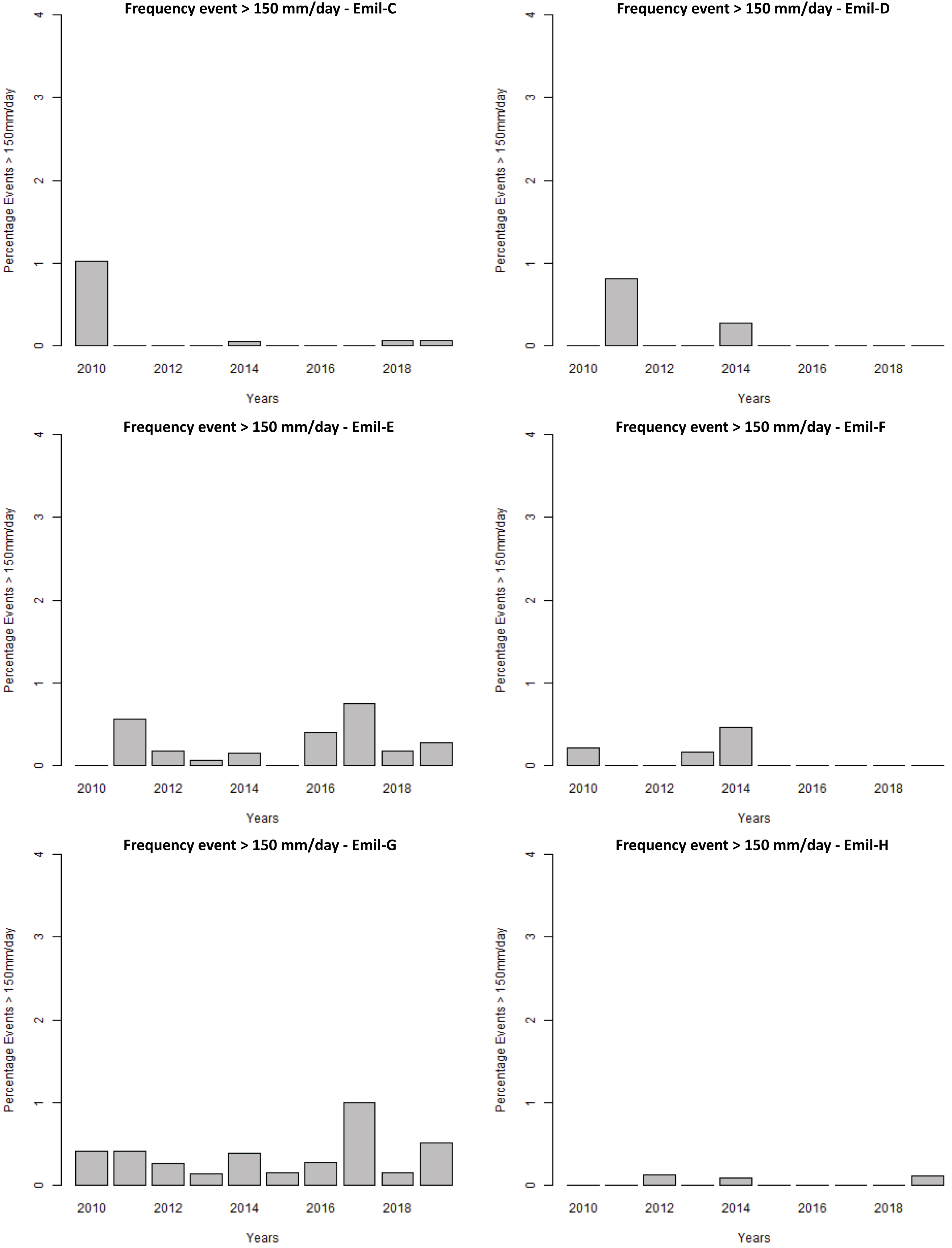
Frequency event > 150 mm/day - Emil-C
Frequency event > 150 mm/day - Emil-D
Frequency event > 150 mm/day - Emil-E
Frequency event > 150 mm/day - Emil-F
Frequency event > 150 mm/day - Emil-G
Frequency event > 150 mm/day - Emil-H
Percentage Events > 150mm/day
Years
0
1
2
3
4
2010
2012
2014
2016
2018

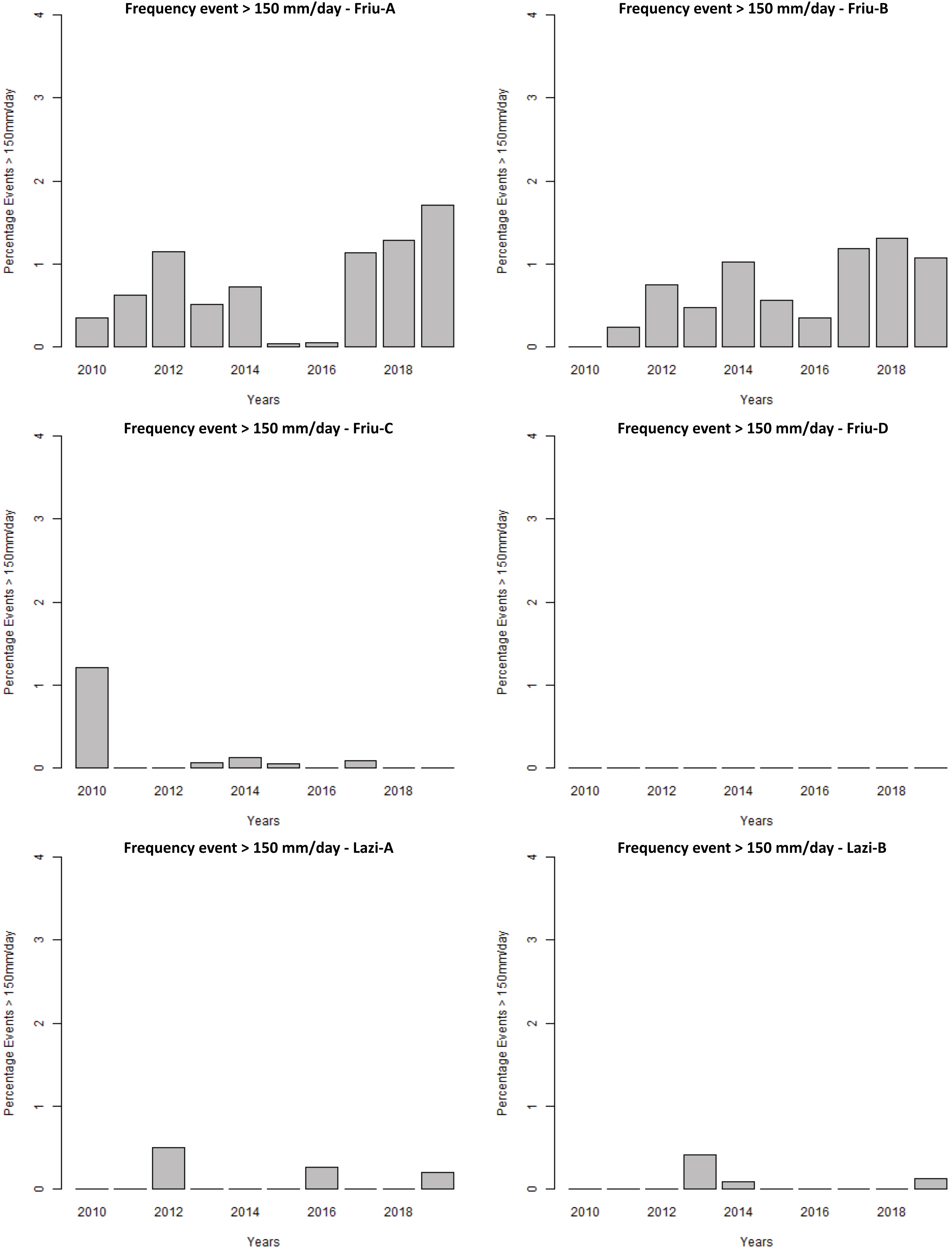
Frequency event > 150 mm/day - Friu-A
Frequency event > 150 mm/day - Friu-B
Frequency event > 150 mm/day - Friu-C
Frequency event > 150 mm/day - Friu-D
Frequency event > 150 mm/day - Lazi-A
Frequency event > 150 mm/day - Lazi-B
Percentage Events > 150mm/day
Years
0
1
2
3
4
2010
2012
2014
2016
2018

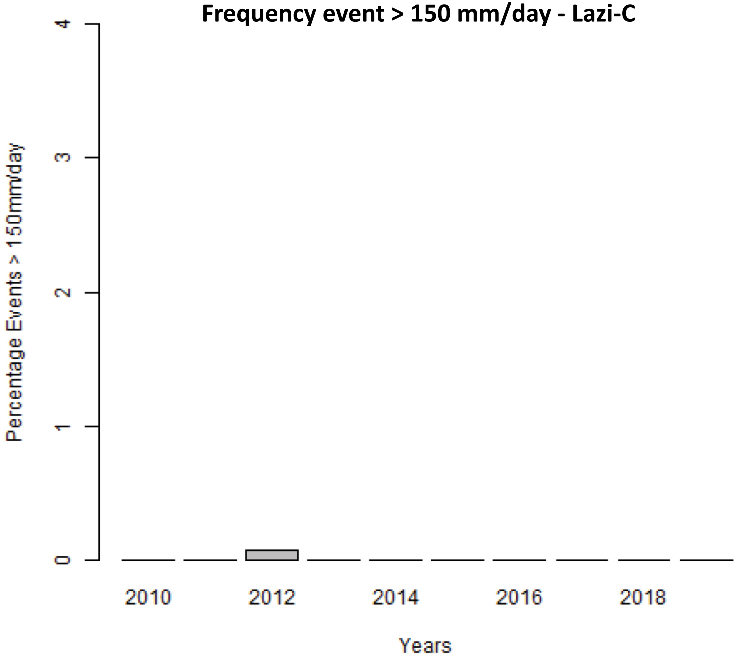
Frequency event > 150 mm/day - Lazi-C
Percentage Events > 150mm/day
Years
0
1
2
3
4
2010
2012
2014
2016
2018

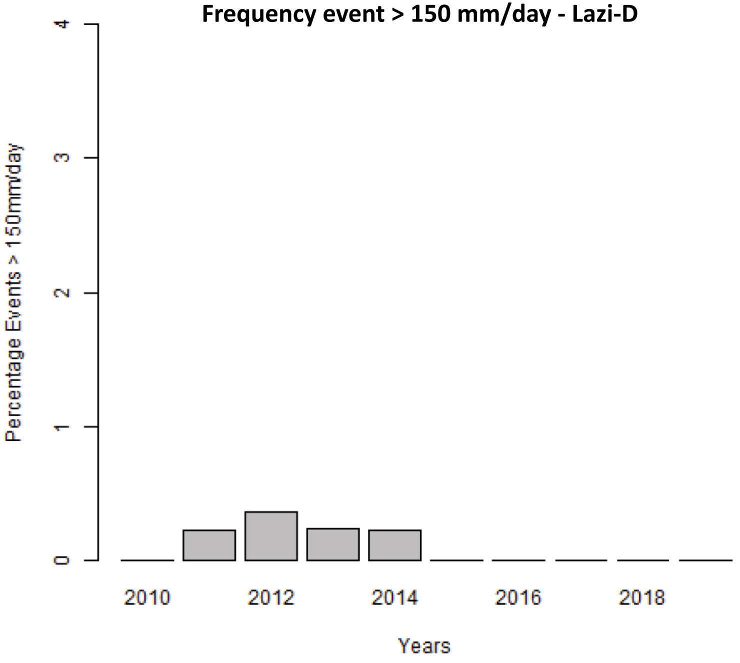
Frequency event > 150 mm/day - Lazi-D
Percentage Events > 150mm/day
Years
0
1
2
3
4
2010
2012
2014
2016
2018

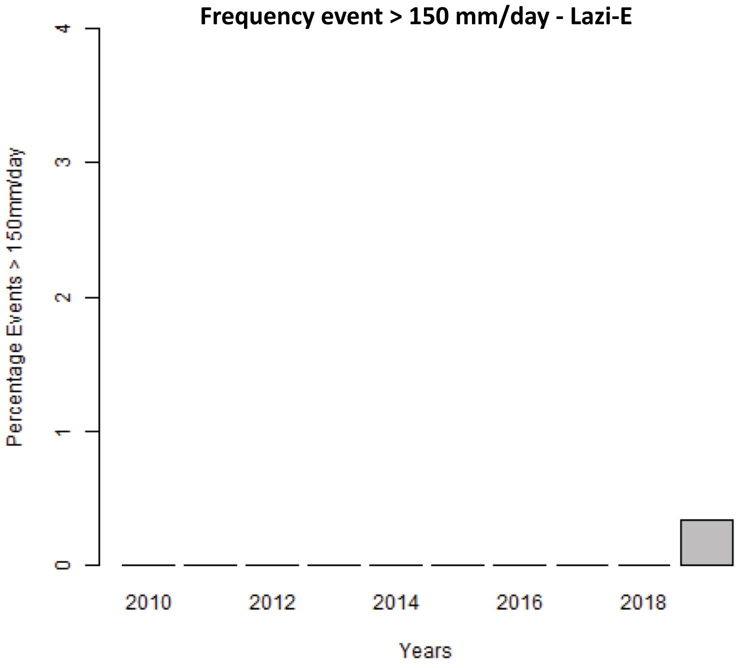
Frequency event > 150 mm/day - Lazi-E
Percentage Events > 150mm/day
Years
0
1
2
3
4
2010
2012
2014
2016
2018

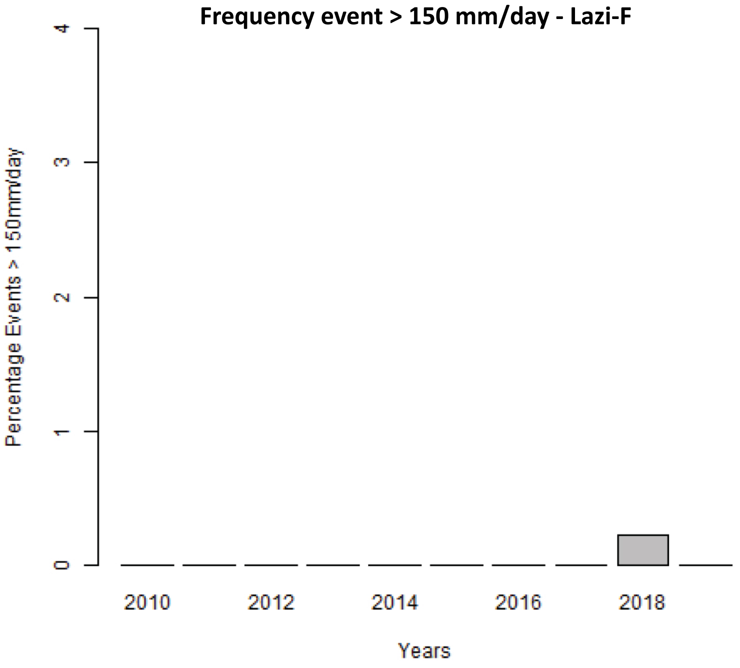
Frequency event > 150 mm/day - Lazi-F
Percentage Events > 150mm/day
Years
0
1
2
3
4
2010
2012
2014
2016
2018

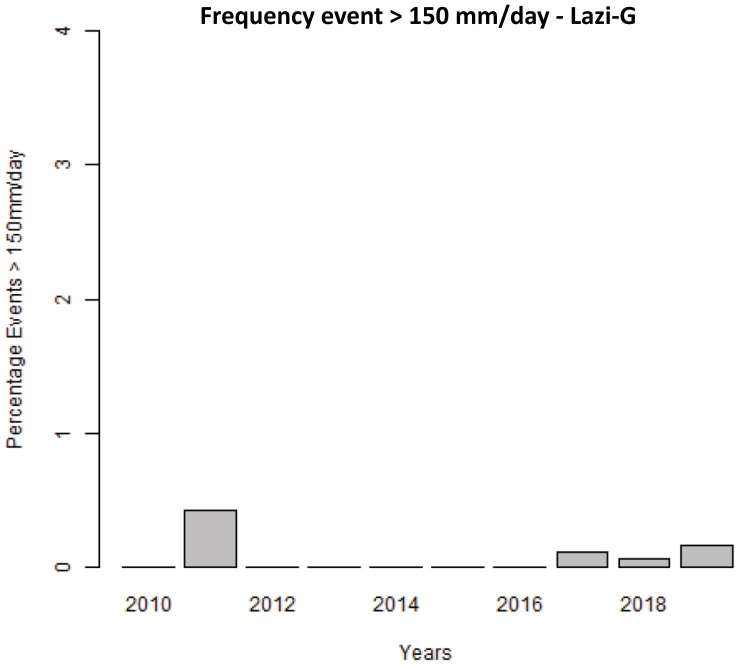
Frequency event > 150 mm/day - Lazi-G
Percentage Events > 150mm/day
Years
0
1
2
3
4
2010
2012
2014
2016
2018

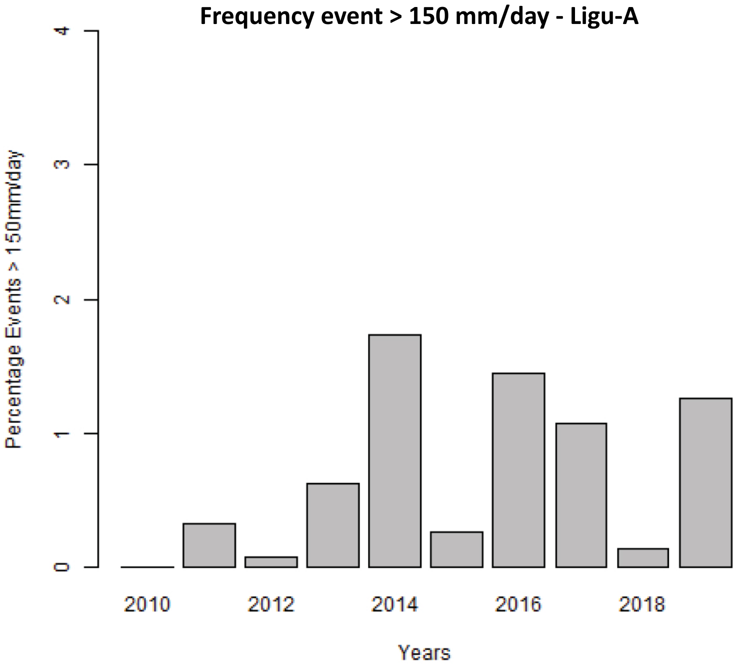
Frequency event > 150 mm/day - Ligu-A
Percentage Events > 150mm/day
Years
0
1
2
3
4
2010
2012
2014
2016
2018

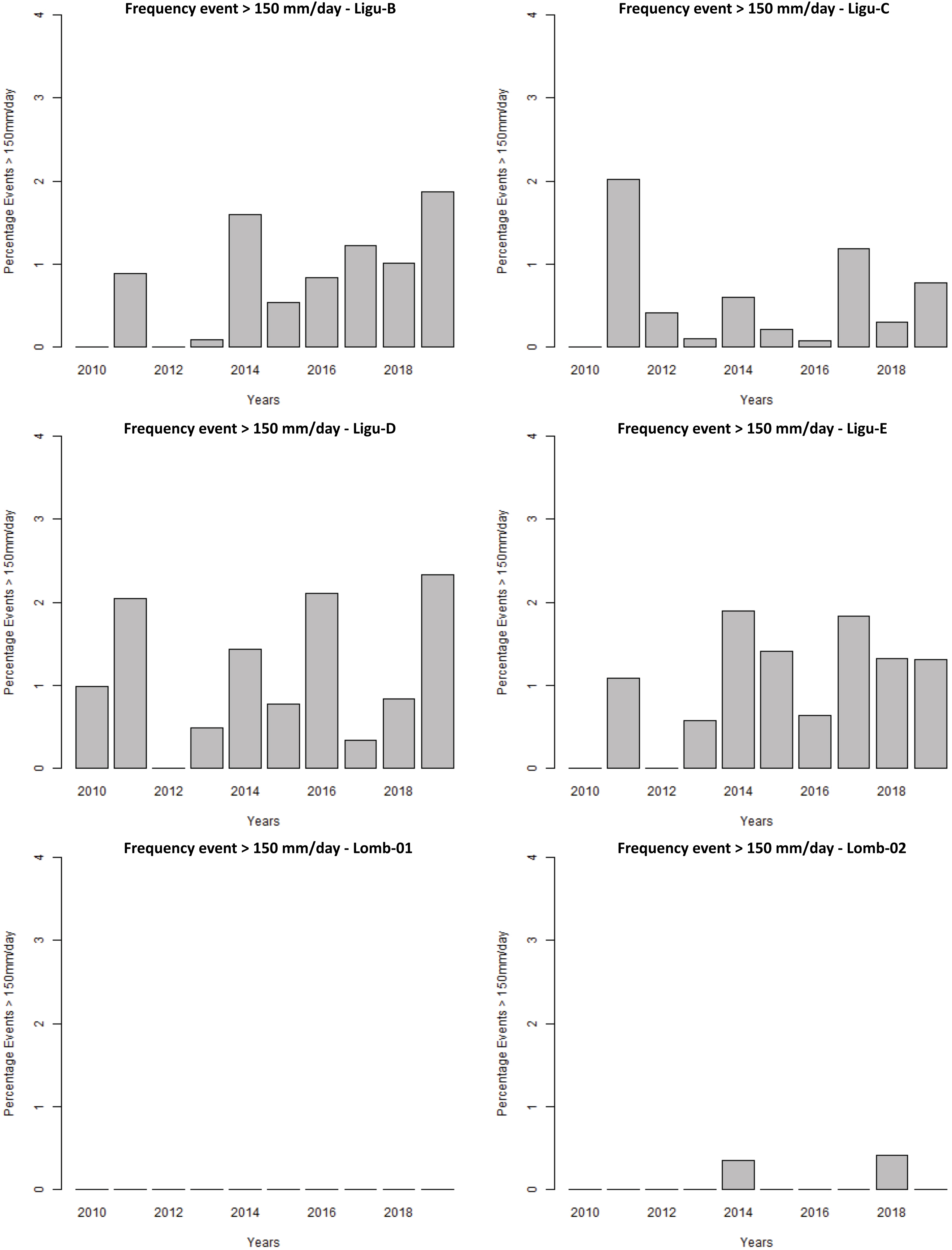
Frequency event > 150 mm/day - Ligu-B
Frequency event > 150 mm/day - Ligu-C
Frequency event > 150 mm/day - Ligu-D
Frequency event > 150 mm/day - Ligu-E
Frequency event > 150 mm/day - Lomb-01
Frequency event > 150 mm/day - Lomb-02
Percentage Events > 150mm/day
Years
0
1
2
3
4
2010
2012
2014
2016
2018
